# Impact of the national home safety equipment scheme ‘Safe At Home’ on hospital admissions for unintentional injury in children under 5: a controlled interrupted time series analysis

**DOI:** 10.1136/jech-2021-216613

**Published:** 2021-06-22

**Authors:** Trevor Hill, Carol Coupland, Denise Kendrick, Matthew Jones, Ashley Akbari, Sarah Rodgers, Michael Craig Watson, Edward Tyrrell, Sheila Merrill, Elizabeth Orton

**Affiliations:** 1 Division of Primary Care, School of Medicine, University of Nottingham, Nottingham, UK; 2 Medical School, Swansea University, Swansea, UK; 3 Public Health and Policy, University of Liverpool, Liverpool, UK; 4 Institute of Health Promotion and Education, Lichfield, UK; 5 Royal Society for the Prevention of Accidents (RoSPA), Edgbaston, UK

**Keywords:** accidents, epidemiology, injury, public health

## Abstract

**Background:**

Unintentional home injuries are a leading cause of preventable death in young children. Safety education and equipment provision improve home safety practices, but their impact on injuries is less clear. Between 2009 and 2011, a national home safety equipment scheme was implemented in England (Safe At Home), targeting high-injury-rate areas and socioeconomically disadvantaged families with children under 5. This provided a ‘natural experiment’ for evaluating the scheme’s impact on hospital admissions for unintentional injuries.

**Methods:**

Controlled interrupted time series analysis of unintentional injury hospital admission rates in small areas (Lower Layer Super Output Areas (LSOAs)) in England where the scheme was implemented (intervention areas, n=9466) and matched with LSOAs in England and Wales where it was not implemented (control areas, n=9466), with subgroup analyses by density of equipment provision.

**Results:**

57 656 homes receiving safety equipment were included in the analysis. In the 2 years after the scheme ended, monthly admission rates declined in intervention areas (−0.33% (−0.47% to −0.18%)) but did not decline in control areas (0.04% (−0.11%–0.19%), p value for difference in trend=0.001). Greater reductions in admission rates were seen as equipment provision density increased. Effects were not maintained beyond 2 years after the scheme ended.

**Conclusions:**

A national home safety equipment scheme was associated with a reduction in injury-related hospital admissions in children under 5 in the 2 years after the scheme ended. Providing a higher number of items of safety equipment appears to be more effective in reducing injury rates than providing fewer items.

## Introduction

Unintentional injuries in children aged under 5 are a leading cause of preventable death and a major cause of ill health, disability and health service resource use.^1^ Every year in England, they are responsible for approximately 370 000 emergency department attendances, 40 000 hospital admissions and 55 deaths.^2^ The majority of these injuries occur in the home, most commonly arising from falls, poisoning, choking, suffocation and strangulation, burns and scalds and drowning.^2^ Such injuries disproportionately affect the more disadvantaged, with a 38% higher hospital admission rate for children from the most deprived compared with the least deprived areas.^2^

Systematic overviews, reviews and meta-analyses have explored whether education, with or without the provision of home safety equipment, improves safety behaviours, increases safety equipment use and reduces childhood injuries. They conclude that these interventions are effective in improving some safety behaviours (safe storage of medicines and cleaning products, reducing hot tap water temperature, fire escape planning, reducing baby-walker use and availability of emergency contact numbers) and use of some items of safety equipment (smoke alarms, safety gates and socket covers) but found insufficient evidence that home safety interventions reduce injuries.^3–12^ However, several more recent small and uncontrolled studies have demonstrated injury reductions associated with education and provision of safety equipment.^13 14^

The Safe At Home (SAH) National Home Safety Equipment Scheme (https://www.rospa.com/home-safety/advice/safe-at-home), delivered between 1 April 2009 and 31 March 2011, was a major time-limited initiative aimed at reducing unintentional injury through providing home safety equipment and advice to disadvantaged families with children aged under 5 who were receiving means-tested state financial support.^15^ Designed and implemented by the Royal Society for the Prevention of Accidents (RoSPA) on behalf of the Department for Education, the £11 million programme was delivered by service providers in 130 participating local authority areas with higher than national hospital admission rates for injury in the under 5s. Over 66 000 families received safety equipment through the scheme, and over five times as many families received information and support. The scheme included training for staff delivering the scheme, home risk assessment, advice and education for parents and free provision and installation of safety equipment including safety gates, fireguard, window restrictors, non-slip bath/shower mat, kitchen cupboard locks, corner cushions and blind cord shorteners. The home safety equipment items selected for the scheme were chosen using the best available evidence of effectiveness.

A previous evaluation of the SAH scheme showed that it reached families at higher risk of child injuries,^16^ and parents reported high levels of satisfaction,^17^ equipment use and other safety behaviours.^16^ However, this evaluation did not include assessment of the effect on child injuries. The availability of routinely recorded hospital utilisation data provides a unique opportunity to evaluate the impact of the SAH national home safety intervention on child injury occurrence. We present an evaluation of the impact of the SAH scheme on hospital admission rates for unintentional injury among children under 5. A cost-effectiveness analysis of the SAH scheme is being undertaken and will be published elsewhere.

## Methods

### Study design

We conducted a controlled interrupted time series (CITS) study using hospital admission data for unintentional injury in children aged under 5 for areas in England where the SAH scheme was implemented (intervention areas) and in England and Wales where it was not implemented (matched control areas). We selected hospital admissions as these data are recorded consistently across all hospitals in England and Wales using International Classification of Diseases 2010 codes. It was not possible to use emergency department data due to multiple coding systems used with inconsistent implementation across hospitals.

### Data included in the study

RoSPA provided anonymised SAH safety equipment fitting data (type and quantity by postcode of home receiving equipment). Hospital admission data for England (Hospital Episodes Statistics (HES)) were obtained from NHS Digital and for Wales (Patient Episode Database for Wales (PEDW)) were obtained from the Secure Anonymised Information Linkage (https://saildatabank.com/) Databank, for January 2004 to December 2015. Midyear population estimates for children aged 0–4 in England and Wales and rural/urban classification were downloaded from the Office for National Statistics. We used the Townsend Deprivation Score as a measure of material deprivation. This is an area-based composite score of counts within an area of unemployment, car ownership, overcrowding in households and households not owner-occupied. Townsend Scores were downloaded from the UK Data Service. Data were stored securely on the Secure eResearch Platform (https://serp.ac.uk) held at Swansea University and accessed via secure, remote login.

### Selection of intervention and control areas

RoSPA safety equipment data at postcode level were mapped to Lower Layer Super Output Areas (LSOA) to comply with data protection legislation. LSOAs sit within local authority boundaries and have a minimum population of 1000 and a mean of 1500. LSOAs within local authorities that implemented SAH were identified as intervention LSOAs, and those in local authorities that did not implement SAH were identified as control LSOAs. Welsh data were added to increase availability of control LSOAs with high injury rates, and more socioeconomically disadvantaged areas as SAH was targeted at areas in England with the highest injury rates, which are also more socioeconomically disadvantaged. The process of selection of intervention and control areas for the analysis, and reasons for exclusion of LSOAs are shown in online supplemental material figure S1.

10.1136/jech-2021-216613.supp1 Supplementary data

### Matching of intervention and control areas

Each intervention LSOA was matched to one control LSOA using 1:1 nearest neighbour matching using a propensity score.^18–20^ Propensity scores were generated using logistic regression including LSOA 5-year baseline (2004–2008) injury rates, rurality and deprivation scores. The matching process was repeated for a secondary analysis with only equipment-preventable injuries included because these injury rates, and hence propensity scores, differed from those for the main analysis.

### Injury admission rates

The primary outcome was the hospital admission rate for unintentional injuries. Only admissions (spells of care) coded as unintentional injuries plausibly occurring in the home in children aged 0–4 years old between 2004 and 2015 were extracted from HES and PEDW data sources. Admissions for intentional injuries, those likely to have occurred outside the home (eg, transport accidents, assault and force of nature) and undetermined or unspecified injury codes were excluded. Our secondary outcome was admissions that were plausibly preventable by the safety equipment provided (equipment-preventable injuries; see online supplemental material 2 Excel document for full code lists). This more restricted code list was agreed by expert consensus within the research team, drawing on expertise from primary care, public health and injury researchers. Admission rates were aggregated across all LSOAs by month of admission across the whole study period (2004–2015).

10.1136/jech-2021-216613.supp2 Supplementary data

### Creation of equipment density categories

We explored the ‘dose effect’ of the SAH scheme by stratifying intervention LSOAs into three levels of equipment density. The number of safety items received by families was based on a risk assessment undertaken by those delivering the scheme, with families being free to decline equipment they did not want. We therefore used the total number of pieces of safety equipment provided per household, aggregated by LSOA and divided by the midyear LSOA population estimates of 0–4 year olds for 2011, to create the equipment density variable.

The resulting equipment density variable was categorised into tertiles.

### A priori selection of the interrupted time series (ITS) impact model

We selected a model measuring changes in trends but no step changes, as SAH scheme implementation occurred gradually, with sudden changes in injury admission rates unlikely.^21 22^ The start (01/04/2009) and end (31/03/2011) months of the SAH scheme were represented by binary indicator variables. A change point 2 years after the end of the SAH scheme was added to reflect when the latest fitted equipment would start to be removed (eg, safety gates are recommended to be used only for children aged up to 2 years). The time periods studied therefore were baseline (January 2004 to March 2009), implementation period (April 2009 to March 2011), first postintervention period (April 2011 to March 2013) and second postintervention period (April 2013 to December 2015).

### Statistical analysis

The analysis dataset for 12 years from January 2004 to December 2015 resulted in 144 monthly time points of aggregated LSOA data for each exposure group (intervention or control). The main analyses compared the difference in slope in admission rates between LSOAs in the intervention areas and matched control areas. Zero truncated, negative binomial regression was used to estimate the changes in the slope of the injury rate across the study time periods, between the two exposure groups. We accounted for seasonality by adding sinusoidal terms and interaction terms between these and the exposure groups.^21 23^ The model included terms for separate baseline differences in slope in the intervention and control groups and interaction terms for changes in slope between these exposure groups in the implementation and postimplementation periods. Model checking included testing for autocorrelation and viewing residual plots using a cut-off of p<0.050.^24^ See online supplemental methods for further information on the model used.

10.1136/jech-2021-216613.supp3 Supplementary data

To explore any potential ‘dose effect’ of the SAH scheme’s implementation, we replaced the binary exposure group variable with the equipment density variable, categorised into tertiles (low, medium and high density) and a separate category for control areas. We conducted all analyses in Stata/SE V.15.1.

## Results

A total of 9466 intervention and 9466 matched control LSOAs were included in the analysis; of the control LSOAs, 1144 (12.1%) were from Wales. The mean Townsend Deprivation Score was 1.95 (SD 3.71) in intervention areas and 1.13 (SD 3.14) in control areas. Intervention and control areas were equally located in rural areas 8.4%.

Intervention and control areas were similar during the baseline period in terms of population size and the proportion of male children. As expected, due to the targeting of the SAH scheme at areas with high injury rates, there were a greater number of hospital admissions in intervention areas during the baseline period, but the age distribution of children with admissions was similar in intervention and control areas (table 1).

**Table 1:** Characteristics of the study population in intervention and control areas (aggregated across all Lower Layer Super Output Areas)

Intervention areas					Matched control areas			
Study period	Baseline (Jan 04 to Mar 09)	Implementation period (Apr 09 to Mar 11)	First postintervention period (Apr 11 to Mar 13)	Second postintervention period (Apr 13 to Dec 15)	Baseline (Jan 04 to Mar 09)	Implementation period (Apr 09 to Mar 11)	First postintervention period (Apr 11 to Mar 13)	Second postintervention period (Apr 13 to Dec 15)
Mean population of 0–4 year olds	942 391	1 034 782	1 069 431	1 089 027	911 097	1 009 239	1 057 115	1 076 509
Mean percentage of population that is man	51.2	51.1	51.2	51.2	51.2	51.2	51.2	51.3
Total injury admissions by age								
0	13 618 (17.9)	6364 (18.6)	6418 (18.3)	8576 (18.0)	12 499 (18.7)	5250 (18.2)	5656 (18.3)	7748 (18.2)
1	20 235 (26.6)	9240 (26.9)	9353 (26.6)	12 702 (26.7)	17 715 (26.4)	7754 (26.9)	8324 (26.9)	10 875 (25.5)
2	17 691 (23.2)	7974 (23.3)	7897 (22.5)	10 613 (22.3)	15 330 (22.9)	6693 (23.2)	6970 (22.6)	9650 (22.6)
3	13 394 (17.6)	5870 (17.1)	6302 (18.0)	8443 (17.7)	11 487 (17.1)	4951 (17.2)	5490 (17.8)	7783 (18.2)
4	10 801 (14.2)	679 (13.6)	4953 (14.1)	7080 (14.9)	9669 (14.4)	4073 (14.1)	4323 (14.0)	6418 (15.0)
5	390 (0.5)	169 (0.5)	193 (0.6)	256 (0.5)	315 (0.5)	127 (0.4)	149 (0.5)	214 (0.5)
Total	76 129	34 296	35 116	47 670	67 015	28 848	30 912	42 688

Safety equipment was provided in 57 656 homes included in this analysis between April 2009 and March 2011. Four hundred eight LSOAs were excluded from the analysis due to incomplete data at household level on equipment provision (online supplemental figure S1). Fifty percent of homes had equipment provided by September 2010 (online supplemental figure S2). The median number of types of safety equipment provided per household was four (IQR 3–5), and the median total number of pieces of safety equipment provided per household was seven (IQR 5–10). The most commonly provided item was safety gates, with fireguards and cord winders being the least commonly provided (table 2).

**Table 2:** Safety equipment provision in intervention areas*

Type of safety equipment	Number (%) of families provided with each type of equipment	Total number of pieces of equipment† provided (% of total n fitted)
Any safety equipment	64 590	493 510 (100.0%)
Safety gate	56 894	106 986 (21.7%)
Cupboard locks	51 459	92 287 (18.7%)
Window restrictors	24 773	88 638 (18.0%)
Corner cushions	44 404	80 683 (16.3%)
Bath/shower mat	54 188	54 432 (11.0%)
Fire guard	34 009	35 852 (7.3%)
Cord winders	18 670	34 632 (7.0%)

*Equipment fitted in all Lower Layer Super Output Areas (LSOAs) prior to the selection of LSOAs for the analysis (as shown in online supplemental figure S1).

†Some pieces of safety equipment (corner cushions and cord winders) were supplied in packs containing several items, and in these instances, packs rather than individual items were counted.

### Injury admission rates

In the intervention areas, the crude annual rate of hospital admissions increased from 1512/100 000 (95% CI 1487–1538) in 2004 to 1725/100 000 (1700–1750) in 2011 and then decreased to 1517 (1494–1540) in 2015. The rate of admissions in the control areas followed a similar pattern but with less marked increases and decreases (2004: 1360 (1336–1385); 2011: 1488 (1465–1512); 2015: 1388 (1365–1410)) (table 3 and online supplemental figure S3).

**Table 3:** Crude annual hospital admission rate for all home injuries in intervention and control areas from 2004 to 2015 (financial year)

Financial year	Control areas			Intervention areas		
	Population*	Total number of injuries (95% CI)	Injury admission rate per 100 000 population (95% CI)	Population*	Total number of injuries (95% CI)	Injury admission rate per 100 000 population (95% CI)
Baseline period						
Jan 2004 to Mar 2004	217 256	2621 (2522–2723)	1206 (1161–1254)	223 609	3063 (2955–3173)	1370 (1322–1419)
Apr 2004 to Mar 2005	871 836	11 861 (11 648–12 076)	1360 (1336–1385)	898 390	13 580 (13 353–13 810)	1512 (1486–1537)
Apr 2005 to Mar 2006	884 749	12 712 (12 492–12 935)	1437 (1412–1462)	915 940	13 960 (13 729–14 194)	1524 (1499–1550)
Apr 2006 to Mar 2007	905 513	12 955 (12 733–13 180)	1431 (1406–1456)	940 047	14 790 (14 553–15 030)	1573 (1548–1599)
Apr 2007 to Mar 2008	935 937	13 201 (12 977–13 428)	1410 (1387–1435)	969 336	15 135 (14 895–15 378)	1561 (1536–1586)
Apr 2008 to Mar 2009	967 968	13 665 (13 437–13 896)	1412 (1388–1436)	1 000 231	15 601 (15 357–15 848)	1560 (1535–1584)
Implementation period						
Apr 2009 to Mar 2010	995 719	14 062 (13 831–14 296)	1412 (1389–1436)	1 024 857	16 635 (16 383–16 890)	1623 (1599–1648)
Apr 2010 to Mar 2011	1 022 758	14 786 (14 549–15 026)	1446 (1422–1469)	1 044 707	17 661 (17 401–17 923)	1690 (1666–1716)
First postintervention period						
Apr 2011 to Mar 2012	1 047 203	15 655 (15 411–15 902)	1495 (1472–1519)	1 060 430	18 419 (17 886–18 415)	1711 (1687–1737)
Apr 2012 to Mar 2013	1 067 028	15 257 (15 016–15 501)	1430 (1407–1453)	1 078 433	16 967 (16 713–17 224)	1573 (1550–1597)
Second postintervention period						
Apr 2013 to Mar 2014	1 074 547	16 003 (15 756–16 253)	1489 (1466–1513)	1 085 764	17 652 (17 393–17 914)	1626 (1602–1650)
Apr 2014 to Mar 2015	1 078 049	15 153 (14 913–15 396)	1406 (1383–1428)	1 090 098	17 375 (17 118–17 635)	1594 (1570–1618)
Apr 2015 to Dec 2015	807 802	11 532 (11 322–11 744)	1428 (1402–1454)	818 961	12 643 (12 424–12 865)	1544 (1517–1571)

*Population is the total number of 0–4 year olds for all Lower Layer Super Output Areas combined (by treatment group) using midyear population estimates from the Office for National Statistics in England and Wales.

**Figure 1:**
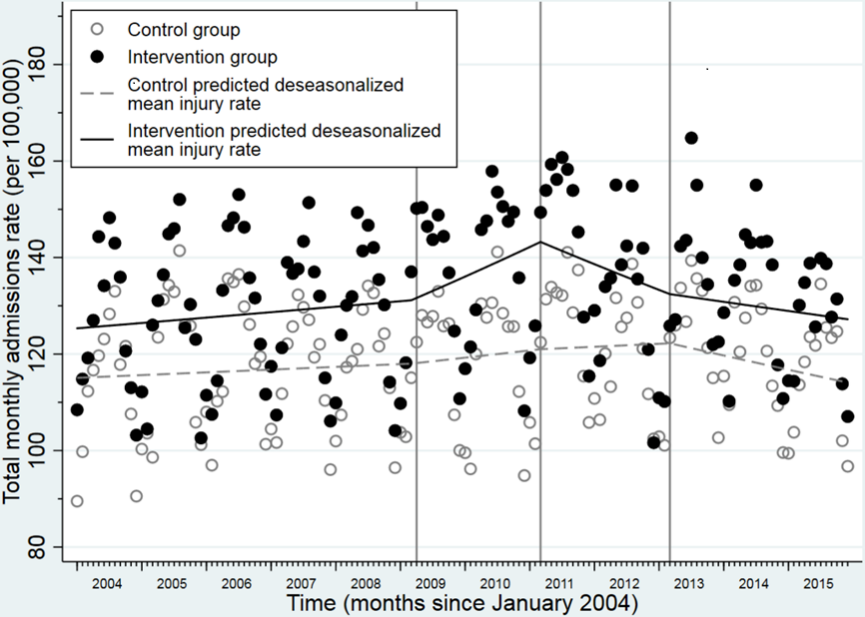
Trends in predicted deseasonalised hospital admission rates before, during and after the Safe At Home scheme in intervention and control areas.

The predicted deseasonalised monthly injury rates in the main analysis are presented in figure 1 and table 4. In the baseline period, there were similar small increases in monthly admission rates in the control (0.04% (−0.02%to 0.10%), p=0.16) and intervention areas (0.07% (0.02% to 0.13%), p=0.009, p value for difference in trends=0.42). During the implementation period, there was a small increase in admission rates in control (0.10% (−0.03% to 0.24%), p=0.14) and a greater increase in intervention areas (0.37% (0.23% to 0.50%), p<0.001, p value for difference in trends=0.007). In the first postintervention period (April 2011 to March 2013), there was a small increase in admission rates in control (0.04% (−0.11% to 0.19%), p=0.59) and a larger decrease in admission rates in the intervention areas (−0.33% (−0.47% to −0.18%), p<0.001, p value for difference in trends=0.001). By the second postintervention period (April 2013 to December 2015), there were similar decreases in admission rates in both control (−0.21% (−0.34% to −0.08%), p=0.002) and intervention areas (−0.12% (−0.25% to 0.01%), p=0.06, p value for difference in trends=0.35).

**Table 4:** Adjusted trends in predicted hospital admission rates before, during and after the Safe At Home scheme in intervention and control areas

Time point	Predicted injury hospital admission rate per 100 000 per month and 95% CI*		Difference in Trends*†
	Control areas	Intervention areas	
**Baseline period**			
January 2004	115.1 (112.5–117.7)	125.3 (122.7–128.1)	
March 2009	118.0 (115.8–120.3)	131.2 (128.7–133.6)	
Change in rate per month (%)‡	0.04 (−0.02–0.10) P=0.16	0.07 (0.02–0.13) P=0.009	0.03 (−0.05–0.11) P=0.42
**Implementation period**			
April 2009	118.15 (116.0–120.3)	131.63 (129.3–134.0)	
March 2011	121.01 (118.2–123.9)	143.24 (140.0–146.5)	
Change in rate per month (%)‡	0.10 (−0.03–0.24) P=0.14	0.37 (0.23–0.50) P<0.001	0.26 (0.07–0.46) P=0.007
**First postintervention period**			
April 2011	121.1 (118.4–123.8)	142.8 (139.7–145.9)	
March 2013	122.2 (119.5–125.0)	132.4 (129.5–135.4)	
Change in rate per month (%)‡	0.04 (−0.11–0.19) P=0.59	−0.33 (−0.47 to −0.18) P<0.001	−0.37 (−0.58 to −0.16) P=0.001
**Second postintervention period**			
April 2013	122.0 (119.4–124.6)	132.3 (129.5–135.1)	
December 2015	114.10 (110.9–117.4)	127.2 (123.7–130.8)	
Change in rate per month (%)‡	−0.21 (−0.34 to −0.08) P=0.002	−0.12 (−0.25–0.01) P=0.06	0.09 (−0.09–0.27) P=0.35

*Across all time points, within each time period.

†The difference in trends is the difference between the change in rate in intervention areas and the change in rates in the control areas.

‡The change in rate per month is derived from the incidence rate ratio and reflects the percentage change in the injury rate per month.

When stratified by equipment density, admission rates in each time period were highest in areas with highest equipment density and lowest in areas with lowest equipment density, as expected due to the SAH scheme being implemented in areas with the highest injury rates. Admission rates stratified by equipment density tertiles were similar in pattern to the main analysis (online supplemental figure S4 and table S1). In the first postintervention period, there was a dose–response relationship with a greater decrease in admission rates in the high-equipment-density areas (−0.37% per month (−0.56% to −0.19%)) compared with the medium-equipment-density (−0.32% per month (−0.52% to −0.12%)) and low-equipment-density (−0.27% per month (−0.48% to −0.07%)) areas (p value for difference in trends between the high-equipment-density areas and the control areas=0.001).

Restricting analyses to equipment-preventable injuries resulted in lower and more variable admission rates than in the main analysis (online supplemental table S2 and figure S5). There were differences in trends in admission rates between control and intervention areas in each time period. At baseline, admission rates are increased in intervention areas but reduced slightly in control areas (p value for difference in trends=0.005). During the implementation period, admission rates increased in both control and intervention areas, with greater increases in control areas (p value for difference in trends=0.002). In the first postintervention period, admission rates in the control areas continued to increase (0.30% per month (0.07%–0.53%), but rates decreased in intervention areas (−0.28% per month (−0.51% to −0.06%) (p value for difference in trends<0.001). In the second postintervention period, admission rates in both control and intervention areas decreased, with greater decreases in control areas (p value for difference in trends<0.001). Analysis of equipment-preventable admission rates by equipment density was consistent with the main results (online supplemental tables S3–S5).

## Discussion

We used a CITS analysis of small-area data to assess the association between the implementation of a home safety equipment scheme and hospital admission due to home injuries in children under 5. We showed that monthly predicted admission rates significantly declined in intervention but not control areas following implementation. This was not maintained beyond 2 years after the scheme ended. We also found that the effect increased as equipment provision density increased, providing evidence of a ‘dose effect’.

### Comparison with the literature

Using an extremely robust study design, our finding of a significant reduction in injury admission rates in intervention but not control areas in the first 2 years after the scheme ended is consistent with findings of several other studies evaluating provision of home safety equipment.^13 14 25 26^ We cannot compare the magnitude of the reduction in injury occurrence in our study with these studies as they either reported emergency department attendance rates as opposed to hospital admission rates^13 14 25^ or any medically attended equipment-preventable injury, as opposed to equipment-preventable hospital admission.^26^ Furthermore, none of these studies reported injury outcomes in the 2 years after the intervention ended. Our findings contrast with two randomised controlled trials (RCTs) evaluating the provision of home safety education and equipment for families with children under 5, which did not find significant reductions in injury-related hospital admissions, despite improvements in home safety practices.^27 28^ However, unlike our study, neither trial had hospital admissions as the primary outcome; hence, both were underpowered to detect significant reductions. In addition, only 38% of families received safety equipment in one trial,^27^ and the uptake of the safety equipment component of the intervention was not reported in the second trial.^28^

### Strengths and limitations

The size and length of the follow-up are particular strengths of this study. We analysed nearly 60 000 intervention households in a scheme costing £11 million ($13.7 million and €12.1 million) over an 11-year period, prior to, during and after the scheme was implemented. While an RCT would provide clearer information about causal relationships between the equipment and injuries prevented, due to the extremely large sample size required, it is unlikely that such an RCT would ever be funded. This means that a ‘natural experiment’ provides the best available evidence of effectiveness, particularly as injury admissions are relatively rare, with only 2%–3% of children in the two RCTs mentioned above having a hospital admission over a 2-year period. In addition, using ‘real-world’ data improves the external validity of the study.^21^

In our study, hospital admission data were not provided at postcode level due to data protection constraints. This likely reduced the potential to detect an effect leading to an underestimation of the effect magnitude. However, the data on equipment provision were well documented at postcode level, providing granularity of information that allowed us to assess the ‘dose effect’. We hypothesised that if the scheme is effective, there would be a steeper decline in injury-related admissions in areas with higher compared with lower equipment density.^29^ While an ITS analysis cannot prove causation, our findings of a dose–response effect strengthen the evidence that providing and fitting home safety equipment are associated with reduced injury admission rates, providing further evidence of causation using Bradford Hill criteria.^30^

An ITS analysis as undertaken here is particularly useful for evaluating public health interventions that happen over a defined period.^21^ We were not able to demonstrate direct causality with this method, and reduction in injuries could have been at least partly due to other changes such as improved supervision rather than the installation of equipment. However, our ITS did include a control group, which has been shown to improve the validity of the analysis by accounting for several sources of bias.^31–33^ We matched on preintervention injury rates, deprivation and rurality; specified the impact model a priori, controlled for seasonality and took account of overdispersion and assessed for autocorrelation, all of which are recommended approaches.^21^

In the preimplementation period, hospital admission rates increased and continued to do so during the implementation period. While it is unclear what caused this, it reflects the national increase in emergency department attendance during this time,^34^ possibly related to the impact of the financial crisis and subsequent austerity measures that disproportionately affected disadvantaged communities and child health.^35 36^

There were some limitations of the study relating to the implementation of the scheme. Our analysis included all families in intervention LSOAs whether they received equipment or not (akin to an intention to treat analysis). This will have included those not eligible for equipment and those not wanting equipment, although some of these families may have received home safety information. Our LSOA-level effect sizes are therefore likely to be conservative estimates of the effect of providing safety equipment. Families who were eligible for safety equipment did not always choose to have it fitted. Reasons included aesthetics, smaller homes and difficulties in being able to have fixtures attached to the walls of rented accommodation, and sometimes, families were not contactable when fitting was due (SM, RoSPA, *personal communications* 2017). Consequently, some equipment-preventable injuries may have occurred despite the availability of the SAH scheme.

Further, this study only quantifies the impact of the scheme on more serious injuries requiring hospital admission and does not provide an indication of the total impact of the scheme including emergency department attendances, primary care attendances or non-medically attended injury. It was not possible to use emergency department data because reasons for attendance are poorly recorded,^37^ nor primary care data as this frequently does not include injury mechanisms.^38^ These problems further highlight the need to design routine data collection systems that link across multiple health, social care and other sectors.^39^ Such systems need to protect data privacy while retaining the ability to link datasets for the evaluation of interventions received at the household level^40^ improving the resolution of studies.^41^ This will increase the likelihood that effective interventions are rolled out for those most in need, promoting equity.

In conclusion, this study provides evidence of the effectiveness of home safety equipment schemes on preventing serious injuries that require hospital admission. This effect diminishes with time once schemes end and equipment ceases to be provided free of charge.

**What is already known**

- Unintentional injury in children under 5 disproportionately affects those living in the most disadvantaged circumstances. The provision of home safety equipment is known to increase its use and improve home safety practices, but there is insufficient evidence of the impact on injury rates.

**What this study adds**

- A national home safety equipment scheme was associated with a significant decrease in injury admissions in intervention areas compared with control areas. The effect was stronger where equipment provision was greatest. These results support the commissioning of home safety equipment schemes by healthcare commissioners, such as Clinical Commissioning Groups in England.

## Data Availability

Data may be obtained from a third party and are not publicly available. The data used in this study are available in the SAIL Databank at Swansea University, Swansea, UK, but as restrictions apply, they are not publicly available. All proposals to use SAIL data are subject to review by an independent Information Governance Review Panel (IGRP). Before any data can be accessed, approval must be given by the IGRP. The IGRP gives careful consideration to each project to ensure proper and appropriate use of SAIL data. When access has been granted, it is gained through a privacy protecting safe haven and remote access system referred to as the SAIL Gateway. SAIL has established an application process to be followed by anyone who would like to access data via SAIL at https://www.saildatabank.com/application-process. The HES Data (copyright 2021) was reused with the permission of the Health and Social Care Information Centre. All rights reserved. Data sharing agreement number DARS-NIC-50919-D5R5D-V1.4.
