# Supplementary material for: Impact of the national home safety equipment scheme ‘Safe At Home’ on hospital admissions for unintentional injury in children under 5: a controlled interrupted time series analysis
Source: J Epidemiol Community Health. 2021 Jun 22;76(1):53–9. doi: 10.1136/jech-2021-216613 (PMC8666806; doi:10.1136/jech-2021-216613)
Supplement: Supplementary data [file jech-2021-216613supp001.pdf]

Figure S1. Selection of Lower Super Output Areas for the analysis.

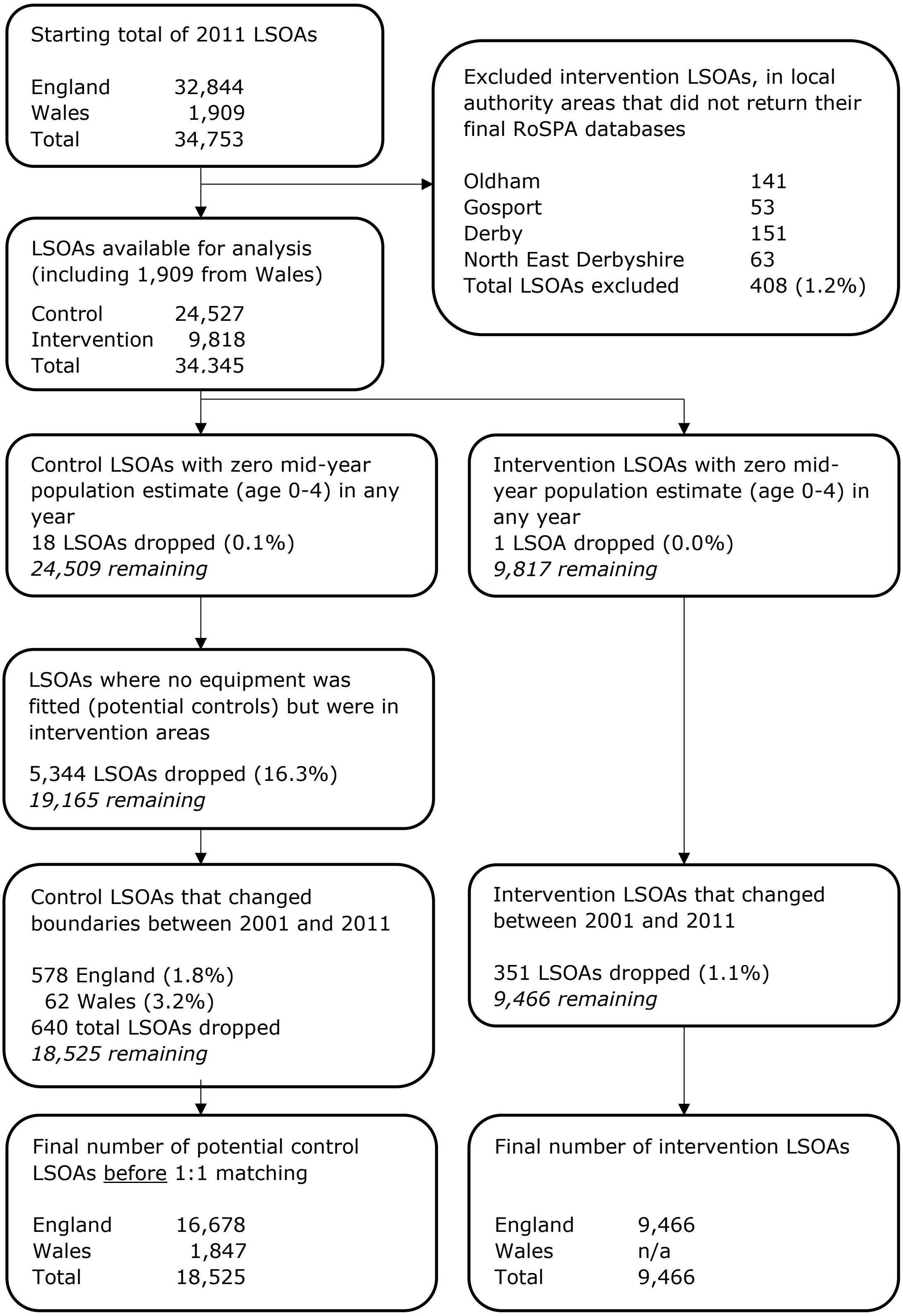

Figure S2: Cumulative percentage of households having safety equipment installed by implementation month (N= 57,656 households)

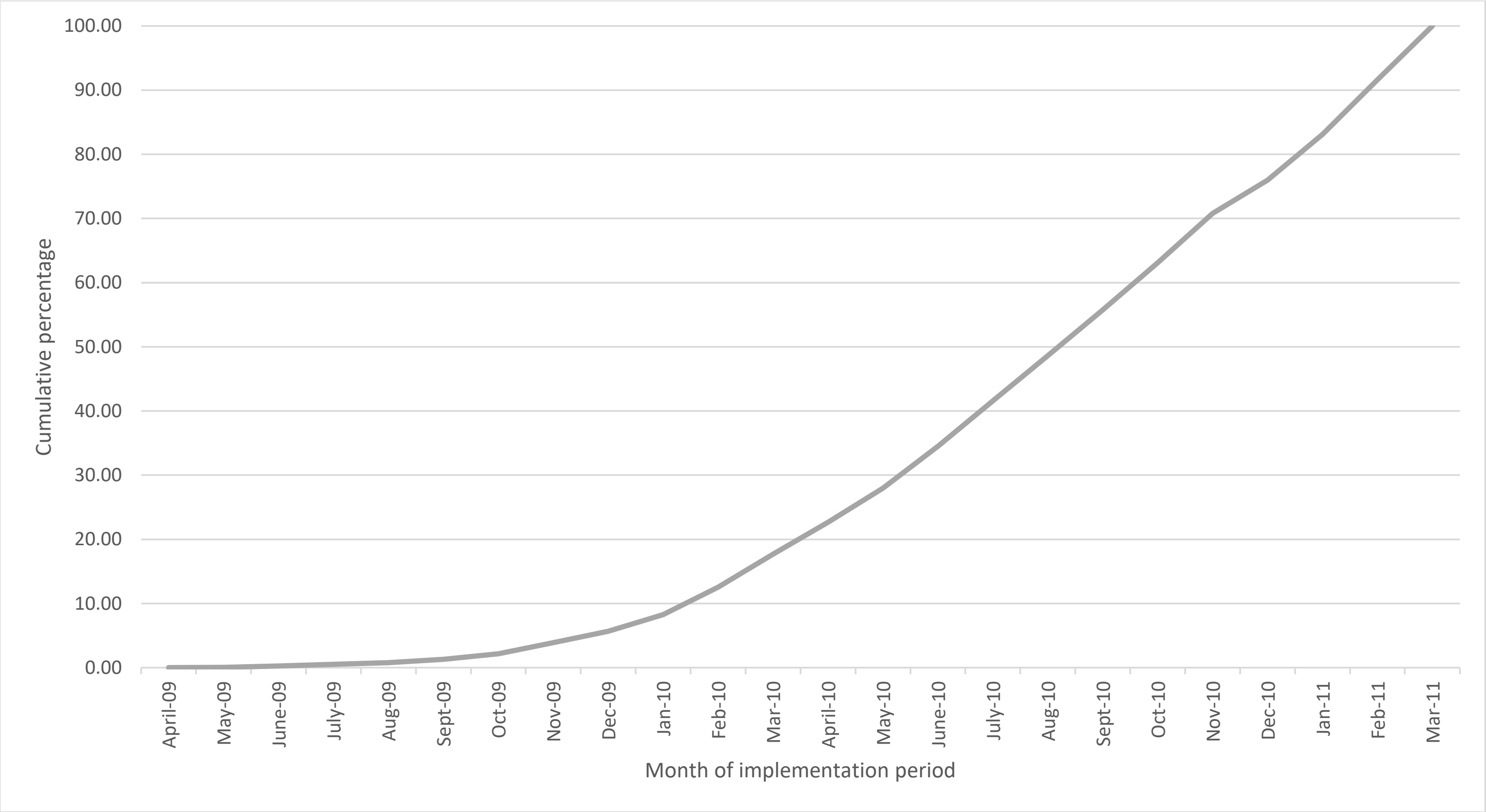

Figure S3: Crude annual hospital admission rates for all home injuries in intervention and control areas from 2004 to 2015

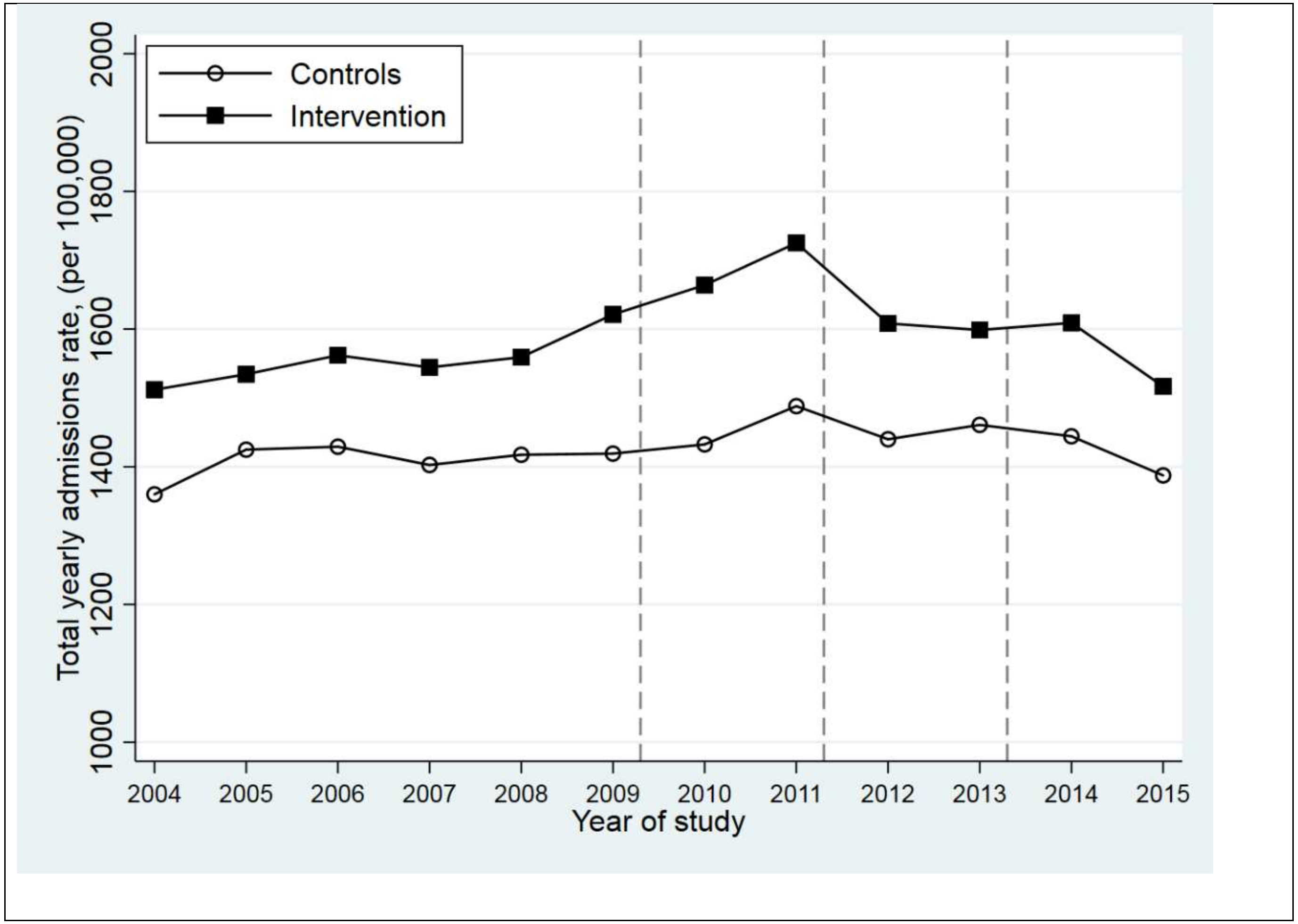

Figure S4: Trends in predicted deseasonalised hospital admission rates before, during and after the Safe At Home scheme in intervention and control areas, stratified by equipment density.

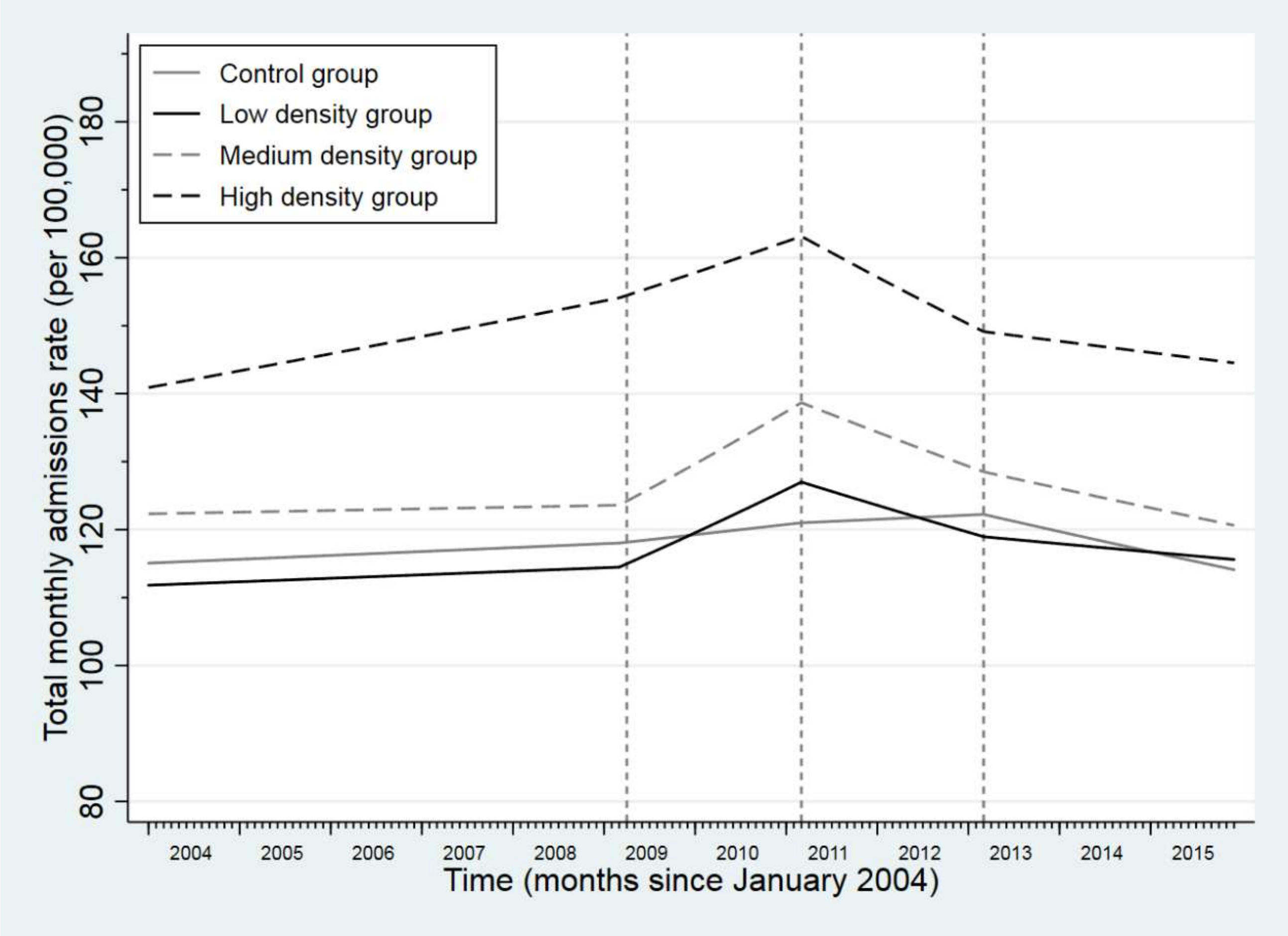

Table S1: Adjusted trends in predicted hospital admission rates according to equipment density before, during and after the Safe At Home scheme in intervention and control areas

**Time point**	**Predicted deseasonalised hospital admission rate per 100,000 per month and 95% Confidence Interval**				**Difference in Trends[1,3]**
	**Control areas**	**Intervention areas**			
		**Low equipment density**	**Medium equipment density**	**High equipment density**	
**Baseline period**					
January 2004	115.1 (112.5 – 117.8)	111.8 (108.4 – 115.4)	122.3 (118.7 – 126.1)	140.9 (137.0 – 145.0)	
March 2009	118.03 (115.7 – 120.4)	114.5 (111.5 – 117.5)	123.60 (120.4 – 126.8)	154.10 (150.5 – 157.8)	
Change in rate per month (%)[2]	0.04 (-0.02 – 0.10) P = 0.17	0.04 (-0.04 – 0.12) P = 0.34	0.02 (-0.06 – 0.09) P = 0.67	0.14 (0.07 – 0.22) P < 0.001	0.10 (0.01 – 0.20) P = 0.03
**Implementation period**					
April 2009	118.2 (116.0 – 120.4)	115.0 (112.2 – 117.9)	124.2 (121.2 – 127.3)	154.5 (151.0 – 158.0)	
March 2011	121.0 (118.1 – 124.0)	127.00 (123.1 – 131.0)	138.7 (134.4 – 143.0)	163.2 (158.5 – 167.9)	
Change in rate per month	0.10 (-0.04 – 0.25) P = 0.16	0.43 (0.25 – 0.62) P < 0.001	0.48 (0.29 – 0.66) P < 0.001	0.24 (0.07 – 0.41) P = 0.006	0.13 (-0.09 – 0.36) P = 0.24
**1st post intervention period**					
April 2011	121.1 (118.3 – 123.9)	126.7 (122.9 – 130.5)	138.2 (134.2 – 142.4)	162.6 (158.2 – 167.1)	
March 2013	122.2 (119.4 – 125.1)	119.0 (115.4 – 122.7)	128.5 (124.7 – 132.5)	149.1 (145.0 – 153.4)	
Change in rate per month	0.04 (-0.11 – 0.20) P = 0.60	-0.27 (-0.48 – -0.07) P = 0.01	-0.32 (-0.52 – -0.12) P = 0.002	-0.37 (-0.56 – -0.19) P < 0.001	-0.42 (-0.66 – -0.17) P = 0.001
**2nd post intervention period**					
April 2013	122.0 (119.3 – 124.7)	118.9 (115.4 – 122.4)	128.3 (124.6 – 132.0)	149.0 (145.0 – 153.1)	
December 2015	114.1 (110.8 – 117.5)	115.6 (111.2 – 120.1)	120.6 (116.1 – 125.3)	144.5 (139.5 – 149.8)	
Change in rate per month	-0.21 (-0.34 – -0.08) P = 0.002	-0.09 (-0.26 – 0.09) P = 0.33	-0.19 (-0.37 – -0.02) P = 0.03	-0.09 (-0.26 – 0.07) P = 0.25	0.11 (-0.10 – 0.32) P = 0.29

[1] Between the high density group and the controls

[2] The change in rate per month is the incidence rate ratio (IRR) and reflects the percentage change in the injury rate per month.

[3] The difference in trends is the difference between the change in rate in intervention areas and the change in rates in the control areas.

Table S2: Adjusted trends in predicted hospital admission rates for equipment preventable injuries, before, during and after the Safe At Home scheme in intervention and control areas

Time point	Predicted deseasonalised hospital admission rate per 100,000 per month and 95% Confidence Interval		Difference in Trends[1,3]
**Baseline period**	Control	Intervention	
January 2004	31.70 (30.60 – 32.84)	33.65 (32.53 – 34.82)	
March 2009	31.07 (30.15 – 32.02)	36.81 (35.77 – 37.87)	
Change in rate per month (%)[2]	-0.03 (-0.12 – 0.06)<br>P = 0.48	0.14 (0.06 – 0.23)<br>P = 0.001	0.18 (0.05 – 0.30)<br>P = 0.005
**Implementation period**			
April 2009	31.25 (30.37 – 32.16)	36.85 (35.87 – 37.87)	
March 2011	35.78 (34.53 – 37.08)	37.92 (36.63 – 39.27)	
Change in rate per month	0.59 (0.38 – 0.80)<br>P < 0.001	0.12 (-0.08 – 0.33)<br>P = 0.24	-0.46 (-0.76 – -0.17)<br>P = 0.002
**1st post intervention period**			
April 2011	35.89 (34.70 – 37.12)	37.82 (36.59 – 39.09)	
March 2013	38.46 (37.19 – 39.78)	35.42 (34.23 – 36.65)	
Change in rate per month	0.30 (0.07 – 0.53)<br>P = 0.01	-0.28 (-0.51 – -0.06)<br>P = 0.01	-0.59 (-0.91 – -0.26)<br>P < 0.001
**2nd post intervention period**			
April 2013	38.18 (36.97 – 39.43)	35.35 (34.22 – 36.52)	
December 2015	30.10 (28.81 – 31.45)	33.24 (31.83 – 34.71)	
Change in rate per month	-0.74 (-0.94 – -0.55)<br>P < 0.001	-0.19 (-0.39 – 0.004)<br>P = 0.05	0.55 (0.27 – 0.83)<br>P < 0.001

[1] Across all time points, within each time period.
[2] The change in rate per month is the incidence rate ratio (IRR) and reflects the percentage change in the injury rate per month.
[3] The difference in trends is the difference between the change in rate in intervention areas and the change in rates in the control areas.

Figure S5: Crude annual hospital admission rates for equipment preventable injuries, in intervention and control areas from 2004 to 2015

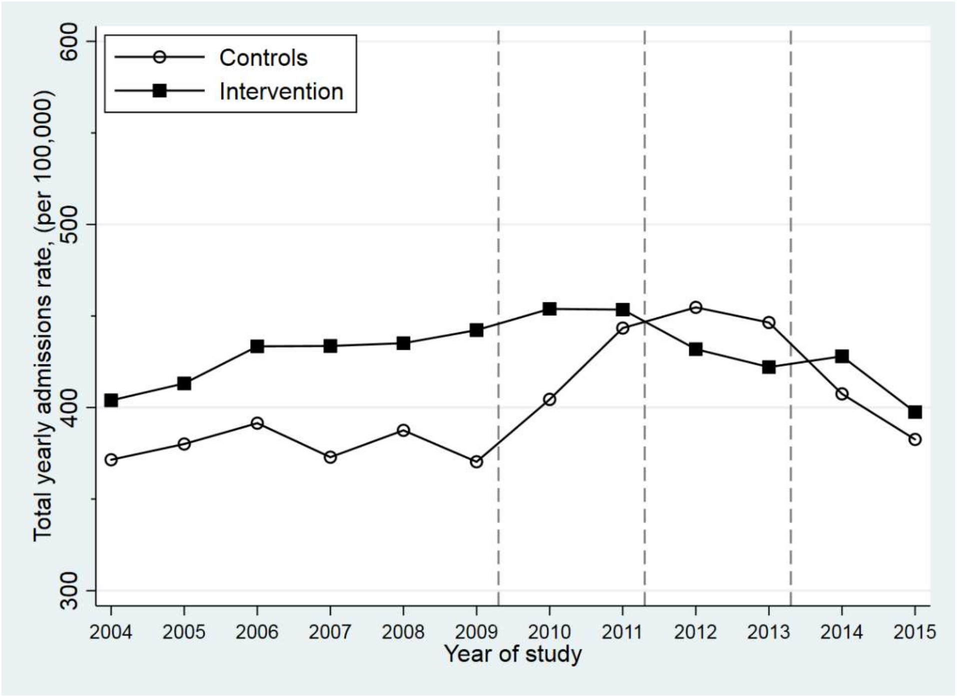

Table S3: Crude annual hospital admission rates for equipment preventable injuries in intervention and control areas from 2004 to 2015 by thirds of equipment density

	**Injury rate per 100,000 population and 95% confidence interval**			
**Year of study**	**Control areas**	**Low equipment density**	**Medium equipment density**	**High equipment Density**
2004	371.51 (358.79 – 384.55)	336.66 (316.02 – 358.29)	412.29 (389.17 – 436.43)	459.68 (436.21 – 484.10)
2005	380.12 (367.33 – 393.23)	367.22 (345.86 – 389.56)	390.35 (368.00 – 413.69)	477.44 (453.74 – 502.06)
2006	391.49 (378.64 – 404.65)	400.85 (378.81 – 423.84)	407.51 (384.92 – 431.09)	487.88 (464.22 – 512.44)
2007	372.89 (360.57 – 385.54)	359.48 (338.98 – 380.89)	421.41 (398.74 – 445.03)	515.47 (491.45 – 540.36)
2008	387.56 (375.20 – 400.22)	386.46 (365.57 – 408.22)	414.36 (392.25 – 437.39)	500.94 (477.62 – 525.10)
2009	370.40 (358.49 – 382.60)	374.27 (354.00 – 395.40)	389.22 (368.06 – 411.27)	556.52 (532.21 – 581.65)
2010	404.48 (392.20 – 417.04)	407.11 (386.17 – 428.89)	432.46 (410.38 – 455.42)	518.50 (495.30 – 542.52)
2011	443.43 (430.72 – 456.42)	409.33 (388.51 – 430.97)	439.87 (417.72 – 462.88)	508.84 (485.97 – 532.51)
2012	454.73 (442.00 – 467.73)	394.06 (373.85 – 415.07)	407.40 (386.32 – 429.33)	491.29 (469.01 – 514.34)
2013	446.47 (433.90 – 459.30)	404.14 (383.74 – 425.34)	404.79 (383.89 – 426.53)	455.68 (434.30 – 477.84)
2014	407.48 (395.51 – 419.71)	400.93 (380.66 – 422.00)	401.38 (380.63 – 422.97)	479.11 (457.22 – 501.78)
2015	382.61 (371.01 – 394.47)	374.38 (354.83 – 394.73)	388.40 (368.03 – 409.62)	428.67 (407.98 – 450.13)

Table S4: Adjusted trends in predicted hospital admission rates for equipment preventable injuries before, during and after the SAH scheme in intervention and control areas by thirds of equipment density

**Time point**	**Predicted deseasonalised hospital admission rate per 100,000 per month and 95% Confidence Interval**				**Difference in Trends[1,3]**
**Baseline period**	**Control**	**Low equipment density**	**Medium equipment density**	**High equipment density**	
January 2004	31.69 (30.56 – 32.88)	29.31 (27.75 – 30.96)	33.78 (32.04 – 35.61)	37.72 (35.95 – 39.57)	
March 2009	31.07 (30.12 – 32.06)	31.89 (30.47 – 33.36)	33.66 (32.18 – 35.19)	44.42 (42.70 – 46.21)	
Change in rate per month (%)[2]	-0.03 (-0.12 – 0.06) P = 0.497	0.14 (0.001 – 0.27) P = 0.051	-0.01 (-0.14 – 0.13) P = 0.931	0.26 (0.14 – 0.38) P < 0.001	0.30 (0.14 – 0.45) P < 0.001
**Implementation period**					
April 2009	31.25 (30.34 – 32.19)	31.98 (30.64 – 33.38)	33.77 (32.36 – 35.23)	44.35 (42.73 – 46.05)	
March 2011	35.78 (34.47 – 37.13)	34.29 (32.49 – 36.18)	36.37 (34.47 – 38.38)	42.90 (40.86 – 45.05)	
Change in rate per month (%)[2]	0.59 (0.37 – 0.81) P < 0.001	0.30 (-0.02 – 0.62) P = 0.066	0.32 (0.003 – 0.64) P = 0.048	-0.14 (-0.43 – 0.14) P = 0.325	-0.73 (-1.10 – -0.37) P <0.001
**1st post intervention period**					
April 2011	35.88 (34.64 – 37.17)	34.25 (32.54 – 36.04)	36.26 (34.46 – 38.15)	42.74 (40.80 – 44.76)	
March 2013	38.46 (37.13 – 39.84)	33.30 (31.61 – 35.07)	33.68 (31.96 – 35.50)	39.06 (37.22 – 41.00)	
Change in rate per month (%)[2]	0.30 (0.06 – 0.54) P = 0.013	-0.12 (-0.47 – 0.23) P = 0.492	-0.32 (-0.67 – 0.03) P = 0.073	-0.39 (-0.71 – -0.07) P = 0.017	-0.69 (-1.09 – -0.29) P = 0.001
**2nd post intervention period**					
April 2013	38.18 (36.91 – 39.49)	33.23 (31.62 – 34.92)	33.63 (31.98 – 35.36)	38.97 (37.21 – 40.82)	
December 2015	30.11 (28.76 – 31.51)	31.27 (29.27 – 33.40)	32.07 (30.01 – 34.28)	36.23 (34.06 – 38.54)	
Change in rate per month (%)[2]	-0.74 (-0.95 – -0.54) P < 0.001	-0.19 (-0.49 – 0.11) P = 0.211	-0.15 (-0.45 – 0.15) P = 0.335	-0.23 (-0.51 – 0.05) P = 0.109	0.51 (0.17 – 0.86) P = 0.004

[1] Between the high density group and the control group.
[2] The change in rate per month is the incidence rate ratio (IRR) and reflects the percentage change in the injury rate per month.
[3] The difference in trends is the difference between the change in rate in intervention areas and the change in rates in the control areas.
