# Supplementary material for: Impact of the national home safety equipment scheme ‘Safe At Home’ on hospital admissions for unintentional injury in children under 5: a controlled interrupted time series analysis
Source: J Epidemiol Community Health. 2021 Jun 22;76(1):53–9. doi: 10.1136/jech-2021-216613 (PMC8666806; doi:10.1136/jech-2021-216613)
Supplement: Supplementary data [file jech-2021-216613supp002.pdf]

code	description	Included in all injury analysis	Included in equipment preventable analysis
S00	Superficial injury of head	Yes	Yes
S000	Superficial injury of scalp	Yes	Yes
S001	Contusion of eyelid and periocular area	Yes	Yes
S002	Other superficial injuries of eyelid and periocular area	Yes	Yes
S003	Superficial injury of nose	Yes	Yes
S004	Superficial injury of ear	Yes	Yes
S005	Superficial injury of lip and oral cavity	Yes	Yes
S007	Multiple superficial injuries of head	Yes	Yes
S008	Superficial injury of other parts of head	Yes	Yes
S009	Superficial injury of head, part unspecified	Yes	Yes
S01	Open wound of head	Yes	Yes
S010	Open wound of scalp	Yes	Yes
S011	Open wound of eyelid and periocular area	Yes	Yes
S012	Open wound of nose	Yes	Yes
S013	Open wound of ear	Yes	Yes
S014	Open wound of cheek and temporomandibular area	Yes	Yes
S015	Open wound of lip and oral cavity	Yes	Yes
S017	Multiple open wounds of head	Yes	Yes
S018	Open wound of other parts of head	Yes	Yes
S019	Open wound of head, part unspecified	Yes	Yes
S02	Fracture of skull and facial bones	Yes	Yes
S020	Fracture of vault of skull	Yes	Yes
S021	Fracture of base of skull	Yes	Yes
S022	Fracture of nasal bones	Yes	Yes
S023	Fracture of orbital floor	Yes	Yes
S024	Fracture of malar and maxillary bones	Yes	Yes
S025	Fracture of tooth	Yes	Yes
S026	Fracture of mandible	Yes	Yes
S027	Multiple fractures involving skull and facial bones	Yes	Yes
S028	Fractures of other skull and facial bones	Yes	Yes
S029	Fracture of skull and facial bones, part unspecified	Yes	Yes
S03	Dislocation, sprain and strain of joints and ligaments of head	Yes	Yes
S030	Dislocation of jaw	Yes	Yes
S031	Dislocation of septal cartilage of nose	Yes	Yes
S032	Dislocation of tooth	Yes	Yes
S033	Dislocation of other and unspecified parts of head	Yes	Yes
S034	Sprain and strain of jaw	Yes	Yes
S035	Sprain and strain of joints and ligaments of other and unspecifi	Yes	Yes
S04	Injury of cranial nerves	Yes	Yes
S040	Injury of optic nerve and pathways	Yes	Yes
S041	Injury of oculomotor nerve	Yes	Yes
S042	Injury of trochlear nerve	Yes	Yes
S043	Injury of trigeminal nerve	Yes	Yes
S044	Injury of abducent nerve	Yes	Yes
S045	Injury of facial nerve	Yes	Yes
S046	Injury of acoustic nerve	Yes	Yes
S047	Injury of accessory nerve	Yes	Yes
S048	Injury of other cranial nerves	Yes	Yes
S049	Injury of unspecified cranial nerve	Yes	Yes
S05	Injury of eye and orbit	Yes	Yes

S050	Injury of conjunctiva and corneal abrasion without mention of f	Yes	Yes
S051	Contusion of eyeball and orbital tissues	Yes	Yes
S052	Ocular laceration and rupture with prolapse or loss of intraocul	Yes	Yes
S053	Ocular laceration without prolapse or loss of intraocular tissue	Yes	Yes
S054	Penetrating wound of orbit with or without foreign body	Yes	Yes
S055	Penetrating wound of eyeball with foreign body	Yes	Yes
S056	Penetrating wound of eyeball without foreign body	Yes	Yes
S057	Avulsion of eye	Yes	Yes
S058	Other injuries of eye and orbit	Yes	Yes
S059	Injury of eye and orbit, unspecified	Yes	Yes
S06	Intracranial injury	Yes	Yes
S060	Concussion	Yes	Yes
S061	Traumatic cerebral oedema	Yes	Yes
S062	Diffuse brain injury	Yes	Yes
S063	Focal brain injury	Yes	Yes
S064	Epidural haemorrhage	Yes	Yes
S065	Traumatic subdural haemorrhage	Yes	Yes
S066	Traumatic subarachnoid haemorrhage	Yes	Yes
S067	Intracranial injury with prolonged coma	Yes	Yes
S068	Other intracranial injuries	Yes	Yes
S069	Intracranial injury, unspecified	Yes	Yes
S07	Crushing injury of head	Yes	Yes
S070	Crushing injury of face	Yes	Yes
S071	Crushing injury of skull	Yes	Yes
S078	Crushing injury of other parts of head	Yes	Yes
S079	Crushing injury of head, part unspecified	Yes	Yes
S08	Traumatic amputation of part of head	Yes	Yes
S080	Avulsion of scalp	Yes	Yes
S081	Traumatic amputation of ear	Yes	Yes
S088	Traumatic amputation of other parts of head	Yes	Yes
S089	Traumatic amputation of unspecified part of head	Yes	Yes
S09	Other and unspecified injuries of head	Yes	Yes
S090	Injury of blood vessels of head, not elsewhere classified	Yes	Yes
S091	Injury of muscle and tendon of head	Yes	Yes
S092	Traumatic rupture of ear drum	Yes	Yes
S097	Multiple injuries of head	Yes	Yes
S098	Other specified injuries of head	Yes	Yes
S099	Unspecified injury of head	Yes	Yes
S10	Superficial injury of neck	Yes	Yes
S100	Contusion of throat	Yes	Yes
S101	Other and unspecified superficial injuries of throat	Yes	Yes
S107	Multiple superficial injuries of neck	Yes	Yes
S108	Superficial injury of other parts of neck	Yes	Yes
S109	Superficial injury of neck, part unspecified	Yes	Yes
S11	Open wound of neck	Yes	Yes
S110	Open wound involving larynx and trachea	Yes	Yes
S111	Open wound involving thyroid gland	Yes	Yes
S112	Open wound involving pharynx and cervical oesophagus	Yes	Yes
S117	Multiple open wounds of neck	Yes	Yes
S118	Open wound of other parts of neck	Yes	Yes
S119	Open wound of neck, part unspecified	Yes	Yes
S12	Fracture of neck	Yes	Yes
S120	Fracture of first cervical vertebra	Yes	Yes

S121	Fracture of second cervical vertebra	Yes	Yes
S122	Fracture of other specified cervical vertebra	Yes	Yes
S127	Multiple fractures of cervical spine	Yes	Yes
S128	Fracture of other parts of neck	Yes	Yes
S129	Fracture of neck, part unspecified	Yes	Yes
S13	Dislocation, sprain and strain of joints and ligaments at neck lev	Yes	Yes
S130	Traumatic rupture of cervical intervertebral disc	Yes	Yes
S131	Dislocation of cervical vertebra	Yes	Yes
S132	Dislocation of other and unspecified parts of neck	Yes	Yes
S133	Multiple dislocations of neck	Yes	Yes
S134	Sprain and strain of cervical spine	Yes	Yes
S135	Sprain and strain of thyroid region	Yes	Yes
S136	Sprain and strain of joints and ligaments of other and unspecifi	Yes	Yes
S14	Injury of nerves and spinal cord at neck level	Yes	Yes
S140	Concussion and oedema of cervical spinal cord	Yes	Yes
S141	Other and unspecified injuries of cervical spinal cord	Yes	Yes
S142	Injury of nerve root of cervical spine	Yes	Yes
S144	Injury of peripheral nerves of neck	Yes	Yes
S145	Injury of cervical sympathetic nerves	Yes	Yes
S146	Injury of other and unspecified nerves of neck	Yes	Yes
S15	Injury of blood vessels at neck level	Yes	Yes
S150	Injury of carotid artery	Yes	Yes
S151	Injury of vertebral artery	Yes	Yes
S152	Injury of external jugular vein	Yes	Yes
S153	Injury of internal jugular vein	Yes	Yes
S157	Injury of multiple blood vessels at neck level	Yes	Yes
S158	Injury of other blood vessels at neck level	Yes	Yes
S159	Injury of unspecified blood vessel at neck level	Yes	Yes
S16X	Injury of muscle and tendon at neck level	Yes	Yes
S17	Crushing injury of neck	Yes	Yes
S170	Crushing injury of larynx and trachea	Yes	Yes
S178	Crushing injury of other parts of neck	Yes	Yes
S179	Crushing injury of neck, part unspecified	Yes	Yes
S18X	Traumatic amputation at neck level	Yes	Yes
S19	Other and unspecified injuries of neck	Yes	Yes
S197	Multiple injuries of neck	Yes	Yes
S198	Other specified injuries of neck	Yes	Yes
S199	Unspecified injury of neck	Yes	Yes
S20	Superficial injury of thorax	Yes	Yes
S200	Contusion of breast	Yes	Yes
S201	Other and unspecified superficial injuries of breast	Yes	Yes
S202	Contusion of thorax	Yes	Yes
S203	Other superficial injuries of front wall of thorax	Yes	Yes
S204	Other superficial injuries of back wall of thorax	Yes	Yes
S207	Multiple superficial injuries of thorax	Yes	Yes
S208	Superficial injury of other and unspecified parts of thorax	Yes	Yes
S21	Open wound of thorax	Yes	Yes
S210	Open wound of breast	Yes	Yes
S211	Open wound of front wall of thorax	Yes	Yes
S212	Open wound of back wall of thorax	Yes	Yes
S217	Multiple open wounds of thoracic wall	Yes	Yes
S218	Open wound of other parts of thorax	Yes	Yes
S219	Open wound of thorax, part unspecified	Yes	Yes

S22	Fracture of rib(s), sternum and thoracic spine	Yes	Yes
S220	Fracture of thoracic vertebra	Yes	Yes
S221	Multiple fractures of thoracic spine	Yes	Yes
S222	Fracture of sternum	Yes	Yes
S223	Fracture of rib	Yes	Yes
S224	Multiple fractures of ribs	Yes	Yes
S225	Flail chest	Yes	Yes
S228	Fracture of other parts of bony thorax	Yes	Yes
S229	Fracture of bony thorax, part unspecified	Yes	Yes
S23	Dislocation, sprain and strain of joints and ligaments of thorax	Yes	Yes
S230	Traumatic rupture of thoracic intervertebral disc	Yes	Yes
S231	Dislocation of thoracic vertebra	Yes	Yes
S232	Dislocation of other and unspecified parts of thorax	Yes	Yes
S233	Sprain and strain of thoracic spine	Yes	Yes
S234	Sprain and strain of ribs and sternum	Yes	Yes
S235	Sprain and strain of other and unspecified parts of thorax	Yes	Yes
S24	Injury of nerves and spinal cord at thorax level	Yes	Yes
S240	Concussion and oedema of thoracic spinal cord	Yes	Yes
S241	Other and unspecified injuries of thoracic spinal cord	Yes	Yes
S242	Injury of nerve root of thoracic spine	Yes	Yes
S243	Injury of peripheral nerves of thorax	Yes	Yes
S244	Injury of thoracic sympathetic nerves	Yes	Yes
S245	Injury of other nerves of thorax	Yes	Yes
S246	Injury of unspecified nerve of thorax	Yes	Yes
S25	Injury of blood vessels of thorax	Yes	Yes
S250	Injury of thoracic aorta	Yes	Yes
S251	Injury of innominate or subclavian artery	Yes	Yes
S252	Injury of superior vena cava	Yes	Yes
S253	Injury of innominate or subclavian vein	Yes	Yes
S254	Injury of pulmonary blood vessels	Yes	Yes
S255	Injury of intercostal blood vessels	Yes	Yes
S257	Injury of multiple blood vessels of thorax	Yes	Yes
S258	Injury of other blood vessels of thorax	Yes	Yes
S259	Injury of unspecified blood vessel of thorax	Yes	Yes
S26	Injury of heart	Yes	Yes
S260	Injury of heart with haemopericardium	Yes	Yes
S268	Other injuries of heart	Yes	Yes
S269	Injury of heart, unspecified	Yes	Yes
S27	Injury of other and unspecified intrathoracic organs	Yes	Yes
S270	Traumatic pneumothorax	Yes	Yes
S271	Traumatic haemothorax	Yes	Yes
S272	Traumatic haemopneumothorax	Yes	Yes
S273	Other injuries of lung	Yes	Yes
S274	Injury of bronchus	Yes	Yes
S275	Injury of thoracic trachea	Yes	Yes
S276	Injury of pleura	Yes	Yes
S277	Multiple injuries of intrathoracic organs	Yes	Yes
S278	Injury of other specified intrathoracic organs	Yes	Yes
S279	Injury of unspecified intrathoracic organ	Yes	Yes
S28	Crushing injury of thorax and traumatic amputation of part of t	Yes	Yes
S280	Crushed chest	Yes	Yes
S281	Traumatic amputation of part of thorax	Yes	Yes
S29	Other and unspecified injuries of thorax	Yes	Yes

S290	Injury of muscle and tendon at thorax level	Yes	Yes
S297	Multiple injuries of thorax	Yes	Yes
S298	Other specified injuries of thorax	Yes	Yes
S299	Unspecified injury of thorax	Yes	Yes
S30	Superficial injury of abdomen, lower back and pelvis	Yes	Yes
S300	Contusion of lower back and pelvis	Yes	Yes
S301	Contusion of abdominal wall	Yes	Yes
S302	Contusion of external genital organs	Yes	Yes
S307	Multiple superficial injuries of abdomen, lower back and pelvis	Yes	Yes
S308	Other superficial injuries of abdomen, lower back and pelvis	Yes	Yes
S309	Superficial injury of abdomen, lower back and pelvis, part unsp	Yes	Yes
S31	Open wound of abdomen, lower back and pelvis	Yes	Yes
S310	Open wound of lower back and pelvis	Yes	Yes
S311	Open wound of abdominal wall	Yes	Yes
S312	Open wound of penis	Yes	Yes
S313	Open wound of scrotum and testes	Yes	Yes
S314	Open wound of vagina and vulva	Yes	Yes
S315	Open wound of other and unspecified external genital organs	Yes	Yes
S317	Multiple open wounds of abdomen, lower back and pelvis	Yes	Yes
S318	Open wound of other and unspecified parts of abdomen	Yes	Yes
S32	Fracture of lumbar spine and pelvis	Yes	Yes
S320	Fracture of lumbar vertebra	Yes	Yes
S321	Fracture of sacrum	Yes	Yes
S322	Fracture of coccyx	Yes	Yes
S323	Fracture of ilium	Yes	Yes
S324	Fracture of acetabulum	Yes	Yes
S325	Fracture of pubis	Yes	Yes
S327	Multiple fractures of lumbar spine and pelvis	Yes	Yes
S328	Fracture of other and unspecified parts of lumbar spine and pe	Yes	Yes
S33	Dislocation, sprain and strain of joints and ligaments of lumbar	Yes	Yes
S330	Traumatic rupture of lumbar intervertebral disc	Yes	Yes
S331	Dislocation of lumbar vertebra	Yes	Yes
S332	Dislocation of sacroiliac and sacrococcygeal joint	Yes	Yes
S333	Dislocation of other and unspecified parts of lumbar spine and	Yes	Yes
S334	Traumatic rupture of symphysis pubis	Yes	Yes
S335	Sprain and strain of lumbar spine	Yes	Yes
S336	Sprain and strain of sacroiliac joint	Yes	Yes
S337	Sprain and strain of other and unspecified parts of lumbar spin	Yes	Yes
S34	Injury of nerves and lumbar spinal cord at abdomen, lower bac	Yes	Yes
S340	Concussion and oedema of lumbar spinal cord	Yes	Yes
S341	Other injury of lumbar spinal cord	Yes	Yes
S342	Injury of nerve root of lumbar and sacral spine	Yes	Yes
S343	Injury of cauda equina	Yes	Yes
S344	Injury of lumbosacral plexus	Yes	Yes
S345	Injury of lumbar, sacral and pelvic sympathetic nerves	Yes	Yes
S346	Injury of peripheral nerve(s) of abdomen, lower back and pelvis	Yes	Yes
S348	Injury of other and unspecified nerves at abdomen, lower back	Yes	Yes
S35	Injury of blood vessels at abdomen, lower back and pelvis level	Yes	Yes
S350	Injury of abdominal aorta	Yes	Yes
S351	Injury of inferior vena cava	Yes	Yes
S352	Injury of coeliac or mesenteric artery	Yes	Yes
S353	Injury of portal or splenic vein	Yes	Yes
S354	Injury of renal blood vessels	Yes	Yes

S355	Injury of iliac blood vessels	Yes	Yes
S357	Injury of multiple blood vessels at abdomen, lower back and pe	Yes	Yes
S358	Injury of other blood vessels at abdomen, lower back and pelvi	Yes	Yes
S359	Injury of unspecified blood vessel at abdomen, lower back and	Yes	Yes
S36	Injury of intra-abdominal organs	Yes	Yes
S360	Injury of spleen	Yes	Yes
S361	Injury of liver or gallbladder	Yes	Yes
S362	Injury of pancreas	Yes	Yes
S363	Injury of stomach	Yes	Yes
S364	Injury of small intestine	Yes	Yes
S365	Injury of colon	Yes	Yes
S366	Injury of rectum	Yes	Yes
S367	Injury of multiple intra-abdominal organs	Yes	Yes
S368	Injury of other intra-abdominal organs	Yes	Yes
S369	Injury of unspecified intra-abdominal organ	Yes	Yes
S37	Injury of urinary and pelvic organs	Yes	Yes
S370	Injury of kidney	Yes	Yes
S371	Injury of ureter	Yes	Yes
S372	Injury of bladder	Yes	Yes
S373	Injury of urethra	Yes	Yes
S374	Injury of ovary	Yes	Yes
S375	Injury of fallopian tube	Yes	Yes
S376	Injury of uterus	Yes	Yes
S377	Injury of multiple pelvic organs	Yes	Yes
S378	Injury of other pelvic organs	Yes	Yes
S379	Injury of unspecified pelvic organ	Yes	Yes
S38	Crushing injury and traumatic amputation of part of abdomen,	Yes	Yes
S380	Crushing injury of external genital organs	Yes	Yes
S381	Crushing injury of other and unspecified parts of abdomen, low	Yes	Yes
S382	Traumatic amputation of external genital organs	Yes	Yes
S383	Traumatic amputation of other and unspecified parts of abdom	Yes	Yes
S39	Other and unspecified injuries of abdomen, lower back and pel	Yes	Yes
S390	Injury of muscle and tendon of abdomen, lower back and pelvis	Yes	Yes
S396	Injury of intra-abdominal organ(s) with pelvic organ(s)	Yes	Yes
S397	Other multiple injuries of abdomen, lower back and pelvis	Yes	Yes
S398	Other specified injuries of abdomen, lower back and pelvis	Yes	Yes
S399	Unspecified injury of abdomen, lower back and pelvis	Yes	Yes
S40	Superficial injury of shoulder and upper arm	Yes	Yes
S400	Contusion of shoulder and upper arm	Yes	Yes
S407	Multiple superficial injuries of shoulder and upper arm	Yes	Yes
S408	Other superficial injuries of shoulder and upper arm	Yes	Yes
S409	Superficial injury of shoulder and upper arm, unspecified	Yes	Yes
S41	Open wound of shoulder and upper arm	Yes	Yes
S410	Open wound of shoulder	Yes	Yes
S411	Open wound of upper arm	Yes	Yes
S417	Multiple open wounds of shoulder and upper arm	Yes	Yes
S418	Open wound of other and unspecified parts of shoulder girdle	Yes	Yes
S42	Fracture of shoulder and upper arm	Yes	Yes
S420	Fracture of clavicle	Yes	Yes
S421	Fracture of scapula	Yes	Yes
S422	Fracture of upper end of humerus	Yes	Yes
S423	Fracture of shaft of humerus	Yes	Yes
S424	Fracture of lower end of humerus	Yes	Yes

S427	Multiple fractures of clavicle, scapula and humerus	Yes	Yes
S428	Fracture of other parts of shoulder and upper arm	Yes	Yes
S429	Fracture of shoulder girdle, part unspecified	Yes	Yes
S43	Dislocation, sprain and strain of joints and ligaments of shoulde	Yes	Yes
S430	Dislocation of shoulder joint	Yes	Yes
S431	Dislocation of acromioclavicular joint	Yes	Yes
S432	Dislocation of sternoclavicular joint	Yes	Yes
S433	Dislocation of other and unspecified parts of shoulder girdle	Yes	Yes
S434	Sprain and strain of shoulder joint	Yes	Yes
S435	Sprain and strain of acromioclavicular joint	Yes	Yes
S436	Sprain and strain of sternoclavicular joint	Yes	Yes
S437	Sprain and strain of other and unspecified parts of shoulder gir	Yes	Yes
S44	Injury of nerves at shoulder and upper arm level	Yes	Yes
S440	Injury of ulnar nerve at upper arm level	Yes	Yes
S441	Injury of median nerve at upper arm level	Yes	Yes
S442	Injury of radial nerve at upper arm level	Yes	Yes
S443	Injury of axillary nerve	Yes	Yes
S444	Injury of musculocutaneous nerve	Yes	Yes
S445	Injury of cutaneous sensory nerve at shoulder and upper arm le	Yes	Yes
S447	Injury of multiple nerves at shoulder and upper arm level	Yes	Yes
S448	Injury of other nerves at shoulder and upper arm level	Yes	Yes
S449	Injury of unspecified nerve at shoulder and upper arm level	Yes	Yes
S45	Injury of blood vessels at shoulder and upper arm level	Yes	Yes
S450	Injury of axillary artery	Yes	Yes
S451	Injury of brachial artery	Yes	Yes
S452	Injury of axillary or brachial vein	Yes	Yes
S453	Injury of superficial vein at shoulder and upper arm level	Yes	Yes
S457	Injury of multiple blood vessels at shoulder and upper arm leve	Yes	Yes
S458	Injury of other blood vessels at shoulder and upper arm level	Yes	Yes
S459	Injury of unspecified blood vessel at shoulder and upper arm le	Yes	Yes
S46	Injury of muscle and tendon at shoulder and upper arm level	Yes	Yes
S460	Injury of muscle(s) and tendon(s) of the rotator cuff of shoulde	Yes	Yes
S461	Injury of muscle and tendon of long head of biceps	Yes	Yes
S462	Injury of muscle and tendon of other parts of biceps	Yes	Yes
S463	Injury of muscle and tendon of triceps	Yes	Yes
S467	Injury of multiple muscles and tendons at shoulder and upper a	Yes	Yes
S468	Injury of other muscles and tendons at shoulder and upper arm	Yes	Yes
S469	Injury of unspecified muscle and tendon at shoulder and upper	Yes	Yes
S47X	Crushing injury of shoulder and upper arm	Yes	Yes
S48	Traumatic amputation of shoulder and upper arm	Yes	Yes
S480	Traumatic amputation at shoulder joint	Yes	Yes
S481	Traumatic amputation at level between shoulder and elbow	Yes	Yes
S489	Traumatic amputation of shoulder and upper arm, level unspec	Yes	Yes
S49	Other and unspecified injuries of shoulder and upper arm	Yes	Yes
S497	Multiple injuries of shoulder and upper arm	Yes	Yes
S498	Other specified injuries of shoulder and upper arm	Yes	Yes
S499	Unspecified injury of shoulder and upper arm	Yes	Yes
S50	Superficial injury of forearm	Yes	Yes
S500	Contusion of elbow	Yes	Yes
S501	Contusion of other and unspecified parts of forearm	Yes	Yes
S507	Multiple superficial injuries of forearm	Yes	Yes
S508	Other superficial injuries of forearm	Yes	Yes
S509	Superficial injury of forearm, unspecified	Yes	Yes

S51	Open wound of forearm	Yes	Yes
S510	Open wound of elbow	Yes	Yes
S517	Multiple open wounds of forearm	Yes	Yes
S518	Open wound of other parts of forearm	Yes	Yes
S519	Open wound of forearm, part unspecified	Yes	Yes
S52	Fracture of forearm	Yes	Yes
S520	Fracture of upper end of ulna	Yes	Yes
S521	Fracture of upper end of radius	Yes	Yes
S522	Fracture of shaft of ulna	Yes	Yes
S523	Fracture of shaft of radius	Yes	Yes
S524	Fracture of shafts of both ulna and radius	Yes	Yes
S525	Fracture of lower end of radius	Yes	Yes
S526	Fracture of lower end of both ulna and radius	Yes	Yes
S527	Multiple fractures of forearm	Yes	Yes
S528	Fracture of other parts of forearm	Yes	Yes
S529	Fracture of forearm, part unspecified	Yes	Yes
S53	Dislocation, sprain and strain of joints and ligaments of elbow	Yes	Yes
S530	Dislocation of radial head	Yes	Yes
S531	Dislocation of elbow, unspecified	Yes	Yes
S532	Traumatic rupture of radial collateral ligament	Yes	Yes
S533	Traumatic rupture of ulnar collateral ligament	Yes	Yes
S534	Sprain and strain of elbow	Yes	Yes
S54	Injury of nerves at forearm level	Yes	Yes
S540	Injury of ulnar nerve at forearm level	Yes	Yes
S541	Injury of median nerve at forearm level	Yes	Yes
S542	Injury of radial nerve at forearm level	Yes	Yes
S543	Injury of cutaneous sensory nerve at forearm level	Yes	Yes
S547	Injury of multiple nerves at forearm level	Yes	Yes
S548	Injury of other nerves at forearm level	Yes	Yes
S549	Injury of unspecified nerve at forearm level	Yes	Yes
S55	Injury of blood vessels at forearm level	Yes	Yes
S550	Injury of ulnar artery at forearm level	Yes	Yes
S551	Injury of radial artery at forearm level	Yes	Yes
S552	Injury of vein at forearm level	Yes	Yes
S557	Injury of multiple blood vessels at forearm level	Yes	Yes
S558	Injury of other blood vessels at forearm level	Yes	Yes
S559	Injury of unspecified blood vessel at forearm level	Yes	Yes
S56	Injury of muscle and tendon at forearm level	Yes	Yes
S560	Injury of flexor muscle and tendon of thumb at forearm level	Yes	Yes
S561	Injury of long flexor muscle and tendon of other finger(s) at for	Yes	Yes
S562	Injury of other flexor muscle and tendon at forearm level	Yes	Yes
S563	Injury of extensor or abductor muscles and tendons of thumb a	Yes	Yes
S564	Injury of extensor muscle and tendon of other finger(s) at forea	Yes	Yes
S565	Injury of other extensor muscle and tendon at forearm level	Yes	Yes
S567	Injury of multiple muscles and tendons at forearm level	Yes	Yes
S568	Injury of other and unspecified muscles and tendons at forearn	Yes	Yes
S57	Crushing injury of forearm	Yes	Yes
S570	Crushing injury of elbow	Yes	Yes
S578	Crushing injury of other parts of forearm	Yes	Yes
S579	Crushing injury of forearm, part unspecified	Yes	Yes
S58	Traumatic amputation of forearm	Yes	Yes
S580	Traumatic amputation at elbow level	Yes	Yes
S581	Traumatic amputation at level between elbow and wrist	Yes	Yes

S589	Traumatic amputation of forearm, level unspecified	Yes	Yes
S59	Other and unspecified injuries of forearm	Yes	Yes
S597	Multiple injuries of forearm	Yes	Yes
S598	Other specified injuries of forearm	Yes	Yes
S599	Unspecified injury of forearm	Yes	Yes
S60	Superficial injury of wrist and hand	Yes	Yes
S600	Contusion of finger(s) without damage to nail	Yes	Yes
S601	Contusion of finger(s) with damage to nail	Yes	Yes
S602	Contusion of other parts of wrist and hand	Yes	Yes
S607	Multiple superficial injuries of wrist and hand	Yes	Yes
S608	Other superficial injuries of wrist and hand	Yes	Yes
S609	Superficial injury of wrist and hand, unspecified	Yes	Yes
S61	Open wound of wrist and hand	Yes	Yes
S610	Open wound of finger(s) without damage to nail	Yes	Yes
S611	Open wound of finger(s) with damage to nail	Yes	Yes
S617	Multiple open wounds of wrist and hand	Yes	Yes
S618	Open wound of other parts of wrist and hand	Yes	Yes
S619	Open wound of wrist and hand part, part unspecified	Yes	Yes
S62	Fracture at wrist and hand level	Yes	Yes
S620	Fracture of navicular [scaphoid] bone of hand	Yes	Yes
S621	Fracture of other carpal bone(s)	Yes	Yes
S622	Fracture of first metacarpal bone	Yes	Yes
S623	Fracture of other metacarpal bone	Yes	Yes
S624	Multiple fractures of metacarpal bones	Yes	Yes
S625	Fracture of thumb	Yes	Yes
S626	Fracture of other finger	Yes	Yes
S627	Multiple fractures of fingers	Yes	Yes
S628	Fracture of other and unspecified parts of wrist and hand	Yes	Yes
S63	Dislocation, sprain and strain of joints and ligaments at wrist ar	Yes	Yes
S630	Dislocation of wrist	Yes	Yes
S631	Dislocation of finger	Yes	Yes
S632	Multiple dislocations of fingers	Yes	Yes
S633	Traumatic rupture of ligament of wrist and carpus	Yes	Yes
S634	Traumatic rupture of ligament of finger at metacarpophalange	Yes	Yes
S635	Sprain and strain of wrist	Yes	Yes
S636	Sprain and strain of finger(s)	Yes	Yes
S637	Sprain and strain of other and unspecified parts of hand	Yes	Yes
S64	Injury of nerves at wrist and hand level	Yes	Yes
S640	Injury of ulnar nerve at wrist and hand level	Yes	Yes
S641	Injury of median nerve at wrist and hand level	Yes	Yes
S642	Injury of radial nerve at wrist and hand level	Yes	Yes
S643	Injury of digital nerve of thumb	Yes	Yes
S644	Injury of digital nerve of other finger	Yes	Yes
S647	Injury of multiple nerves at wrist and hand level	Yes	Yes
S648	Injury of other nerves at wrist and hand level	Yes	Yes
S649	Injury of unspecified nerve at wrist and hand level	Yes	Yes
S65	Injury of blood vessels at wrist and hand level	Yes	Yes
S650	Injury of ulnar artery at wrist and hand level	Yes	Yes
S651	Injury of radial artery at wrist and hand level	Yes	Yes
S652	Injury of superficial palmar arch	Yes	Yes
S653	Injury of deep palmar arch	Yes	Yes
S654	Injury of blood vessel(s) of thumb	Yes	Yes
S655	Injury of blood vessel(s) of other finger	Yes	Yes

S657	Injury of multiple blood vessels at wrist and hand level	Yes	Yes
S658	Injury of other blood vessels at wrist and hand level	Yes	Yes
S659	Injury of unspecified blood vessel at wrist and hand level	Yes	Yes
S66	Injury of muscle and tendon at wrist and hand level	Yes	Yes
S660	Injury of long flexor muscle and tendon of thumb at wrist and h	Yes	Yes
S661	Injury of flexor muscle and tendon of other finger at wrist and h	Yes	Yes
S662	Injury of extensor muscle and tendon of thumb at wrist and ha	Yes	Yes
S663	Injury of extensor muscle and tendon of other finger at wrist ar	Yes	Yes
S664	Injury of intrinsic muscle and tendon of thumb at wrist and han	Yes	Yes
S665	Injury of intrinsic muscle and tendon of other finger at wrist an	Yes	Yes
S666	Injury of multiple flexor muscles and tendons at wrist and hand	Yes	Yes
S667	Injury of multiple extensor muscles and tendons at wrist and ha	Yes	Yes
S668	Injury of other muscles and tendons at wrist and hand level	Yes	Yes
S669	Injury of unspecified muscle and tendon at wrist and hand leve	Yes	Yes
S67	Crushing injury of wrist and hand	Yes	Yes
S670	Crushing injury of thumb and other finger(s)	Yes	Yes
S678	Crushing injury of other and unspecified parts of wrist and hand	Yes	Yes
S68	Traumatic amputation of wrist and hand	Yes	Yes
S680	Traumatic amputation of thumb (complete)(partial)	Yes	Yes
S681	Traumatic amputation of other single finger (complete)(partial)	Yes	Yes
S682	Traumatic amputation of two or more fingers alone (complete)	Yes	Yes
S683	Combined traumatic amputation of (part of) finger(s) with othe	Yes	Yes
S684	Traumatic amputation of hand at wrist level	Yes	Yes
S688	Traumatic amputation of other parts of wrist and hand	Yes	Yes
S689	Traumatic amputation of wrist and hand, level unspecified	Yes	Yes
S69	Other and unspecified injuries of wrist and hand	Yes	Yes
S697	Multiple injuries of wrist and hand	Yes	Yes
S698	Other specified injuries of wrist and hand	Yes	Yes
S699	Unspecified injury of wrist and hand	Yes	Yes
S70	Superficial injury of hip and thigh	Yes	Yes
S700	Contusion of hip	Yes	Yes
S701	Contusion of thigh	Yes	Yes
S707	Multiple superficial injuries of hip and thigh	Yes	Yes
S708	Other superficial injuries of hip and thigh	Yes	Yes
S709	Superficial injury of hip and thigh, unspecified	Yes	Yes
S71	Open wound of hip and thigh	Yes	Yes
S710	Open wound of hip	Yes	Yes
S711	Open wound of thigh	Yes	Yes
S717	Multiple open wounds of hip and thigh	Yes	Yes
S718	Open wound of other and unspecified parts of pelvic girdle	Yes	Yes
S72	Fracture of femur	Yes	Yes
S720	Fracture of neck of femur	Yes	Yes
S721	Pertrochanteric fracture	Yes	Yes
S722	Subtrochanteric fracture	Yes	Yes
S723	Fracture of shaft of femur	Yes	Yes
S724	Fracture of lower end of femur	Yes	Yes
S727	Multiple fractures of femur	Yes	Yes
S728	Fractures of other parts of femur	Yes	Yes
S729	Fracture of femur, part unspecified	Yes	Yes
S73	Dislocation, sprain and strain of joint and ligaments of hip	Yes	Yes
S730	Dislocation of hip	Yes	Yes
S731	Sprain and strain of hip	Yes	Yes
S74	Injury of nerves at hip and thigh level	Yes	Yes

S740	Injury of sciatic nerve at hip and thigh level	Yes	Yes
S741	Injury of femoral nerve at hip and thigh level	Yes	Yes
S742	Injury of cutaneous sensory nerve at hip and thigh level	Yes	Yes
S747	Injury of multiple nerves at hip and thigh level	Yes	Yes
S748	Injury of other nerves at hip and thigh level	Yes	Yes
S749	Injury of unspecified nerve at hip and thigh level	Yes	Yes
S75	Injury of blood vessels at hip and thigh level	Yes	Yes
S750	Injury of femoral artery	Yes	Yes
S751	Injury of femoral vein at hip and thigh level	Yes	Yes
S752	Injury of greater saphenous vein at hip and thigh level	Yes	Yes
S757	Injury of multiple blood vessels at hip and thigh level	Yes	Yes
S758	Injury of other blood vessels at hip and thigh level	Yes	Yes
S759	Injury of unspecified blood vessel at hip and thigh level	Yes	Yes
S76	Injury of muscle and tendon at hip and thigh level	Yes	Yes
S760	Injury of muscle and tendon of hip	Yes	Yes
S761	Injury of quadriceps muscle and tendon	Yes	Yes
S762	Injury of adductor muscle and tendon of thigh	Yes	Yes
S763	Injury of muscle and tendon of the posterior muscle group at th	Yes	Yes
S764	Injury of other and unspecified muscles and tendons at thigh le	Yes	Yes
S767	Injury of multiple muscles and tendons at hip and thigh level	Yes	Yes
S77	Crushing injury of hip and thigh	Yes	Yes
S770	Crushing injury of hip	Yes	Yes
S771	Crushing injury of thigh	Yes	Yes
S772	Crushing injury of hip with thigh	Yes	Yes
S78	Traumatic amputation of hip and thigh	Yes	Yes
S780	Traumatic amputation at hip joint	Yes	Yes
S781	Traumatic amputation at level between hip and knee	Yes	Yes
S789	Traumatic amputation of hip and thigh, level unspecified	Yes	Yes
S79	Other and specified injuries of hip and thigh	Yes	Yes
S797	Multiple injuries of hip and thigh	Yes	Yes
S798	Other specified injuries of hip and thigh	Yes	Yes
S799	Unspecified injury of hip and thigh	Yes	Yes
S80	Superficial injury of lower leg	Yes	Yes
S800	Contusion of knee	Yes	Yes
S801	Contusion of other and unspecified parts of lower leg	Yes	Yes
S807	Multiple superficial injuries of lower leg	Yes	Yes
S808	Other superficial injuries of lower leg	Yes	Yes
S809	Superficial injury of lower leg, unspecified	Yes	Yes
S81	Open wound of lower leg	Yes	Yes
S810	Open wound of knee	Yes	Yes
S817	Multiple open wounds of lower leg	Yes	Yes
S818	Open wound of other parts of lower leg	Yes	Yes
S819	Open wound of lower leg, part unspecified	Yes	Yes
S82	Fracture of lower leg, including ankle	Yes	Yes
S820	Fracture of patella	Yes	Yes
S821	Fracture of upper end of tibia	Yes	Yes
S822	Fracture of shaft of tibia	Yes	Yes
S823	Fracture of lower end of tibia	Yes	Yes
S824	Fracture of fibula alone	Yes	Yes
S825	Fracture of medial malleolus	Yes	Yes
S826	Fracture of lateral malleolus	Yes	Yes
S827	Multiple fractures of lower leg	Yes	Yes
S828	Fractures of other parts of lower leg	Yes	Yes

S829	Fracture of lower leg, part unspecified	Yes	Yes
S83	Dislocation, sprain and strain of joints and ligaments of knee	Yes	Yes
S830	Dislocation of patella	Yes	Yes
S831	Dislocation of knee	Yes	Yes
S832	Tear of meniscus, current	Yes	Yes
S833	Tear of articular cartilage of knee, current	Yes	Yes
S834	Sprain and strain involving (fibular)(tibial) collateral ligament o	Yes	Yes
S835	Sprain and strain involving (anterior)(posterior) cruciate ligame	Yes	Yes
S836	Sprain and strain of other and unspecified parts of knee	Yes	Yes
S837	Injury to multiple structures of knee	Yes	Yes
S84	Injury of nerves at lower leg level	Yes	Yes
S840	Injury of tibial nerve at lower leg level	Yes	Yes
S841	Injury of peroneal nerve at lower leg level	Yes	Yes
S842	Injury of cutaneous sensory nerve at lower leg level	Yes	Yes
S847	Injury of multiple nerves at lower leg level	Yes	Yes
S848	Injury of other nerves at lower leg level	Yes	Yes
S849	Injury of unspecified nerve at lower leg level	Yes	Yes
S85	Injury of blood vessels at lower leg level	Yes	Yes
S850	Injury of popliteal artery	Yes	Yes
S851	Injury of (anterior)(posterior) tibial artery	Yes	Yes
S852	Injury of peroneal artery	Yes	Yes
S853	Injury of greater saphenous vein at lower leg level	Yes	Yes
S854	Injury of lesser saphenous vein at lower leg level	Yes	Yes
S855	Injury of popliteal vein	Yes	Yes
S857	Injury of multiple blood vessels at lower leg level	Yes	Yes
S858	Injury of other blood vessels at lower leg level	Yes	Yes
S859	Injury of unspecified blood vessel at lower leg level	Yes	Yes
S86	Injury of muscle and tendon at lower leg level	Yes	Yes
S860	Injury of Achilles tendon	Yes	Yes
S861	Injury of other muscle(s) and tendon(s) of posterior muscle gro	Yes	Yes
S862	Injury of muscle(s) and tendon(s) of anterior muscle group at lo	Yes	Yes
S863	Injury of muscle(s) and tendon(s) of peroneal muscle group at l	Yes	Yes
S867	Injury of multiple muscles and tendons at lower leg level	Yes	Yes
S868	Injury of other muscles and tendons at lower leg level	Yes	Yes
S869	Injury of unspecified muscle and tendon at lower leg level	Yes	Yes
S87	Crushing injury of lower leg	Yes	Yes
S870	Crushing injury of knee	Yes	Yes
S878	Crushing injury of other and unspecified parts of lower leg	Yes	Yes
S88	Traumatic amputation of lower leg	Yes	Yes
S880	Traumatic amputation at knee level	Yes	Yes
S881	Traumatic amputation at level between knee and ankle	Yes	Yes
S889	Traumatic amputation of lower leg, level unspecified	Yes	Yes
S89	Other and unspecified injuries of lower leg	Yes	Yes
S897	Multiple injuries of lower leg	Yes	Yes
S898	Other specified injuries of lower leg	Yes	Yes
S899	Unspecified injury of lower leg	Yes	Yes
S90	Superficial injury of ankle and foot	Yes	Yes
S900	Contusion of ankle	Yes	Yes
S901	Contusion of toe(s) without damage to nail	Yes	Yes
S902	Contusion of toe(s) with damage to nail	Yes	Yes
S903	Contusion of other and unspecified parts of foot	Yes	Yes
S907	Multiple superficial injuries of ankle and foot	Yes	Yes
S908	Other superficial injuries of ankle and foot	Yes	Yes

S909	Superficial injury of ankle and foot, unspecified	Yes	Yes
S91	Open wound of ankle and foot	Yes	Yes
S910	Open wound of ankle	Yes	Yes
S911	Open wound of toe(s) without damage to nail	Yes	Yes
S912	Open wound of toe(s) with damage to nail	Yes	Yes
S913	Open wound of other parts of foot	Yes	Yes
S917	Multiple open wounds of ankle and foot	Yes	Yes
S92	Fracture of foot, except ankle	Yes	Yes
S920	Fracture of calcaneus	Yes	Yes
S921	Fracture of talus	Yes	Yes
S922	Fracture of other tarsal bone(s)	Yes	Yes
S923	Fracture of metatarsal bone	Yes	Yes
S924	Fracture of great toe	Yes	Yes
S925	Fracture of other toe	Yes	Yes
S927	Multiple fractures of foot	Yes	Yes
S929	Fracture of foot, unspecified	Yes	Yes
S93	Dislocation, sprain and strain of joints and ligaments at ankle a	Yes	Yes
S930	Dislocation of ankle joint	Yes	Yes
S931	Dislocation of toe(s)	Yes	Yes
S932	Rupture of ligaments at ankle and foot level	Yes	Yes
S933	Dislocation of other and unspecified parts of foot	Yes	Yes
S934	Sprain and strain of ankle	Yes	Yes
S935	Sprain and strain of toe(s)	Yes	Yes
S936	Sprain and strain of other and unspecified parts of foot	Yes	Yes
S94	Injury of nerves at ankle and foot level	Yes	Yes
S940	Injury of lateral plantar nerve	Yes	Yes
S941	Injury of medial plantar nerve	Yes	Yes
S942	Injury of deep peroneal nerve at ankle and foot level	Yes	Yes
S943	Injury of cutaneous sensory nerve at ankle and foot level	Yes	Yes
S947	Injury of multiple nerves at ankle and foot level	Yes	Yes
S948	Injury of other nerves at ankle and foot level	Yes	Yes
S949	Injury of unspecified nerve at ankle and foot level	Yes	Yes
S95	Injury of blood vessels at ankle and foot level	Yes	Yes
S950	Injury of dorsal artery of foot	Yes	Yes
S951	Injury of plantar artery of foot	Yes	Yes
S952	Injury of dorsal vein of foot	Yes	Yes
S957	Injury of multiple blood vessels at ankle and foot level	Yes	Yes
S958	Injury of other blood vessels at ankle and foot level	Yes	Yes
S959	Injury of unspecified blood vessel at ankle and foot level	Yes	Yes
S96	Injury of muscle and tendon at ankle and foot level	Yes	Yes
S960	Injury of muscle and tendon of long flexor muscle of toe at ankl	Yes	Yes
S961	Injury of muscle and tendon of long extensor muscle of toe at a	Yes	Yes
S962	Injury of intrinsic muscle and tendon at ankle and foot level	Yes	Yes
S967	Injury of multiple muscles and tendons at ankle and foot level	Yes	Yes
S968	Injury of other muscles and tendons at ankle and foot level	Yes	Yes
S969	Injury of unspecified muscle and tendon at ankle and foot level	Yes	Yes
S97	Crushing injury of ankle and foot	Yes	Yes
S970	Crushing injury of ankle	Yes	Yes
S971	Crushing injury of toe(s)	Yes	Yes
S978	Crushing injury of other parts of ankle and foot	Yes	Yes
S98	Traumatic amputation of ankle and foot	Yes	Yes
S980	Traumatic amputation of foot at ankle level	Yes	Yes
S981	Traumatic amputation of one toe	Yes	Yes

S982	Traumatic amputation of two or more toes	Yes	Yes
S983	Traumatic amputation of other parts of foot	Yes	Yes
S984	Traumatic amputation of foot, level unspecified	Yes	Yes
S99	Other and unspecified injuries of ankle and foot	Yes	Yes
S997	Multiple injuries of ankle and foot	Yes	Yes
S998	Other specified injuries of ankle and foot	Yes	Yes
S999	Unspecified injury of ankle and foot	Yes	Yes
T00	Superficial injuries involving multiple body regions	Yes	Yes
T000	Superficial injuries involving head with neck	Yes	Yes
T001	Superficial injuries involving thorax with abdomen, lower back	Yes	Yes
T002	Superficial injuries involving multiple regions of upper limb(s)	Yes	Yes
T003	Superficial injuries involving multiple regions of lower limb(s)	Yes	Yes
T006	Superficial injuries involving multiple regions of upper limb(s) v	Yes	Yes
T008	Superficial injuries involving other combinations of body regior	Yes	Yes
T009	Multiple superficial injuries, unspecified	Yes	Yes
T01	Open wounds involving multiple body regions	Yes	Yes
T010	Open wounds involving head with neck	Yes	Yes
T011	Open wounds involving thorax with abdomen, lower back and	Yes	Yes
T012	Open wounds involving multiple regions of upper limb(s)	Yes	Yes
T013	Open wounds involving multiple regions of lower limb(s)	Yes	Yes
T016	Open wounds involving multiple regions of upper limb(s) with l	Yes	Yes
T018	Open wounds involving other combinations of body regions	Yes	Yes
T019	Multiple open wounds, unspecified	Yes	Yes
T02	Fractures involving multiple body regions	Yes	Yes
T020	Fractures involving head with neck	Yes	Yes
T021	Fractures involving thorax with lower back and pelvis	Yes	Yes
T022	Fractures involving multiple regions of one upper limb	Yes	Yes
T023	Fractures involving multiple regions of one lower limb	Yes	Yes
T024	Fractures involving multiple regions of both upper limbs	Yes	Yes
T025	Fractures involving multiple regions of both lower limbs	Yes	Yes
T026	Fractures involving multiple regions of upper limb(s) with lowe	Yes	Yes
T027	Fractures involving thorax with lower back and pelvis with limb	Yes	Yes
T028	Fractures involving other combinations of body regions	Yes	Yes
T029	Multiple fractures, unspecified	Yes	Yes
T03	Dislocations, sprains and strains involving multiple body region	Yes	Yes
T030	Dislocations, sprains and strains involving head with neck	Yes	Yes
T031	Dislocations, sprains and strains involving thorax with lower ba	Yes	Yes
T032	Dislocations, sprains and strains involving multiple regions of u	Yes	Yes
T033	Dislocations, sprains and strains involving multiple regions of lo	Yes	Yes
T034	Dislocations, sprains and strains involving multiple regions of u	Yes	Yes
T038	Dislocations, sprains and strains involving other combinations c	Yes	Yes
T039	Multiple dislocations, sprains and strains, unspecified	Yes	Yes
T04	Crushing injuries involving multiple body regions	Yes	Yes
T040	Crushing injuries involving head with neck	Yes	Yes
T041	Crushing injuries involving thorax with abdomen, lower back ar	Yes	Yes
T042	Crushing injuries involving multiple regions of upper limb(s)	Yes	Yes
T043	Crushing injuries involving multiple regions of lower limb(s)	Yes	Yes
T044	Crushing injuries involving multiple regions of upper limb(s) wit	Yes	Yes
T047	Crushing injuries of thorax with abdomen, lower back and pelvi	Yes	Yes
T048	Crushing injuries involving other combinations of body regions	Yes	Yes
T049	Multiple crushing injuries, unspecified	Yes	Yes
T05	Traumatic amputations involving multiple body regions	Yes	Yes
T050	Traumatic amputation of both hands	Yes	Yes

T051	Traumatic amputation of one hand and other arm [any level, e›	Yes	Yes
T052	Traumatic amputation of both arms [any level]	Yes	Yes
T053	Traumatic amputation of both feet	Yes	Yes
T054	Traumatic amputation of one foot and other leg [any level, exc	Yes	Yes
T055	Traumatic amputation of both legs [any level]	Yes	Yes
T056	Traumatic amputation of upper and lower limbs, any combinat	Yes	Yes
T058	Traumatic amputations involving other combinations of body r	Yes	Yes
T059	Multiple traumatic amputations, unspecified	Yes	Yes
T06	Other injuries involving multiple body regions, not elsewhere c	Yes	Yes
T060	Injuries of brain and cranial nerves with injuries of nerves and s	Yes	Yes
T061	Injuries of nerves and spinal cord involving other multiple body	Yes	Yes
T062	Injuries of nerves involving multiple body regions	Yes	Yes
T063	Injuries of blood vessels involving multiple body regions	Yes	Yes
T064	Injuries of muscles and tendons involving multiple body region:	Yes	Yes
T065	Injuries of intrathoracic organs with intra-abdominal and pelvic	Yes	Yes
T068	Other specified injuries involving multiple body regions	Yes	Yes
T07X	Unspecified multiple injuries	Yes	Yes
T08X	Fracture of spine, level unspecified	Yes	Yes
T080	Fracture of spine, level unspecified	Yes	Yes
T09	Other injuries of spine and trunk, level unspecified	Yes	Yes
T090	Superficial injury of trunk, level unspecified	Yes	Yes
T091	Open wound of trunk, level unspecified	Yes	Yes
T092	Dislocation, sprain and strain of unspecified joint and ligament	Yes	Yes
T093	Injury of spinal cord, level unspecified	Yes	Yes
T094	Injury of unspecified nerve, spinal nerve root and plexus of trur	Yes	Yes
T095	Injury of unspecified muscle and tendon of trunk	Yes	Yes
T096	Traumatic amputation of trunk, level unspecified	Yes	Yes
T098	Other specified injuries of trunk, level unspecified	Yes	Yes
T099	Unspecified injury of trunk, level unspecified	Yes	Yes
T10X	Fracture of upper limb, level unspecified	Yes	Yes
T100	Fracture of upper limb, level unspecified	Yes	Yes
T11	Other injuries of upper limb, level unspecified	Yes	Yes
T110	Superficial injury of upper limb, level unspecified	Yes	Yes
T111	Open wound of upper limb, level unspecified	Yes	Yes
T112	Dislocation, sprain and strain of unspecified joint and ligament	Yes	Yes
T113	Injury of unspecified nerve of upper limb, level unspecified	Yes	Yes
T114	Injury of unspecified blood vessel of upper limb, level unspecifi	Yes	Yes
T115	Injury of unspecified muscle and tendon of upper limb, level ur	Yes	Yes
T116	Traumatic amputation of upper limb, level unspecified	Yes	Yes
T118	Other specified injuries of upper limb, level unspecified	Yes	Yes
T119	Unspecified injury of upper limb, level unspecified	Yes	Yes
T12X	Fracture of lower limb, level unspecified	Yes	Yes
T120	Fracture of lower limb, level unspecified	Yes	Yes
T13	Other injuries of lower limb, level unspecified	Yes	Yes
T130	Superficial injury of lower limb, level unspecified	Yes	Yes
T131	Open wound of lower limb, level unspecified	Yes	Yes
T132	Dislocation, sprain and strain of unspecified joint and ligament	Yes	Yes
T133	Injury of unspecified nerve of lower limb, level unspecified	Yes	Yes
T134	Injury of unspecified blood vessel of lower limb, level unspecifi	Yes	Yes
T135	Injury of unspecified muscle and tendon of lower limb, level un	Yes	Yes
T136	Traumatic amputation of lower limb, level unspecified	Yes	Yes
T138	Other specified injuries of lower limb, level unspecified	Yes	Yes
T139	Unspecified injury of lower limb, level unspecified	Yes	Yes

T14	Injury of unspecified body region	Yes	Yes
T140	Superficial injury of unspecified body region	Yes	Yes
T141	Open wound of unspecified body region	Yes	Yes
T142	Fracture of unspecified body region	Yes	Yes
T143	Dislocation, sprain and strain of unspecified body region	Yes	Yes
T144	Injury of nerve(s) of unspecified body region	Yes	Yes
T145	Injury of blood vessel(s) of unspecified body region	Yes	Yes
T146	Injury of muscles and tendons of unspecified body region	Yes	Yes
T147	Crushing injury and traumatic amputation of unspecified body	Yes	Yes
T148	Other injuries of unspecified body region	Yes	Yes
T149	Injury, unspecified	Yes	Yes
T15	Foreign body on external eye	Yes	Yes
T150	Foreign body in cornea	Yes	Yes
T151	Foreign body in conjunctival sac	Yes	Yes
T158	Foreign body in other and multiple parts of external eye	Yes	Yes
T159	Foreign body on external eye, part unspecified	Yes	Yes
T16X	Foreign body in ear	Yes	Yes
T17	Foreign body in respiratory tract	Yes	Yes
T170	Foreign body in nasal sinus	Yes	Yes
T171	Foreign body in nostril	Yes	Yes
T172	Foreign body in pharynx	Yes	Yes
T173	Foreign body in larynx	Yes	Yes
T174	Foreign body in trachea	Yes	Yes
T175	Foreign body in bronchus	Yes	Yes
T178	Foreign body in other and multiple parts of respiratory tract	Yes	Yes
T179	Foreign body in respiratory tract, part unspecified	Yes	Yes
T18	Foreign body in alimentary tract	Yes	Yes
T180	Foreign body in mouth	Yes	Yes
T181	Foreign body in oesophagus	Yes	Yes
T182	Foreign body in stomach	Yes	Yes
T183	Foreign body in small intestine	Yes	Yes
T184	Foreign body in colon	Yes	Yes
T185	Foreign body in anus and rectum	Yes	Yes
T188	Foreign body in other and multiple parts of alimentary tract	Yes	Yes
T189	Foreign body in alimentary tract, part unspecified	Yes	Yes
T19	Foreign body in genitourinary tract	Yes	Yes
T190	Foreign body in urethra	Yes	Yes
T191	Foreign body in bladder	Yes	Yes
T192	Foreign body in vulva and vagina	Yes	Yes
T193	Foreign body in uterus [any part]	Yes	Yes
T198	Foreign body in other and multiple parts of genitourinary tract	Yes	Yes
T199	Foreign body in genitourinary tract, part unspecified	Yes	Yes
T20	Burn and corrosion of head and neck	Yes	Yes
T200	Burn of unspecified degree of head and neck	Yes	Yes
T201	Burn of first degree of head and neck	Yes	Yes
T202	Burn of second degree of head and neck	Yes	Yes
T203	Burn of third degree of head and neck	Yes	Yes
T204	Corrosion of unspecified degree of head and neck	Yes	Yes
T205	Corrosion of first degree of head and neck	Yes	Yes
T206	Corrosion of second degree of head and neck	Yes	Yes
T207	Corrosion of third degree of head and neck	Yes	Yes
T21	Burn and corrosion of trunk	Yes	Yes
T210	Burn of unspecified degree of trunk	Yes	Yes

T211	Burn of first degree of trunk	Yes	Yes
T212	Burn of second degree of trunk	Yes	Yes
T213	Burn of third degree of trunk	Yes	Yes
T214	Corrosion of unspecified degree of trunk	Yes	Yes
T215	Corrosion of first degree of trunk	Yes	Yes
T216	Corrosion of second degree of trunk	Yes	Yes
T217	Corrosion of third degree of trunk	Yes	Yes
T22	Burn and corrosion of shoulder and upper limb, except wrist an	Yes	Yes
T220	Burn of unspecified degree of shoulder and upper limb, except	Yes	Yes
T221	Burn of first degree of shoulder and upper limb, except wrist ar	Yes	Yes
T222	Burn of second degree of shoulder and upper limb, except wris	Yes	Yes
T223	Burn of third degree of shoulder and upper limb, except wrist a	Yes	Yes
T224	Corrosion of unspecified degree of shoulder and upper limb, ex	Yes	Yes
T225	Corrosion of first degree of shoulder and upper limb, except wr	Yes	Yes
T226	Corrosion of second degree of shoulder and upper limb, except	Yes	Yes
T227	Corrosion of third degree of shoulder and upper limb, except w	Yes	Yes
T23	Burn and corrosion of wrist and hand	Yes	Yes
T230	Burn of unspecified degree of wrist and hand	Yes	Yes
T231	Burn of first degree of wrist and hand	Yes	Yes
T232	Burn of second degree of wrist and hand	Yes	Yes
T233	Burn of third degree of wrist and hand	Yes	Yes
T234	Corrosion of unspecified degree of wrist and hand	Yes	Yes
T235	Corrosion of first degree of wrist and hand	Yes	Yes
T236	Corrosion of second degree of wrist and hand	Yes	Yes
T237	Corrosion of third degree of wrist and hand	Yes	Yes
T24	Burn and corrosion of hip and lower limb, except ankle and foo	Yes	Yes
T240	Burn of unspecified degree of hip and lower limb, except ankle	Yes	Yes
T241	Burn of first degree of hip and lower limb, except ankle and foc	Yes	Yes
T242	Burn of second degree of hip and lower limb, except ankle and	Yes	Yes
T243	Burn of third degree of hip and lower limb, except ankle and fo	Yes	Yes
T244	Corrosion of unspecified degree of hip and lower limb, except a	Yes	Yes
T245	Corrosion of first degree of hip and lower limb, except ankle an	Yes	Yes
T246	Corrosion of second degree of hip and lower limb, except ankle	Yes	Yes
T247	Corrosion of third degree of hip and lower limb, except ankle ar	Yes	Yes
T25	Burn and corrosion of ankle and foot	Yes	Yes
T250	Burn of unspecified degree of ankle and foot	Yes	Yes
T251	Burn of first degree of ankle and foot	Yes	Yes
T252	Burn of second degree of ankle and foot	Yes	Yes
T253	Burn of third degree of ankle and foot	Yes	Yes
T254	Corrosion of unspecified degree of ankle and foot	Yes	Yes
T255	Corrosion of first degree of ankle and foot	Yes	Yes
T256	Corrosion of second degree of ankle and foot	Yes	Yes
T257	Corrosion of third degree of ankle and foot	Yes	Yes
T26	Burn and corrosion confined to eye and adnexa	Yes	Yes
T260	Burn of eyelid and periocular area	Yes	Yes
T261	Burn of cornea and conjunctival sac	Yes	Yes
T262	Burn with resulting rupture and destruction of eyeball	Yes	Yes
T263	Burn of other parts of eye and adnexa	Yes	Yes
T264	Burn of eye and adnexa, part unspecified	Yes	Yes
T265	Corrosion of eyelid and periocular area	Yes	Yes
T266	Corrosion of cornea and conjunctival sac	Yes	Yes
T267	Corrosion with resulting rupture and destruction of eyeball	Yes	Yes
T268	Corrosion of other parts of eye and adnexa	Yes	Yes

T269	Corrosion of eye and adnexa, part unspecified	Yes	Yes
T27	Burn and corrosion of respiratory tract	Yes	Yes
T274	Corrosion of larynx and trachea	Yes	Yes
T275	Corrosion involving larynx and trachea with lung	Yes	Yes
T276	Corrosion of other parts of respiratory tract	Yes	Yes
T277	Corrosion of respiratory tract, part unspecified	Yes	Yes
T28	Burn and corrosion of other internal organs	Yes	Yes
T280	Burn of mouth and pharynx	Yes	Yes
T281	Burn of oesophagus	Yes	Yes
T282	Burn of other parts of alimentary tract	Yes	Yes
T283	Burn of internal genitourinary organs	Yes	Yes
T284	Burn of other and unspecified internal organs	Yes	Yes
T285	Corrosion of mouth and pharynx	Yes	Yes
T286	Corrosion of oesophagus	Yes	Yes
T287	Corrosion of other parts of alimentary tract	Yes	Yes
T288	Corrosion of internal genitourinary organs	Yes	Yes
T289	Corrosion of other and unspecified internal organs	Yes	Yes
T29	Burns and corrosions of multiple body regions	Yes	Yes
T290	Burns of multiple regions, unspecified degree	Yes	Yes
T291	Burns of multiple regions, no more than first-degree burns mer	Yes	Yes
T292	Burns of multiple regions, no more than second-degree burns r	Yes	Yes
T293	Burns of multiple regions, at least one burn of third degree mer	Yes	Yes
T294	Corrosions of multiple regions, unspecified degree	Yes	Yes
T295	Corrosions of multiple regions, no more than first-degree corro	Yes	Yes
T296	Corrosions of multiple regions, no more than second-degree co	Yes	Yes
T297	Corrosions of multiple regions, at least one corrosion of third d	Yes	Yes
T30	Burn and corrosion, body region unspecified	Yes	Yes
T300	Burn of unspecified body region, unspecified degree	Yes	Yes
T301	Burn of first degree, body region unspecified	Yes	Yes
T302	Burn of second degree, body region unspecified	Yes	Yes
T303	Burn of third degree, body region unspecified	Yes	Yes
T304	Corrosion of unspecified body region, unspecified degree	Yes	Yes
T305	Corrosion of first degree, body region unspecified	Yes	Yes
T306	Corrosion of second degree, body region unspecified	Yes	Yes
T307	Corrosion of third degree, body region unspecified	Yes	Yes
T31	Burns classified according to extent of body surface involved	Yes	Yes
T310	Burns involving less than 10% of body surface	Yes	Yes
T311	Burns involving 10-19% of body surface	Yes	Yes
T312	Burns involving 20-29% of body surface	Yes	Yes
T313	Burns involving 30-39% of body surface	Yes	Yes
T314	Burns involving 40-49% of body surface	Yes	Yes
T315	Burns involving 50-59% of body surface	Yes	Yes
T316	Burns involving 60-69% of body surface	Yes	Yes
T317	Burns involving 70-79% of body surface	Yes	Yes
T318	Burns involving 80-89% of body surface	Yes	Yes
T319	Burns involving 90% or more of body surface	Yes	Yes
T32	Corrosions classified according to extent of body surface involv	Yes	Yes
T320	Corrosions involving less than 10% of body surface	Yes	Yes
T321	Corrosions involving 10-19% of body surface	Yes	Yes
T322	Corrosions involving 20-29% of body surface	Yes	Yes
T323	Corrosions involving 30-39% of body surface	Yes	Yes
T324	Corrosions involving 40-49% of body surface	Yes	Yes
T325	Corrosions involving 50-59% of body surface	Yes	Yes

T326	Corrosions involving 60-69% of body surface	Yes	Yes
T327	Corrosions involving 70-79% of body surface	Yes	Yes
T328	Corrosions involving 80-89% of body surface	Yes	Yes
T329	Corrosions involving 90% or more of body surface	Yes	Yes
T36	Poisoning by systemic antibiotics	Yes	Yes
T360	Poisoning: Penicillins	Yes	Yes
T361	Poisoning: Cefalosporins and other beta-lactam antibiotics	Yes	Yes
T362	Poisoning: Chloramphenicol group	Yes	Yes
T363	Poisoning: Macrolides	Yes	Yes
T364	Poisoning: Tetracyclines	Yes	Yes
T365	Poisoning: Aminoglycosides	Yes	Yes
T366	Poisoning: Rifamycins	Yes	Yes
T367	Poisoning: Antifungal antibiotics, systemically used	Yes	Yes
T368	Poisoning: Other systemic antibiotics	Yes	Yes
T369	Poisoning: Systemic antibiotic, unspecified	Yes	Yes
T37	Poisoning by other systemic anti-infectives and antiparasitics	Yes	Yes
T370	Poisoning: Sulfonamides	Yes	Yes
T371	Poisoning: Antimycobacterial drugs	Yes	Yes
T372	Poisoning: Antimalarials and drugs acting on other blood proto	Yes	Yes
T373	Poisoning: Other antiprotozoal drugs	Yes	Yes
T374	Poisoning: Anthelminthics	Yes	Yes
T375	Poisoning: Antiviral drugs	Yes	Yes
T378	Poisoning: Other specified systemic anti-infectives and antipara	Yes	Yes
T379	Poisoning: Systemic anti-infective and antiparasitic, unspecified	Yes	Yes
T38	Poisoning by hormones and their synthetic substitutes and ant	Yes	Yes
T380	Poisoning: Glucocorticoids and synthetic analogues	Yes	Yes
T381	Poisoning: Thyroid hormones and substitutes	Yes	Yes
T382	Poisoning: Antithyroid drugs	Yes	Yes
T383	Poisoning: Insulin and oral hypoglycaemic [antidiabetic] drugs	Yes	Yes
T384	Poisoning: Oral contraceptives	Yes	Yes
T385	Poisoning: Other estrogens and progestogens	Yes	Yes
T386	Poisoning: Antigonadotrophins, antiestrogens, antiandrogens, r	Yes	Yes
T387	Poisoning: Androgens and anabolic congeners	Yes	Yes
T388	Poisoning: Other and unspecified hormones and their synthetic	Yes	Yes
T389	Poisoning: Other and unspecified hormone antagonists	Yes	Yes
T39	Poisoning by nonopioid analgesics, antipyretics and antirheuma	Yes	Yes
T390	Poisoning: Salicylates	Yes	Yes
T391	Poisoning: 4-Aminophenol derivatives	Yes	Yes
T392	Poisoning: Pyrazolone derivatives	Yes	Yes
T393	Poisoning: Other nonsteroidal anti-inflammatory drugs [NSAID]	Yes	Yes
T394	Poisoning: Antirheumatics, not elsewhere classified	Yes	Yes
T398	Poisoning: Other nonopioid analgesics and antipyretics, not els	Yes	Yes
T399	Poisoning: Nonopioid analgesic, antipyretic and antirheumatic,	Yes	Yes
T40	Poisoning by narcotics and psychodysleptics [hallucinogens]	Yes	Yes
T400	Poisoning: Opium	Yes	Yes
T401	Poisoning: Heroin	Yes	Yes
T402	Poisoning: Other opioids	Yes	Yes
T403	Poisoning: Methadone	Yes	Yes
T404	Poisoning: Other synthetic narcotics	Yes	Yes
T405	Poisoning: Cocaine	Yes	Yes
T406	Poisoning: Other and unspecified narcotics	Yes	Yes
T407	Poisoning: Cannabis (derivatives)	Yes	Yes
T408	Poisoning: Lysergide [LSD]	Yes	Yes

T409	Poisoning: Other and unspecified psychodysleptics [hallucinoge	Yes	Yes
T41	Poisoning by anaesthetics and therapeutic gases	Yes	Yes
T410	Poisoning: Inhaled anaesthetics	Yes	Yes
T411	Poisoning: Intravenous anaesthetics	Yes	Yes
T412	Poisoning: Other and unspecified general anaesthetics	Yes	Yes
T413	Poisoning: Local anaesthetics	Yes	Yes
T414	Poisoning: Anaesthetic, unspecified	Yes	Yes
T415	Poisoning: Therapeutic gases	Yes	Yes
T42	Poisoning by antiepileptic, sedative-hypnotic and antiparkinsor	Yes	Yes
T420	Poisoning: Hydantoin derivatives	Yes	Yes
T421	Poisoning: Iminostilbenes	Yes	Yes
T422	Poisoning: Succinimides and oxazolidinediones	Yes	Yes
T423	Poisoning: Barbiturates	Yes	Yes
T424	Poisoning: Benzodiazepines	Yes	Yes
T425	Poisoning: Mixed antiepileptics, not elsewhere classified	Yes	Yes
T426	Poisoning: Other antiepileptic and sedative-hypnotic drugs	Yes	Yes
T427	Poisoning: Antiepileptic and sedative-hypnotic drugs, unspecifi	Yes	Yes
T428	Poisoning: Antiparkinsonism drugs and other central muscle-to	Yes	Yes
T43	Poisoning by psychotropic drugs, not elsewhere classified	Yes	Yes
T430	Poisoning: Tricyclic and tetracyclic antidepressants	Yes	Yes
T431	Poisoning: Monoamine-oxidase-inhibitor antidepressants	Yes	Yes
T432	Poisoning: Other and unspecified antidepressants	Yes	Yes
T433	Poisoning: Phenothiazine antipsychotics and neuroleptics	Yes	Yes
T434	Poisoning: Butyrophenone and thioxanthene neuroleptics	Yes	Yes
T435	Poisoning: Other and unspecified antipsychotics and neurolept	Yes	Yes
T436	Poisoning: Psychostimulants with abuse potential	Yes	Yes
T438	Poisoning: Other psychotropic drugs, not elsewhere classified	Yes	Yes
T439	Poisoning: Psychotropic drug, unspecified	Yes	Yes
T44	Poisoning by drugs primarily affecting the autonomic nervous s	Yes	Yes
T440	Poisoning: Anticholinesterase agents	Yes	Yes
T441	Poisoning: Other parasympathomimetics [cholinergics]	Yes	Yes
T442	Poisoning: Ganglionic blocking drugs, not elsewhere classified	Yes	Yes
T443	Poisoning: Other parasympatholytics [anticholinergics and anti	Yes	Yes
T444	Poisoning: Predominantly alpha-adrenoreceptor agonists, not e	Yes	Yes
T445	Poisoning: Predominantly beta-adrenoreceptor agonists, not el	Yes	Yes
T446	Poisoning: Alpha-adrenoreceptor antagonists, not elsewhere cl	Yes	Yes
T447	Poisoning: Beta-adrenoreceptor antagonists, not elsewhere cla	Yes	Yes
T448	Poisoning: Centrally acting and adrenergic-neuron-blocking age	Yes	Yes
T449	Poisoning: Other and unspecified drugs primarily affecting the a	Yes	Yes
T45	Poisoning by primarily systemic and haematological agents, not	Yes	Yes
T450	Poisoning: Antiallergic and antiemetic drugs	Yes	Yes
T451	Poisoning: Antineoplastic and immunosuppressive drugs	Yes	Yes
T452	Poisoning: Vitamins, not elsewhere classified	Yes	Yes
T453	Poisoning: Enzymes, not elsewhere classified	Yes	Yes
T454	Poisoning: Iron and its compounds	Yes	Yes
T455	Poisoning: Anticoagulants	Yes	Yes
T456	Poisoning: Fibrinolysis-affecting drugs	Yes	Yes
T457	Poisoning: Anticoagulant antagonists, vitamin K and other coag	Yes	Yes
T458	Poisoning: Other primarily systemic and haematological agents	Yes	Yes
T459	Poisoning: Primarily systemic and haematological agent, unspe	Yes	Yes
T46	Poisoning by agents primarily affecting the cardiovascular syste	Yes	Yes
T460	Poisoning: Cardiac-stimulant glycosides and drugs of similar act	Yes	Yes
T461	Poisoning: Calcium-channel blockers	Yes	Yes

T462	Poisoning: Other antidysrhythmic drugs, not elsewhere classifie	Yes	Yes
T463	Poisoning: Coronary vasodilators, not elsewhere classified	Yes	Yes
T464	Poisoning: Angiotensin-converting-enzyme inhibitors	Yes	Yes
T465	Poisoning: Other antihypertensive drugs, not elsewhere classifi	Yes	Yes
T466	Poisoning: Antihyperlipidaemic and antiarteriosclerotic drugs	Yes	Yes
T467	Poisoning: Peripheral vasodilators	Yes	Yes
T468	Poisoning: Antivaricose drugs, including sclerosing agents	Yes	Yes
T469	Poisoning: Other and unspecified agents primarily affecting the	Yes	Yes
T47	Poisoning by agents primarily affecting the gastrointestinal syst	Yes	Yes
T470	Poisoning: Histamine H2-receptor antagonists	Yes	Yes
T471	Poisoning: Other antacids and anti-gastric-secretion drugs	Yes	Yes
T472	Poisoning: Stimulant laxatives	Yes	Yes
T473	Poisoning: Saline and osmotic laxatives	Yes	Yes
T474	Poisoning: Other laxatives	Yes	Yes
T475	Poisoning: Digestants	Yes	Yes
T476	Poisoning: Antidiarrhoeal drugs	Yes	Yes
T477	Poisoning: Emetics	Yes	Yes
T478	Poisoning: Other agents primarily affecting the gastrointestinal	Yes	Yes
T479	Poisoning: Agent primarily affecting the gastrointestinal system	Yes	Yes
T48	Poisoning by agents primarily acting on smooth and skeletal mu	Yes	Yes
T480	Poisoning: Oxytocic drugs	Yes	Yes
T481	Poisoning: Skeletal muscle relaxants [neuromuscular blocking a	Yes	Yes
T482	Poisoning: Other and unspecified agents primarily acting on mu	Yes	Yes
T483	Poisoning: Antitussives	Yes	Yes
T484	Poisoning: Expectorants	Yes	Yes
T485	Poisoning: Anti-common-cold drugs	Yes	Yes
T486	Poisoning: Antiasthmatics, not elsewhere classified	Yes	Yes
T487	Poisoning: Other and unspecified agents primarily acting on the	Yes	Yes
T49	Poisoning by topical agents primarily affecting skin and mucous	Yes	Yes
T490	Poisoning: Local antifungal, anti-infective and anti-inflammator	Yes	Yes
T491	Poisoning: Antipruritics	Yes	Yes
T492	Poisoning: Local astringents and local detergents	Yes	Yes
T493	Poisoning: Emollients, demulcents and protectants	Yes	Yes
T494	Poisoning: Keratolytics, keratoplastics and other hair treatment	Yes	Yes
T495	Poisoning: Ophthalmological drugs and preparations	Yes	Yes
T496	Poisoning: Otorhinolaryngological drugs and preparations	Yes	Yes
T497	Poisoning: Dental drugs, topically applied	Yes	Yes
T498	Poisoning: Other topical agents	Yes	Yes
T499	Poisoning: Topical agent, unspecified	Yes	Yes
T50	Poisoning by diuretics and other and unspecified drugs, medica	Yes	Yes
T500	Poisoning: Mineralocorticoids and their antagonists	Yes	Yes
T501	Poisoning: Loop [high-ceiling] diuretics	Yes	Yes
T502	Poisoning: Carbonic-anhydrase inhibitors, benzothiadiazides an	Yes	Yes
T503	Poisoning: Electrolytic, caloric and water-balance agents	Yes	Yes
T504	Poisoning: Drugs affecting uric acid metabolism	Yes	Yes
T505	Poisoning: Appetite depressants	Yes	Yes
T506	Poisoning: Antidotes and chelating agents, not elsewhere classi	Yes	Yes
T507	Poisoning: Analeptics and opioid receptor antagonists	Yes	Yes
T508	Poisoning: Diagnostic agents	Yes	Yes
T509	Poisoning: Other and unspecified drugs, medicaments and biolo	Yes	Yes
T51	Toxic effect of alcohol	Yes	Yes
T510	Toxic effect: Ethanol	Yes	Yes
T511	Toxic effect: Methanol	Yes	Yes

T512	Toxic effect: 2-Propanol	Yes	Yes
T513	Toxic effect: Fusel oil	Yes	Yes
T518	Toxic effect: Other alcohols	Yes	Yes
T519	Toxic effect: Alcohol, unspecified	Yes	Yes
T52	Toxic effect of organic solvents	Yes	Yes
T520	Toxic effect: Petroleum products	Yes	Yes
T521	Toxic effect: Benzene	Yes	Yes
T522	Toxic effect: Homologues of benzene	Yes	Yes
T523	Toxic effect: Glycols	Yes	Yes
T524	Toxic effect: Ketones	Yes	Yes
T528	Toxic effect: Other organic solvents	Yes	Yes
T529	Toxic effect: Organic solvent, unspecified	Yes	Yes
T53	Toxic effect of halogen derivatives of aliphatic and aromatic hy	Yes	Yes
T530	Toxic effect: Carbon tetrachloride	Yes	Yes
T531	Toxic effect: Chloroform	Yes	Yes
T532	Toxic effect: Trichloroethylene	Yes	Yes
T533	Toxic effect: Tetrachloroethylene	Yes	Yes
T534	Toxic effect: Dichloromethane	Yes	Yes
T535	Toxic effect: Chlorofluorocarbons	Yes	Yes
T536	Toxic effect: Other halogen derivatives of aliphatic hydrocarbor	Yes	Yes
T537	Toxic effect: Other halogen derivatives of aromatic hydrocarbo	Yes	Yes
T539	Toxic effect: Halogen derivative of aliphatic and aromatic hydro	Yes	Yes
T54	Toxic effect of corrosive substances	Yes	Yes
T540	Toxic effect: Phenol and phenol homologues	Yes	Yes
T541	Toxic effect: Other corrosive organic compounds	Yes	Yes
T542	Toxic effect: Corrosive acids and acid-like substances	Yes	Yes
T543	Toxic effect: Corrosive alkalis and alkali-like substances	Yes	Yes
T549	Toxic effect: Corrosive substance, unspecified	Yes	Yes
T55X	Toxic effect of soaps and detergents	Yes	Yes
T56	Toxic effect of metals	Yes	Yes
T560	Toxic effect: Lead and its compounds	Yes	Yes
T561	Toxic effect: Mercury and its compounds	Yes	Yes
T562	Toxic effect: Chromium and its compounds	Yes	Yes
T563	Toxic effect: Cadmium and its compounds	Yes	Yes
T564	Toxic effect: Copper and its compounds	Yes	Yes
T565	Toxic effect: Zinc and its compounds	Yes	Yes
T566	Toxic effect: Tin and its compounds	Yes	Yes
T567	Toxic effect: Beryllium and its compounds	Yes	Yes
T568	Toxic effect: Other metals	Yes	Yes
T569	Toxic effect: Metal, unspecified	Yes	Yes
T57	Toxic effect of other inorganic substances	Yes	Yes
T570	Toxic effect: Arsenic and its compounds	Yes	Yes
T571	Toxic effect: Phosphorus and its compounds	Yes	Yes
T572	Toxic effect: Manganese and its compounds	Yes	Yes
T573	Toxic effect: Hydrogen cyanide	Yes	Yes
T578	Toxic effect: Other specified inorganic substances	Yes	Yes
T579	Toxic effect: Inorganic substance, unspecified	Yes	Yes
T58X	Toxic effect of carbon monoxide	Yes	Yes
T59	Toxic effect of other gases, fumes and vapours	Yes	Yes
T590	Toxic effect: Nitrogen oxides	Yes	Yes
T591	Toxic effect: Sulfur dioxide	Yes	Yes
T592	Toxic effect: Formaldehyde	Yes	Yes
T593	Toxic effect: Lacrimogenic gas	Yes	Yes

T594	Toxic effect: Chlorine gas	Yes	Yes
T595	Toxic effect: Fluorine gas and hydrogen fluoride	Yes	Yes
T596	Toxic effect: Hydrogen sulfide	Yes	Yes
T597	Toxic effect: Carbon dioxide	Yes	Yes
T598	Toxic effect: Other specified gases, fumes and vapours	Yes	Yes
T599	Toxic effect: Gases, fumes and vapours, unspecified	Yes	Yes
T60	Toxic effect of pesticides	Yes	Yes
T600	Toxic effect: Organophosphate and carbamate insecticides	Yes	Yes
T601	Toxic effect: Halogenated insecticides	Yes	Yes
T602	Toxic effect: Other insecticides	Yes	Yes
T603	Toxic effect: Herbicides and fungicides	Yes	Yes
T604	Toxic effect: Rodenticides	Yes	Yes
T608	Toxic effect: Other pesticides	Yes	Yes
T609	Toxic effect: Pesticide, unspecified	Yes	Yes
T65	Toxic effect of other and unspecified substances	Yes	Yes
T650	Toxic effect: Cyanides	Yes	Yes
T651	Toxic effect: Strychnine and its salts	Yes	Yes
T652	Toxic effect: Tobacco and nicotine	Yes	Yes
T653	Toxic effect: Nitroderivatives and aminoderivatives of benzene	Yes	Yes
T654	Toxic effect: Carbon disulfide	Yes	Yes
T655	Toxic effect: Nitroglycerin and other nitric acids and esters	Yes	Yes
T656	Toxic effect: Paints and dyes, not elsewhere classified	Yes	Yes
T658	Toxic effect: Toxic effect of other specified substances	Yes	Yes
T659	Toxic effect: Toxic effect of unspecified substance	Yes	Yes
T751	Drowning and nonfatal submersion	Yes	Yes
W10	Fall on and from stairs and steps	Yes	Yes
W100	Fall on and from stairs and steps	Yes	Yes
W101	Fall on and from stairs and steps	Yes	Yes
W102	Fall on and from stairs and steps	Yes	Yes
W103	Fall on and from stairs and steps	Yes	Yes
W104	Fall on and from stairs and steps	Yes	Yes
W105	Fall on and from stairs and steps	Yes	Yes
W106	Fall on and from stairs and steps	Yes	Yes
W107	Fall on and from stairs and steps	Yes	Yes
W108	Fall on and from stairs and steps	Yes	Yes
W109	Fall on and from stairs and steps	Yes	Yes
W13	Fall from, out of or through building or structure	Yes	Yes
W130	Fall from, out of or through building or structure	Yes	Yes
W131	Fall from, out of or through building or structure	Yes	Yes
W132	Fall from, out of or through building or structure	Yes	Yes
W133	Fall from, out of or through building or structure	Yes	Yes
W134	Fall from, out of or through building or structure	Yes	Yes
W135	Fall from, out of or through building or structure	Yes	Yes
W136	Fall from, out of or through building or structure	Yes	Yes
W137	Fall from, out of or through building or structure	Yes	Yes
W138	Fall from, out of or through building or structure	Yes	Yes
W139	Fall from, out of or through building or structure	Yes	Yes
W65	Drowning and submersion while in bath-tub	Yes	Yes
W650	Drowning and submersion while in bath-tub	Yes	Yes
W651	Drowning and submersion while in bath-tub	Yes	Yes
W652	Drowning and submersion while in bath-tub	Yes	Yes
W653	Drowning and submersion while in bath-tub	Yes	Yes
W654	Drowning and submersion while in bath-tub	Yes	Yes

W655	Drowning and submersion while in bath-tub	Yes	Yes
W656	Drowning and submersion while in bath-tub	Yes	Yes
W657	Drowning and submersion while in bath-tub	Yes	Yes
W658	Drowning and submersion while in bath-tub	Yes	Yes
W659	Drowning and submersion while in bath-tub	Yes	Yes
X02	Exposure to controlled fire in building or structure	Yes	Yes
X020	Exposure to controlled fire in building or structure	Yes	Yes
X021	Exposure to controlled fire in building or structure	Yes	Yes
X022	Exposure to controlled fire in building or structure	Yes	Yes
X023	Exposure to controlled fire in building or structure	Yes	Yes
X024	Exposure to controlled fire in building or structure	Yes	Yes
X025	Exposure to controlled fire in building or structure	Yes	Yes
X026	Exposure to controlled fire in building or structure	Yes	Yes
X027	Exposure to controlled fire in building or structure	Yes	Yes
X028	Exposure to controlled fire in building or structure	Yes	Yes
X029	Exposure to controlled fire in building or structure	Yes	Yes
X40	Accidental poisoning by and exposure to nonopioid analgesics,	Yes	Yes
X400	Accidental poisoning by and exposure to nonopioid analgesics,	Yes	Yes
X401	Accidental poisoning by and exposure to nonopioid analgesics,	Yes	Yes
X402	Accidental poisoning by and exposure to nonopioid analgesics,	Yes	Yes
X403	Accidental poisoning by and exposure to nonopioid analgesics,	Yes	Yes
X404	Accidental poisoning by and exposure to nonopioid analgesics,	Yes	Yes
X405	Accidental poisoning by and exposure to nonopioid analgesics,	Yes	Yes
X406	Accidental poisoning by and exposure to nonopioid analgesics,	Yes	Yes
X407	Accidental poisoning by and exposure to nonopioid analgesics,	Yes	Yes
X408	Accidental poisoning by and exposure to nonopioid analgesics,	Yes	Yes
X409	Accidental poisoning by and exposure to nonopioid analgesics,	Yes	Yes
X41	Accidental poisoning by and exposure to antiepileptic, sedative	Yes	Yes
X410	Accidental poisoning by and exposure to antiepileptic, sedative	Yes	Yes
X411	Accidental poisoning by and exposure to antiepileptic, sedative	Yes	Yes
X412	Accidental poisoning by and exposure to antiepileptic, sedative	Yes	Yes
X413	Accidental poisoning by and exposure to antiepileptic, sedative	Yes	Yes
X414	Accidental poisoning by and exposure to antiepileptic, sedative	Yes	Yes
X415	Accidental poisoning by and exposure to antiepileptic, sedative	Yes	Yes
X416	Accidental poisoning by and exposure to antiepileptic, sedative	Yes	Yes
X417	Accidental poisoning by and exposure to antiepileptic, sedative	Yes	Yes
X418	Accidental poisoning by and exposure to antiepileptic, sedative	Yes	Yes
X419	Accidental poisoning by and exposure to antiepileptic, sedative	Yes	Yes
X42	Accidental poisoning by and exposure to narcotics and psychod	Yes	Yes
X420	Accidental poisoning by and exposure to narcotics and psychod	Yes	Yes
X421	Accidental poisoning by and exposure to narcotics and psychod	Yes	Yes
X422	Accidental poisoning by and exposure to narcotics and psychod	Yes	Yes
X423	Accidental poisoning by and exposure to narcotics and psychod	Yes	Yes
X424	Accidental poisoning by and exposure to narcotics and psychod	Yes	Yes
X425	Accidental poisoning by and exposure to narcotics and psychod	Yes	Yes
X426	Accidental poisoning by and exposure to narcotics and psychod	Yes	Yes
X427	Accidental poisoning by and exposure to narcotics and psychod	Yes	Yes
X428	Accidental poisoning by and exposure to narcotics and psychod	Yes	Yes
X429	Accidental poisoning by and exposure to narcotics and psychod	Yes	Yes
X43	Accidental poisoning by and exposure to other drugs acting on	Yes	Yes
X430	Accidental poisoning by and exposure to other drugs acting on	Yes	Yes
X431	Accidental poisoning by and exposure to other drugs acting on	Yes	Yes
X432	Accidental poisoning by and exposure to other drugs acting on	Yes	Yes

X433	Accidental poisoning by and exposure to other drugs acting on	Yes	Yes
X434	Accidental poisoning by and exposure to other drugs acting on	Yes	Yes
X435	Accidental poisoning by and exposure to other drugs acting on	Yes	Yes
X436	Accidental poisoning by and exposure to other drugs acting on	Yes	Yes
X437	Accidental poisoning by and exposure to other drugs acting on	Yes	Yes
X438	Accidental poisoning by and exposure to other drugs acting on	Yes	Yes
X439	Accidental poisoning by and exposure to other drugs acting on	Yes	Yes
X44	Accidental poisoning by and exposure to other and unspecified	Yes	Yes
X440	Accidental poisoning by and exposure to other and unspecified	Yes	Yes
X441	Accidental poisoning by and exposure to other and unspecified	Yes	Yes
X442	Accidental poisoning by and exposure to other and unspecified	Yes	Yes
X443	Accidental poisoning by and exposure to other and unspecified	Yes	Yes
X444	Accidental poisoning by and exposure to other and unspecified	Yes	Yes
X445	Accidental poisoning by and exposure to other and unspecified	Yes	Yes
X446	Accidental poisoning by and exposure to other and unspecified	Yes	Yes
X447	Accidental poisoning by and exposure to other and unspecified	Yes	Yes
X448	Accidental poisoning by and exposure to other and unspecified	Yes	Yes
X449	Accidental poisoning by and exposure to other and unspecified	Yes	Yes
X45	Accidental poisoning by and exposure to alcohol	Yes	Yes
X450	Accidental poisoning by and exposure to alcohol	Yes	Yes
X451	Accidental poisoning by and exposure to alcohol	Yes	Yes
X452	Accidental poisoning by and exposure to alcohol	Yes	Yes
X453	Accidental poisoning by and exposure to alcohol	Yes	Yes
X454	Accidental poisoning by and exposure to alcohol	Yes	Yes
X455	Accidental poisoning by and exposure to alcohol	Yes	Yes
X456	Accidental poisoning by and exposure to alcohol	Yes	Yes
X457	Accidental poisoning by and exposure to alcohol	Yes	Yes
X458	Accidental poisoning by and exposure to alcohol	Yes	Yes
X459	Accidental poisoning by and exposure to alcohol	Yes	Yes
X46	Accidental poisoning by and exposure to organic solvents and h	Yes	Yes
X460	Accidental poisoning by and exposure to organic solvents and h	Yes	Yes
X461	Accidental poisoning by and exposure to organic solvents and h	Yes	Yes
X462	Accidental poisoning by and exposure to organic solvents and h	Yes	Yes
X463	Accidental poisoning by and exposure to organic solvents and h	Yes	Yes
X464	Accidental poisoning by and exposure to organic solvents and h	Yes	Yes
X465	Accidental poisoning by and exposure to organic solvents and h	Yes	Yes
X466	Accidental poisoning by and exposure to organic solvents and h	Yes	Yes
X467	Accidental poisoning by and exposure to organic solvents and h	Yes	Yes
X468	Accidental poisoning by and exposure to organic solvents and h	Yes	Yes
X469	Accidental poisoning by and exposure to organic solvents and h	Yes	Yes
X47	Accidental poisoning by and exposure to other gases and vapou	Yes	Yes
X470	Accidental poisoning by and exposure to other gases and vapou	Yes	Yes
X471	Accidental poisoning by and exposure to other gases and vapou	Yes	Yes
X472	Accidental poisoning by and exposure to other gases and vapou	Yes	Yes
X473	Accidental poisoning by and exposure to other gases and vapou	Yes	Yes
X474	Accidental poisoning by and exposure to other gases and vapou	Yes	Yes
X475	Accidental poisoning by and exposure to other gases and vapou	Yes	Yes
X476	Accidental poisoning by and exposure to other gases and vapou	Yes	Yes
X477	Accidental poisoning by and exposure to other gases and vapou	Yes	Yes
X478	Accidental poisoning by and exposure to other gases and vapou	Yes	Yes
X479	Accidental poisoning by and exposure to other gases and vapou	Yes	Yes
X48	Accidental poisoning by and exposure to pesticides	Yes	Yes
X480	Accidental poisoning by and exposure to pesticides	Yes	Yes

X481	Accidental poisoning by and exposure to pesticides	Yes	Yes
X482	Accidental poisoning by and exposure to pesticides	Yes	Yes
X483	Accidental poisoning by and exposure to pesticides	Yes	Yes
X484	Accidental poisoning by and exposure to pesticides	Yes	Yes
X485	Accidental poisoning by and exposure to pesticides	Yes	Yes
X486	Accidental poisoning by and exposure to pesticides	Yes	Yes
X487	Accidental poisoning by and exposure to pesticides	Yes	Yes
X488	Accidental poisoning by and exposure to pesticides	Yes	Yes
X489	Accidental poisoning by and exposure to pesticides	Yes	Yes
X49	Accidental poisoning by and exposure to other and unspecified	Yes	Yes
X490	Accidental poisoning by and exposure to other and unspecified	Yes	Yes
X491	Accidental poisoning by and exposure to other and unspecified	Yes	Yes
X492	Accidental poisoning by and exposure to other and unspecified	Yes	Yes
X493	Accidental poisoning by and exposure to other and unspecified	Yes	Yes
X494	Accidental poisoning by and exposure to other and unspecified	Yes	Yes
X495	Accidental poisoning by and exposure to other and unspecified	Yes	Yes
X496	Accidental poisoning by and exposure to other and unspecified	Yes	Yes
X497	Accidental poisoning by and exposure to other and unspecified	Yes	Yes
X498	Accidental poisoning by and exposure to other and unspecified	Yes	Yes
X499	Accidental poisoning by and exposure to other and unspecified	Yes	Yes
Y90	Evidence of alcohol involvement determined by blood alcohol l	Yes	Yes
Y900	Blood alcohol level of less than 20 mg/100 ml	Yes	Yes
Y901	Blood alcohol level of 20-39 mg/100 ml	Yes	Yes
Y902	Blood alcohol level of 40-59 mg/100 ml	Yes	Yes
Y903	Blood alcohol level of 60-79 mg/100 ml	Yes	Yes
Y904	Blood alcohol level of 80-99 mg/100 ml	Yes	Yes
Y905	Blood alcohol level of 100-119 mg/100 ml	Yes	Yes
Y906	Blood alcohol level of 120-199 mg/100 ml	Yes	Yes
Y907	Blood alcohol level of 200-239 mg/100 ml	Yes	Yes
Y908	Blood alcohol level of 240 mg/100 ml or more	Yes	Yes
Y909	Presence of alcohol in blood, level not specified	Yes	Yes
Y91	Evidence of alcohol involvement determined by level of intoxic	Yes	Yes
Y910	Mild alcohol intoxication	Yes	Yes
Y911	Moderate alcohol intoxication	Yes	Yes
Y912	Severe alcohol intoxication	Yes	Yes
Y913	Very severe alcohol intoxication	Yes	Yes
Y919	Alcohol involvement, not otherwise specified	Yes	Yes
S143	Injury of brachial plexus	Yes	No
T270	Burn of larynx and trachea	Yes	No
T271	Burn involving larynx and trachea with lung	Yes	No
T272	Burn of other parts of respiratory tract	Yes	No
T273	Burn of respiratory tract, part unspecified	Yes	No
T63	Toxic effect of contact with venomous animals	Yes	No
T630	Toxic effect: Snake venom	Yes	No
T631	Toxic effect: Venom of other reptiles	Yes	No
T632	Toxic effect: Venom of scorpion	Yes	No
T633	Toxic effect: Venom of spider	Yes	No
T634	Toxic effect: Venom of other arthropods	Yes	No
T635	Toxic effect: Toxic effect of contact with fish	Yes	No
T636	Toxic effect: Toxic effect of contact with other marine animals	Yes	No
T638	Toxic effect: Toxic effect of contact with other venomous anima	Yes	No
T639	Toxic effect: Toxic effect of contact with unspecified venomous	Yes	No
T69	Other effects of reduced temperature	Yes	No

T690	Immersion hand and foot	Yes	No
T71X	Asphyxiation	Yes	No
T754	Effects of electric current	Yes	No
W00	Fall on same level involving ice and snow	Yes	No
W000	Fall on same level involving ice and snow	Yes	No
W001	Fall on same level involving ice and snow	Yes	No
W002	Fall on same level involving ice and snow	Yes	No
W003	Fall on same level involving ice and snow	Yes	No
W004	Fall on same level involving ice and snow	Yes	No
W005	Fall on same level involving ice and snow	Yes	No
W006	Fall on same level involving ice and snow	Yes	No
W007	Fall on same level involving ice and snow	Yes	No
W008	Fall on same level involving ice and snow	Yes	No
W009	Fall on same level involving ice and snow	Yes	No
W01	Fall on same level from slipping, tripping and stumbling	Yes	No
W010	Fall on same level from slipping, tripping and stumbling	Yes	No
W011	Fall on same level from slipping, tripping and stumbling	Yes	No
W012	Fall on same level from slipping, tripping and stumbling	Yes	No
W013	Fall on same level from slipping, tripping and stumbling	Yes	No
W014	Fall on same level from slipping, tripping and stumbling	Yes	No
W015	Fall on same level from slipping, tripping and stumbling	Yes	No
W016	Fall on same level from slipping, tripping and stumbling	Yes	No
W017	Fall on same level from slipping, tripping and stumbling	Yes	No
W018	Fall on same level from slipping, tripping and stumbling	Yes	No
W019	Fall on same level from slipping, tripping and stumbling	Yes	No
W03	Other fall on same level due to collision with, or pushing by, an	Yes	No
W030	Other fall on same level due to collision with, or pushing by, an	Yes	No
W031	Other fall on same level due to collision with, or pushing by, an	Yes	No
W032	Other fall on same level due to collision with, or pushing by, an	Yes	No
W033	Other fall on same level due to collision with, or pushing by, an	Yes	No
W034	Other fall on same level due to collision with, or pushing by, an	Yes	No
W035	Other fall on same level due to collision with, or pushing by, an	Yes	No
W036	Other fall on same level due to collision with, or pushing by, an	Yes	No
W037	Other fall on same level due to collision with, or pushing by, an	Yes	No
W038	Other fall on same level due to collision with, or pushing by, an	Yes	No
W039	Other fall on same level due to collision with, or pushing by, an	Yes	No
W04	Fall while being carried or supported by other persons	Yes	No
W040	Fall while being carried or supported by other persons	Yes	No
W041	Fall while being carried or supported by other persons	Yes	No
W042	Fall while being carried or supported by other persons	Yes	No
W043	Fall while being carried or supported by other persons	Yes	No
W044	Fall while being carried or supported by other persons	Yes	No
W045	Fall while being carried or supported by other persons	Yes	No
W046	Fall while being carried or supported by other persons	Yes	No
W047	Fall while being carried or supported by other persons	Yes	No
W048	Fall while being carried or supported by other persons	Yes	No
W049	Fall while being carried or supported by other persons	Yes	No
W05	Fall involving wheelchair	Yes	No
W050	Fall involving wheelchair	Yes	No
W051	Fall involving wheelchair	Yes	No
W052	Fall involving wheelchair	Yes	No
W053	Fall involving wheelchair	Yes	No
W054	Fall involving wheelchair	Yes	No

W055	Fall involving wheelchair	Yes	No
W056	Fall involving wheelchair	Yes	No
W057	Fall involving wheelchair	Yes	No
W058	Fall involving wheelchair	Yes	No
W059	Fall involving wheelchair	Yes	No
W06	Fall involving bed	Yes	No
W060	Fall involving bed	Yes	No
W061	Fall involving bed	Yes	No
W062	Fall involving bed	Yes	No
W063	Fall involving bed	Yes	No
W064	Fall involving bed	Yes	No
W065	Fall involving bed	Yes	No
W066	Fall involving bed	Yes	No
W067	Fall involving bed	Yes	No
W068	Fall involving bed	Yes	No
W069	Fall involving bed	Yes	No
W07	Fall involving chair	Yes	No
W070	Fall involving chair	Yes	No
W071	Fall involving chair	Yes	No
W072	Fall involving chair	Yes	No
W073	Fall involving chair	Yes	No
W074	Fall involving chair	Yes	No
W075	Fall involving chair	Yes	No
W076	Fall involving chair	Yes	No
W077	Fall involving chair	Yes	No
W078	Fall involving chair	Yes	No
W079	Fall involving chair	Yes	No
W08	Fall involving other furniture	Yes	No
W080	Fall involving other furniture	Yes	No
W081	Fall involving other furniture	Yes	No
W082	Fall involving other furniture	Yes	No
W083	Fall involving other furniture	Yes	No
W084	Fall involving other furniture	Yes	No
W085	Fall involving other furniture	Yes	No
W086	Fall involving other furniture	Yes	No
W087	Fall involving other furniture	Yes	No
W088	Fall involving other furniture	Yes	No
W089	Fall involving other furniture	Yes	No
W11	Fall on and from ladder	Yes	No
W110	Fall on and from ladder	Yes	No
W111	Fall on and from ladder	Yes	No
W112	Fall on and from ladder	Yes	No
W113	Fall on and from ladder	Yes	No
W114	Fall on and from ladder	Yes	No
W115	Fall on and from ladder	Yes	No
W116	Fall on and from ladder	Yes	No
W117	Fall on and from ladder	Yes	No
W118	Fall on and from ladder	Yes	No
W119	Fall on and from ladder	Yes	No
W12	Fall on and from scaffolding	Yes	No
W120	Fall on and from scaffolding	Yes	No
W121	Fall on and from scaffolding	Yes	No
W122	Fall on and from scaffolding	Yes	No

W123	Fall on and from scaffolding	Yes	No
W124	Fall on and from scaffolding	Yes	No
W125	Fall on and from scaffolding	Yes	No
W126	Fall on and from scaffolding	Yes	No
W127	Fall on and from scaffolding	Yes	No
W128	Fall on and from scaffolding	Yes	No
W129	Fall on and from scaffolding	Yes	No
W14	Fall from tree	Yes	No
W140	Fall from tree	Yes	No
W141	Fall from tree	Yes	No
W142	Fall from tree	Yes	No
W143	Fall from tree	Yes	No
W144	Fall from tree	Yes	No
W145	Fall from tree	Yes	No
W146	Fall from tree	Yes	No
W147	Fall from tree	Yes	No
W148	Fall from tree	Yes	No
W149	Fall from tree	Yes	No
W17	Other fall from one level to another	Yes	No
W170	Other fall from one level to another	Yes	No
W171	Other fall from one level to another	Yes	No
W172	Other fall from one level to another	Yes	No
W173	Other fall from one level to another	Yes	No
W174	Other fall from one level to another	Yes	No
W175	Other fall from one level to another	Yes	No
W176	Other fall from one level to another	Yes	No
W177	Other fall from one level to another	Yes	No
W178	Other fall from one level to another	Yes	No
W179	Other fall from one level to another	Yes	No
W18	Other fall on same level	Yes	No
W180	Other fall on same level	Yes	No
W181	Other fall on same level	Yes	No
W182	Other fall on same level	Yes	No
W183	Other fall on same level	Yes	No
W184	Other fall on same level	Yes	No
W185	Other fall on same level	Yes	No
W186	Other fall on same level	Yes	No
W187	Other fall on same level	Yes	No
W188	Other fall on same level	Yes	No
W189	Other fall on same level	Yes	No
W19	Unspecified fall	Yes	No
W190	Unspecified fall	Yes	No
W191	Unspecified fall	Yes	No
W192	Unspecified fall	Yes	No
W193	Unspecified fall	Yes	No
W194	Unspecified fall	Yes	No
W195	Unspecified fall	Yes	No
W196	Unspecified fall	Yes	No
W197	Unspecified fall	Yes	No
W198	Unspecified fall	Yes	No
W199	Unspecified fall	Yes	No
W20	Struck by thrown, projected or falling object	Yes	No
W200	Struck by thrown, projected or falling object	Yes	No

W201	Struck by thrown, projected or falling object	Yes	No
W202	Struck by thrown, projected or falling object	Yes	No
W203	Struck by thrown, projected or falling object	Yes	No
W204	Struck by thrown, projected or falling object	Yes	No
W205	Struck by thrown, projected or falling object	Yes	No
W206	Struck by thrown, projected or falling object	Yes	No
W207	Struck by thrown, projected or falling object	Yes	No
W208	Struck by thrown, projected or falling object	Yes	No
W209	Struck by thrown, projected or falling object	Yes	No
W21	Striking against or struck by sports equipment	Yes	No
W210	Striking against or struck by sports equipment	Yes	No
W211	Striking against or struck by sports equipment	Yes	No
W212	Striking against or struck by sports equipment	Yes	No
W213	Striking against or struck by sports equipment	Yes	No
W214	Striking against or struck by sports equipment	Yes	No
W215	Striking against or struck by sports equipment	Yes	No
W216	Striking against or struck by sports equipment	Yes	No
W217	Striking against or struck by sports equipment	Yes	No
W218	Striking against or struck by sports equipment	Yes	No
W219	Striking against or struck by sports equipment	Yes	No
W22	Striking against or struck by other objects	Yes	No
W220	Striking against or struck by other objects	Yes	No
W221	Striking against or struck by other objects	Yes	No
W222	Striking against or struck by other objects	Yes	No
W223	Striking against or struck by other objects	Yes	No
W224	Striking against or struck by other objects	Yes	No
W225	Striking against or struck by other objects	Yes	No
W226	Striking against or struck by other objects	Yes	No
W227	Striking against or struck by other objects	Yes	No
W228	Striking against or struck by other objects	Yes	No
W229	Striking against or struck by other objects	Yes	No
W23	Caught, crushed, jammed or pinched in or between objects	Yes	No
W230	Caught, crushed, jammed or pinched in or between objects	Yes	No
W231	Caught, crushed, jammed or pinched in or between objects	Yes	No
W232	Caught, crushed, jammed or pinched in or between objects	Yes	No
W233	Caught, crushed, jammed or pinched in or between objects	Yes	No
W234	Caught, crushed, jammed or pinched in or between objects	Yes	No
W235	Caught, crushed, jammed or pinched in or between objects	Yes	No
W236	Caught, crushed, jammed or pinched in or between objects	Yes	No
W237	Caught, crushed, jammed or pinched in or between objects	Yes	No
W238	Caught, crushed, jammed or pinched in or between objects	Yes	No
W239	Caught, crushed, jammed or pinched in or between objects	Yes	No
W24	Contact with lifting and transmission devices, not elsewhere cla	Yes	No
W240	Contact with lifting and transmission devices, not elsewhere cla	Yes	No
W241	Contact with lifting and transmission devices, not elsewhere cla	Yes	No
W242	Contact with lifting and transmission devices, not elsewhere cla	Yes	No
W243	Contact with lifting and transmission devices, not elsewhere cla	Yes	No
W244	Contact with lifting and transmission devices, not elsewhere cla	Yes	No
W245	Contact with lifting and transmission devices, not elsewhere cla	Yes	No
W246	Contact with lifting and transmission devices, not elsewhere cla	Yes	No
W247	Contact with lifting and transmission devices, not elsewhere cla	Yes	No
W248	Contact with lifting and transmission devices, not elsewhere cla	Yes	No
W249	Contact with lifting and transmission devices, not elsewhere cla	Yes	No

W25	Contact with sharp glass	Yes	No
W250	Contact with sharp glass	Yes	No
W251	Contact with sharp glass	Yes	No
W252	Contact with sharp glass	Yes	No
W253	Contact with sharp glass	Yes	No
W254	Contact with sharp glass	Yes	No
W255	Contact with sharp glass	Yes	No
W256	Contact with sharp glass	Yes	No
W257	Contact with sharp glass	Yes	No
W258	Contact with sharp glass	Yes	No
W259	Contact with sharp glass	Yes	No
W26	Contact with knife, sword or dagger	Yes	No
W260	Contact with knife, sword or dagger	Yes	No
W261	Contact with knife, sword or dagger	Yes	No
W262	Contact with knife, sword or dagger	Yes	No
W263	Contact with knife, sword or dagger	Yes	No
W264	Contact with knife, sword or dagger	Yes	No
W265	Contact with knife, sword or dagger	Yes	No
W266	Contact with knife, sword or dagger	Yes	No
W267	Contact with knife, sword or dagger	Yes	No
W268	Contact with knife, sword or dagger	Yes	No
W269	Contact with knife, sword or dagger	Yes	No
W27	Contact with nonpowered hand tool	Yes	No
W270	Contact with nonpowered hand tool	Yes	No
W271	Contact with nonpowered hand tool	Yes	No
W272	Contact with nonpowered hand tool	Yes	No
W273	Contact with nonpowered hand tool	Yes	No
W274	Contact with nonpowered hand tool	Yes	No
W275	Contact with nonpowered hand tool	Yes	No
W276	Contact with nonpowered hand tool	Yes	No
W277	Contact with nonpowered hand tool	Yes	No
W278	Contact with nonpowered hand tool	Yes	No
W279	Contact with nonpowered hand tool	Yes	No
W28	Contact with powered lawnmower	Yes	No
W280	Contact with powered lawnmower	Yes	No
W281	Contact with powered lawnmower	Yes	No
W282	Contact with powered lawnmower	Yes	No
W283	Contact with powered lawnmower	Yes	No
W284	Contact with powered lawnmower	Yes	No
W285	Contact with powered lawnmower	Yes	No
W286	Contact with powered lawnmower	Yes	No
W287	Contact with powered lawnmower	Yes	No
W288	Contact with powered lawnmower	Yes	No
W289	Contact with powered lawnmower	Yes	No
W29	Contact with other powered hand tools and household machin	Yes	No
W290	Contact with other powered hand tools and household machin	Yes	No
W291	Contact with other powered hand tools and household machin	Yes	No
W292	Contact with other powered hand tools and household machin	Yes	No
W293	Contact with other powered hand tools and household machin	Yes	No
W294	Contact with other powered hand tools and household machin	Yes	No
W295	Contact with other powered hand tools and household machin	Yes	No
W296	Contact with other powered hand tools and household machin	Yes	No
W297	Contact with other powered hand tools and household machin	Yes	No

W298	Contact with other powered hand tools and household machin	Yes	No
W299	Contact with other powered hand tools and household machin	Yes	No
W30	Contact with agricultural machinery	Yes	No
W300	Contact with agricultural machinery	Yes	No
W301	Contact with agricultural machinery	Yes	No
W302	Contact with agricultural machinery	Yes	No
W303	Contact with agricultural machinery	Yes	No
W304	Contact with agricultural machinery	Yes	No
W305	Contact with agricultural machinery	Yes	No
W306	Contact with agricultural machinery	Yes	No
W307	Contact with agricultural machinery	Yes	No
W308	Contact with agricultural machinery	Yes	No
W309	Contact with agricultural machinery	Yes	No
W31	Contact with other and unspecified machinery	Yes	No
W310	Contact with other and unspecified machinery	Yes	No
W311	Contact with other and unspecified machinery	Yes	No
W312	Contact with other and unspecified machinery	Yes	No
W313	Contact with other and unspecified machinery	Yes	No
W314	Contact with other and unspecified machinery	Yes	No
W315	Contact with other and unspecified machinery	Yes	No
W316	Contact with other and unspecified machinery	Yes	No
W317	Contact with other and unspecified machinery	Yes	No
W318	Contact with other and unspecified machinery	Yes	No
W319	Contact with other and unspecified machinery	Yes	No
W32	Handgun discharge	Yes	No
W320	Handgun discharge	Yes	No
W321	Handgun discharge	Yes	No
W322	Handgun discharge	Yes	No
W323	Handgun discharge	Yes	No
W324	Handgun discharge	Yes	No
W325	Handgun discharge	Yes	No
W326	Handgun discharge	Yes	No
W327	Handgun discharge	Yes	No
W328	Handgun discharge	Yes	No
W329	Handgun discharge	Yes	No
W33	Rifle, shotgun and larger firearm discharge	Yes	No
W330	Rifle, shotgun and larger firearm discharge	Yes	No
W331	Rifle, shotgun and larger firearm discharge	Yes	No
W332	Rifle, shotgun and larger firearm discharge	Yes	No
W333	Rifle, shotgun and larger firearm discharge	Yes	No
W334	Rifle, shotgun and larger firearm discharge	Yes	No
W335	Rifle, shotgun and larger firearm discharge	Yes	No
W336	Rifle, shotgun and larger firearm discharge	Yes	No
W337	Rifle, shotgun and larger firearm discharge	Yes	No
W338	Rifle, shotgun and larger firearm discharge	Yes	No
W339	Rifle, shotgun and larger firearm discharge	Yes	No
W34	Discharge from other and unspecified firearms	Yes	No
W340	Discharge from other and unspecified firearms	Yes	No
W341	Discharge from other and unspecified firearms	Yes	No
W342	Discharge from other and unspecified firearms	Yes	No
W343	Discharge from other and unspecified firearms	Yes	No
W344	Discharge from other and unspecified firearms	Yes	No
W345	Discharge from other and unspecified firearms	Yes	No

W346	Discharge from other and unspecified firearms	Yes	No
W347	Discharge from other and unspecified firearms	Yes	No
W348	Discharge from other and unspecified firearms	Yes	No
W349	Discharge from other and unspecified firearms	Yes	No
W35	Explosion and rupture of boiler	Yes	No
W350	Explosion and rupture of boiler	Yes	No
W351	Explosion and rupture of boiler	Yes	No
W352	Explosion and rupture of boiler	Yes	No
W353	Explosion and rupture of boiler	Yes	No
W354	Explosion and rupture of boiler	Yes	No
W355	Explosion and rupture of boiler	Yes	No
W356	Explosion and rupture of boiler	Yes	No
W357	Explosion and rupture of boiler	Yes	No
W358	Explosion and rupture of boiler	Yes	No
W359	Explosion and rupture of boiler	Yes	No
W36	Explosion and rupture of gas cylinder	Yes	No
W360	Explosion and rupture of gas cylinder	Yes	No
W361	Explosion and rupture of gas cylinder	Yes	No
W362	Explosion and rupture of gas cylinder	Yes	No
W363	Explosion and rupture of gas cylinder	Yes	No
W364	Explosion and rupture of gas cylinder	Yes	No
W365	Explosion and rupture of gas cylinder	Yes	No
W366	Explosion and rupture of gas cylinder	Yes	No
W367	Explosion and rupture of gas cylinder	Yes	No
W368	Explosion and rupture of gas cylinder	Yes	No
W369	Explosion and rupture of gas cylinder	Yes	No
W37	Explosion and rupture of pressurized tyre, pipe or hose	Yes	No
W370	Explosion and rupture of pressurized tyre, pipe or hose	Yes	No
W371	Explosion and rupture of pressurized tyre, pipe or hose	Yes	No
W372	Explosion and rupture of pressurized tyre, pipe or hose	Yes	No
W373	Explosion and rupture of pressurized tyre, pipe or hose	Yes	No
W374	Explosion and rupture of pressurized tyre, pipe or hose	Yes	No
W375	Explosion and rupture of pressurized tyre, pipe or hose	Yes	No
W376	Explosion and rupture of pressurized tyre, pipe or hose	Yes	No
W377	Explosion and rupture of pressurized tyre, pipe or hose	Yes	No
W378	Explosion and rupture of pressurized tyre, pipe or hose	Yes	No
W379	Explosion and rupture of pressurized tyre, pipe or hose	Yes	No
W38	Explosion and rupture of other specified pressurized devices	Yes	No
W380	Explosion and rupture of other specified pressurized devices	Yes	No
W381	Explosion and rupture of other specified pressurized devices	Yes	No
W382	Explosion and rupture of other specified pressurized devices	Yes	No
W383	Explosion and rupture of other specified pressurized devices	Yes	No
W384	Explosion and rupture of other specified pressurized devices	Yes	No
W385	Explosion and rupture of other specified pressurized devices	Yes	No
W386	Explosion and rupture of other specified pressurized devices	Yes	No
W387	Explosion and rupture of other specified pressurized devices	Yes	No
W388	Explosion and rupture of other specified pressurized devices	Yes	No
W389	Explosion and rupture of other specified pressurized devices	Yes	No
W39	Discharge of firework	Yes	No
W390	Discharge of firework	Yes	No
W391	Discharge of firework	Yes	No
W392	Discharge of firework	Yes	No
W393	Discharge of firework	Yes	No

W394	Discharge of firework	Yes	No
W395	Discharge of firework	Yes	No
W396	Discharge of firework	Yes	No
W397	Discharge of firework	Yes	No
W398	Discharge of firework	Yes	No
W399	Discharge of firework	Yes	No
W40	Explosion of other materials	Yes	No
W400	Explosion of other materials	Yes	No
W401	Explosion of other materials	Yes	No
W402	Explosion of other materials	Yes	No
W403	Explosion of other materials	Yes	No
W404	Explosion of other materials	Yes	No
W405	Explosion of other materials	Yes	No
W406	Explosion of other materials	Yes	No
W407	Explosion of other materials	Yes	No
W408	Explosion of other materials	Yes	No
W409	Explosion of other materials	Yes	No
W41	Exposure to high-pressure jet	Yes	No
W410	Exposure to high-pressure jet	Yes	No
W411	Exposure to high-pressure jet	Yes	No
W412	Exposure to high-pressure jet	Yes	No
W413	Exposure to high-pressure jet	Yes	No
W414	Exposure to high-pressure jet	Yes	No
W415	Exposure to high-pressure jet	Yes	No
W416	Exposure to high-pressure jet	Yes	No
W417	Exposure to high-pressure jet	Yes	No
W418	Exposure to high-pressure jet	Yes	No
W419	Exposure to high-pressure jet	Yes	No
W42	Exposure to noise	Yes	No
W420	Exposure to noise	Yes	No
W421	Exposure to noise	Yes	No
W422	Exposure to noise	Yes	No
W423	Exposure to noise	Yes	No
W424	Exposure to noise	Yes	No
W425	Exposure to noise	Yes	No
W426	Exposure to noise	Yes	No
W427	Exposure to noise	Yes	No
W428	Exposure to noise	Yes	No
W429	Exposure to noise	Yes	No
W43	Exposure to vibration	Yes	No
W430	Exposure to vibration	Yes	No
W431	Exposure to vibration	Yes	No
W432	Exposure to vibration	Yes	No
W433	Exposure to vibration	Yes	No
W434	Exposure to vibration	Yes	No
W435	Exposure to vibration	Yes	No
W436	Exposure to vibration	Yes	No
W437	Exposure to vibration	Yes	No
W438	Exposure to vibration	Yes	No
W439	Exposure to vibration	Yes	No
W44	Foreign body entering into or through eye or natural orifice	Yes	No
W440	Foreign body entering into or through eye or natural orifice	Yes	No
W441	Foreign body entering into or through eye or natural orifice	Yes	No

W442	Foreign body entering into or through eye or natural orifice	Yes	No
W443	Foreign body entering into or through eye or natural orifice	Yes	No
W444	Foreign body entering into or through eye or natural orifice	Yes	No
W445	Foreign body entering into or through eye or natural orifice	Yes	No
W446	Foreign body entering into or through eye or natural orifice	Yes	No
W447	Foreign body entering into or through eye or natural orifice	Yes	No
W448	Foreign body entering into or through eye or natural orifice	Yes	No
W449	Foreign body entering into or through eye or natural orifice	Yes	No
W45	Foreign body or object entering through skin	Yes	No
W450	Foreign body or object entering through skin	Yes	No
W451	Foreign body or object entering through skin	Yes	No
W452	Foreign body or object entering through skin	Yes	No
W453	Foreign body or object entering through skin	Yes	No
W454	Foreign body or object entering through skin	Yes	No
W455	Foreign body or object entering through skin	Yes	No
W456	Foreign body or object entering through skin	Yes	No
W457	Foreign body or object entering through skin	Yes	No
W458	Foreign body or object entering through skin	Yes	No
W459	Foreign body or object entering through skin	Yes	No
W46	Contact with hypodermic needle	Yes	No
W460	Contact with hypodermic needle	Yes	No
W461	Contact with hypodermic needle	Yes	No
W462	Contact with hypodermic needle	Yes	No
W463	Contact with hypodermic needle	Yes	No
W464	Contact with hypodermic needle	Yes	No
W465	Contact with hypodermic needle	Yes	No
W466	Contact with hypodermic needle	Yes	No
W467	Contact with hypodermic needle	Yes	No
W468	Contact with hypodermic needle	Yes	No
W469	Contact with hypodermic needle	Yes	No
W49	Exposure to other and unspecified inanimate mechanical forces	Yes	No
W490	Exposure to other and unspecified inanimate mechanical forces	Yes	No
W491	Exposure to other and unspecified inanimate mechanical forces	Yes	No
W492	Exposure to other and unspecified inanimate mechanical forces	Yes	No
W493	Exposure to other and unspecified inanimate mechanical forces	Yes	No
W494	Exposure to other and unspecified inanimate mechanical forces	Yes	No
W495	Exposure to other and unspecified inanimate mechanical forces	Yes	No
W496	Exposure to other and unspecified inanimate mechanical forces	Yes	No
W497	Exposure to other and unspecified inanimate mechanical forces	Yes	No
W498	Exposure to other and unspecified inanimate mechanical forces	Yes	No
W499	Exposure to other and unspecified inanimate mechanical forces	Yes	No
W50	Hit, struck, kicked, twisted, bitten or scratched by another pers	Yes	No
W500	Hit, struck, kicked, twisted, bitten or scratched by another pers	Yes	No
W501	Hit, struck, kicked, twisted, bitten or scratched by another pers	Yes	No
W502	Hit, struck, kicked, twisted, bitten or scratched by another pers	Yes	No
W503	Hit, struck, kicked, twisted, bitten or scratched by another pers	Yes	No
W504	Hit, struck, kicked, twisted, bitten or scratched by another pers	Yes	No
W505	Hit, struck, kicked, twisted, bitten or scratched by another pers	Yes	No
W506	Hit, struck, kicked, twisted, bitten or scratched by another pers	Yes	No
W507	Hit, struck, kicked, twisted, bitten or scratched by another pers	Yes	No
W508	Hit, struck, kicked, twisted, bitten or scratched by another pers	Yes	No
W509	Hit, struck, kicked, twisted, bitten or scratched by another pers	Yes	No
W51	Striking against or bumped into by another person	Yes	No

W510	Striking against or bumped into by another person	Yes	No
W511	Striking against or bumped into by another person	Yes	No
W512	Striking against or bumped into by another person	Yes	No
W513	Striking against or bumped into by another person	Yes	No
W514	Striking against or bumped into by another person	Yes	No
W515	Striking against or bumped into by another person	Yes	No
W516	Striking against or bumped into by another person	Yes	No
W517	Striking against or bumped into by another person	Yes	No
W518	Striking against or bumped into by another person	Yes	No
W519	Striking against or bumped into by another person	Yes	No
W53	Bitten by rat	Yes	No
W530	Bitten by rat	Yes	No
W531	Bitten by rat	Yes	No
W532	Bitten by rat	Yes	No
W533	Bitten by rat	Yes	No
W534	Bitten by rat	Yes	No
W535	Bitten by rat	Yes	No
W536	Bitten by rat	Yes	No
W537	Bitten by rat	Yes	No
W538	Bitten by rat	Yes	No
W539	Bitten by rat	Yes	No
W54	Bitten or struck by dog	Yes	No
W540	Bitten or struck by dog	Yes	No
W541	Bitten or struck by dog	Yes	No
W542	Bitten or struck by dog	Yes	No
W543	Bitten or struck by dog	Yes	No
W544	Bitten or struck by dog	Yes	No
W545	Bitten or struck by dog	Yes	No
W546	Bitten or struck by dog	Yes	No
W547	Bitten or struck by dog	Yes	No
W548	Bitten or struck by dog	Yes	No
W549	Bitten or struck by dog	Yes	No
W55	Bitten or struck by other mammals	Yes	No
W550	Bitten or struck by other mammals	Yes	No
W551	Bitten or struck by other mammals	Yes	No
W552	Bitten or struck by other mammals	Yes	No
W553	Bitten or struck by other mammals	Yes	No
W554	Bitten or struck by other mammals	Yes	No
W555	Bitten or struck by other mammals	Yes	No
W556	Bitten or struck by other mammals	Yes	No
W557	Bitten or struck by other mammals	Yes	No
W558	Bitten or struck by other mammals	Yes	No
W559	Bitten or struck by other mammals	Yes	No
W56	Contact with marine animal	Yes	No
W560	Contact with marine animal	Yes	No
W561	Contact with marine animal	Yes	No
W562	Contact with marine animal	Yes	No
W563	Contact with marine animal	Yes	No
W564	Contact with marine animal	Yes	No
W565	Contact with marine animal	Yes	No
W566	Contact with marine animal	Yes	No
W567	Contact with marine animal	Yes	No
W568	Contact with marine animal	Yes	No

W569	Contact with marine animal	Yes	No
W57	Bitten or stung by nonvenomous insect and other nonvenomou	Yes	No
W570	Bitten or stung by nonvenomous insect and other nonvenomou	Yes	No
W571	Bitten or stung by nonvenomous insect and other nonvenomou	Yes	No
W572	Bitten or stung by nonvenomous insect and other nonvenomou	Yes	No
W573	Bitten or stung by nonvenomous insect and other nonvenomou	Yes	No
W574	Bitten or stung by nonvenomous insect and other nonvenomou	Yes	No
W575	Bitten or stung by nonvenomous insect and other nonvenomou	Yes	No
W576	Bitten or stung by nonvenomous insect and other nonvenomou	Yes	No
W577	Bitten or stung by nonvenomous insect and other nonvenomou	Yes	No
W578	Bitten or stung by nonvenomous insect and other nonvenomou	Yes	No
W579	Bitten or stung by nonvenomous insect and other nonvenomou	Yes	No
W58	Bitten or struck by crocodile or alligator	Yes	No
W580	Bitten or struck by crocodile or alligator	Yes	No
W581	Bitten or struck by crocodile or alligator	Yes	No
W582	Bitten or struck by crocodile or alligator	Yes	No
W583	Bitten or struck by crocodile or alligator	Yes	No
W584	Bitten or struck by crocodile or alligator	Yes	No
W585	Bitten or struck by crocodile or alligator	Yes	No
W586	Bitten or struck by crocodile or alligator	Yes	No
W587	Bitten or struck by crocodile or alligator	Yes	No
W588	Bitten or struck by crocodile or alligator	Yes	No
W589	Bitten or struck by crocodile or alligator	Yes	No
W59	Bitten or crushed by other reptiles	Yes	No
W590	Bitten or crushed by other reptiles	Yes	No
W591	Bitten or crushed by other reptiles	Yes	No
W592	Bitten or crushed by other reptiles	Yes	No
W593	Bitten or crushed by other reptiles	Yes	No
W594	Bitten or crushed by other reptiles	Yes	No
W595	Bitten or crushed by other reptiles	Yes	No
W596	Bitten or crushed by other reptiles	Yes	No
W597	Bitten or crushed by other reptiles	Yes	No
W598	Bitten or crushed by other reptiles	Yes	No
W599	Bitten or crushed by other reptiles	Yes	No
W60	Contact with plant thorns and spines and sharp leaves	Yes	No
W600	Contact with plant thorns and spines and sharp leaves	Yes	No
W601	Contact with plant thorns and spines and sharp leaves	Yes	No
W602	Contact with plant thorns and spines and sharp leaves	Yes	No
W603	Contact with plant thorns and spines and sharp leaves	Yes	No
W604	Contact with plant thorns and spines and sharp leaves	Yes	No
W605	Contact with plant thorns and spines and sharp leaves	Yes	No
W606	Contact with plant thorns and spines and sharp leaves	Yes	No
W607	Contact with plant thorns and spines and sharp leaves	Yes	No
W608	Contact with plant thorns and spines and sharp leaves	Yes	No
W609	Contact with plant thorns and spines and sharp leaves	Yes	No
W64	Exposure to other and unspecified animate mechanical forces	Yes	No
W640	Exposure to other and unspecified animate mechanical forces	Yes	No
W641	Exposure to other and unspecified animate mechanical forces	Yes	No
W642	Exposure to other and unspecified animate mechanical forces	Yes	No
W643	Exposure to other and unspecified animate mechanical forces	Yes	No
W644	Exposure to other and unspecified animate mechanical forces	Yes	No
W645	Exposure to other and unspecified animate mechanical forces	Yes	No
W646	Exposure to other and unspecified animate mechanical forces	Yes	No

W647	Exposure to other and unspecified animate mechanical forces	Yes	No
W648	Exposure to other and unspecified animate mechanical forces	Yes	No
W649	Exposure to other and unspecified animate mechanical forces	Yes	No
W66	Drowning and submersion following fall into bath-tub	Yes	No
W660	Drowning and submersion following fall into bath-tub	Yes	No
W661	Drowning and submersion following fall into bath-tub	Yes	No
W662	Drowning and submersion following fall into bath-tub	Yes	No
W663	Drowning and submersion following fall into bath-tub	Yes	No
W664	Drowning and submersion following fall into bath-tub	Yes	No
W665	Drowning and submersion following fall into bath-tub	Yes	No
W666	Drowning and submersion following fall into bath-tub	Yes	No
W667	Drowning and submersion following fall into bath-tub	Yes	No
W668	Drowning and submersion following fall into bath-tub	Yes	No
W669	Drowning and submersion following fall into bath-tub	Yes	No
W67	Drowning and submersion while in swimming-pool	Yes	No
W670	Drowning and submersion while in swimming-pool	Yes	No
W671	Drowning and submersion while in swimming-pool	Yes	No
W672	Drowning and submersion while in swimming-pool	Yes	No
W673	Drowning and submersion while in swimming-pool	Yes	No
W674	Drowning and submersion while in swimming-pool	Yes	No
W675	Drowning and submersion while in swimming-pool	Yes	No
W676	Drowning and submersion while in swimming-pool	Yes	No
W677	Drowning and submersion while in swimming-pool	Yes	No
W678	Drowning and submersion while in swimming-pool	Yes	No
W679	Drowning and submersion while in swimming-pool	Yes	No
W68	Drowning and submersion following fall into swimming-pool	Yes	No
W680	Drowning and submersion following fall into swimming-pool	Yes	No
W681	Drowning and submersion following fall into swimming-pool	Yes	No
W682	Drowning and submersion following fall into swimming-pool	Yes	No
W683	Drowning and submersion following fall into swimming-pool	Yes	No
W684	Drowning and submersion following fall into swimming-pool	Yes	No
W685	Drowning and submersion following fall into swimming-pool	Yes	No
W686	Drowning and submersion following fall into swimming-pool	Yes	No
W687	Drowning and submersion following fall into swimming-pool	Yes	No
W688	Drowning and submersion following fall into swimming-pool	Yes	No
W689	Drowning and submersion following fall into swimming-pool	Yes	No
W73	Other specified drowning and submersion	Yes	No
W730	Other specified drowning and submersion	Yes	No
W731	Other specified drowning and submersion	Yes	No
W732	Other specified drowning and submersion	Yes	No
W733	Other specified drowning and submersion	Yes	No
W734	Other specified drowning and submersion	Yes	No
W735	Other specified drowning and submersion	Yes	No
W736	Other specified drowning and submersion	Yes	No
W737	Other specified drowning and submersion	Yes	No
W738	Other specified drowning and submersion	Yes	No
W739	Other specified drowning and submersion	Yes	No
W74	Unspecified drowning and submersion	Yes	No
W740	Unspecified drowning and submersion	Yes	No
W741	Unspecified drowning and submersion	Yes	No
W742	Unspecified drowning and submersion	Yes	No
W743	Unspecified drowning and submersion	Yes	No
W744	Unspecified drowning and submersion	Yes	No

W745	Unspecified drowning and submersion	Yes	No
W746	Unspecified drowning and submersion	Yes	No
W747	Unspecified drowning and submersion	Yes	No
W748	Unspecified drowning and submersion	Yes	No
W749	Unspecified drowning and submersion	Yes	No
W75	Accidental suffocation and strangulation in bed	Yes	No
W750	Accidental suffocation and strangulation in bed	Yes	No
W751	Accidental suffocation and strangulation in bed	Yes	No
W752	Accidental suffocation and strangulation in bed	Yes	No
W753	Accidental suffocation and strangulation in bed	Yes	No
W754	Accidental suffocation and strangulation in bed	Yes	No
W755	Accidental suffocation and strangulation in bed	Yes	No
W756	Accidental suffocation and strangulation in bed	Yes	No
W757	Accidental suffocation and strangulation in bed	Yes	No
W758	Accidental suffocation and strangulation in bed	Yes	No
W759	Accidental suffocation and strangulation in bed	Yes	No
W76	Other accidental hanging and strangulation	Yes	No
W760	Other accidental hanging and strangulation	Yes	No
W761	Other accidental hanging and strangulation	Yes	No
W762	Other accidental hanging and strangulation	Yes	No
W763	Other accidental hanging and strangulation	Yes	No
W764	Other accidental hanging and strangulation	Yes	No
W765	Other accidental hanging and strangulation	Yes	No
W766	Other accidental hanging and strangulation	Yes	No
W767	Other accidental hanging and strangulation	Yes	No
W768	Other accidental hanging and strangulation	Yes	No
W769	Other accidental hanging and strangulation	Yes	No
W77	Threat to breathing due to cave-in, falling earth and other subs	Yes	No
W770	Threat to breathing due to cave-in, falling earth and other subs	Yes	No
W771	Threat to breathing due to cave-in, falling earth and other subs	Yes	No
W772	Threat to breathing due to cave-in, falling earth and other subs	Yes	No
W773	Threat to breathing due to cave-in, falling earth and other subs	Yes	No
W774	Threat to breathing due to cave-in, falling earth and other subs	Yes	No
W775	Threat to breathing due to cave-in, falling earth and other subs	Yes	No
W776	Threat to breathing due to cave-in, falling earth and other subs	Yes	No
W777	Threat to breathing due to cave-in, falling earth and other subs	Yes	No
W778	Threat to breathing due to cave-in, falling earth and other subs	Yes	No
W779	Threat to breathing due to cave-in, falling earth and other subs	Yes	No
W78	Inhalation of gastric contents	Yes	No
W780	Inhalation of gastric contents	Yes	No
W781	Inhalation of gastric contents	Yes	No
W782	Inhalation of gastric contents	Yes	No
W783	Inhalation of gastric contents	Yes	No
W784	Inhalation of gastric contents	Yes	No
W785	Inhalation of gastric contents	Yes	No
W786	Inhalation of gastric contents	Yes	No
W787	Inhalation of gastric contents	Yes	No
W788	Inhalation of gastric contents	Yes	No
W789	Inhalation of gastric contents	Yes	No
W79	Inhalation and ingestion of food causing obstruction of respirat	Yes	No
W790	Inhalation and ingestion of food causing obstruction of respirat	Yes	No
W791	Inhalation and ingestion of food causing obstruction of respirat	Yes	No
W792	Inhalation and ingestion of food causing obstruction of respirat	Yes	No

W793	Inhalation and ingestion of food causing obstruction of respirat	Yes	No
W794	Inhalation and ingestion of food causing obstruction of respirat	Yes	No
W795	Inhalation and ingestion of food causing obstruction of respirat	Yes	No
W796	Inhalation and ingestion of food causing obstruction of respirat	Yes	No
W797	Inhalation and ingestion of food causing obstruction of respirat	Yes	No
W798	Inhalation and ingestion of food causing obstruction of respirat	Yes	No
W799	Inhalation and ingestion of food causing obstruction of respirat	Yes	No
W80	Inhalation and ingestion of other objects causing obstruction o	Yes	No
W800	Inhalation and ingestion of other objects causing obstruction o	Yes	No
W801	Inhalation and ingestion of other objects causing obstruction o	Yes	No
W802	Inhalation and ingestion of other objects causing obstruction o	Yes	No
W803	Inhalation and ingestion of other objects causing obstruction o	Yes	No
W804	Inhalation and ingestion of other objects causing obstruction o	Yes	No
W805	Inhalation and ingestion of other objects causing obstruction o	Yes	No
W806	Inhalation and ingestion of other objects causing obstruction o	Yes	No
W807	Inhalation and ingestion of other objects causing obstruction o	Yes	No
W808	Inhalation and ingestion of other objects causing obstruction o	Yes	No
W809	Inhalation and ingestion of other objects causing obstruction o	Yes	No
W81	Confined to or trapped in a low-oxygen environment	Yes	No
W810	Confined to or trapped in a low-oxygen environment	Yes	No
W811	Confined to or trapped in a low-oxygen environment	Yes	No
W812	Confined to or trapped in a low-oxygen environment	Yes	No
W813	Confined to or trapped in a low-oxygen environment	Yes	No
W814	Confined to or trapped in a low-oxygen environment	Yes	No
W815	Confined to or trapped in a low-oxygen environment	Yes	No
W816	Confined to or trapped in a low-oxygen environment	Yes	No
W817	Confined to or trapped in a low-oxygen environment	Yes	No
W818	Confined to or trapped in a low-oxygen environment	Yes	No
W819	Confined to or trapped in a low-oxygen environment	Yes	No
W83	Other specified threats to breathing	Yes	No
W830	Other specified threats to breathing	Yes	No
W831	Other specified threats to breathing	Yes	No
W832	Other specified threats to breathing	Yes	No
W833	Other specified threats to breathing	Yes	No
W834	Other specified threats to breathing	Yes	No
W835	Other specified threats to breathing	Yes	No
W836	Other specified threats to breathing	Yes	No
W837	Other specified threats to breathing	Yes	No
W838	Other specified threats to breathing	Yes	No
W839	Other specified threats to breathing	Yes	No
W84	Unspecified threat to breathing	Yes	No
W840	Unspecified threat to breathing	Yes	No
W841	Unspecified threat to breathing	Yes	No
W842	Unspecified threat to breathing	Yes	No
W843	Unspecified threat to breathing	Yes	No
W844	Unspecified threat to breathing	Yes	No
W845	Unspecified threat to breathing	Yes	No
W846	Unspecified threat to breathing	Yes	No
W847	Unspecified threat to breathing	Yes	No
W848	Unspecified threat to breathing	Yes	No
W849	Unspecified threat to breathing	Yes	No
W85	Exposure to electric transmission lines	Yes	No
W850	Exposure to electric transmission lines	Yes	No

W851	Exposure to electric transmission lines	Yes	No
W852	Exposure to electric transmission lines	Yes	No
W853	Exposure to electric transmission lines	Yes	No
W854	Exposure to electric transmission lines	Yes	No
W855	Exposure to electric transmission lines	Yes	No
W856	Exposure to electric transmission lines	Yes	No
W857	Exposure to electric transmission lines	Yes	No
W858	Exposure to electric transmission lines	Yes	No
W859	Exposure to electric transmission lines	Yes	No
W86	Exposure to other specified electric current	Yes	No
W860	Exposure to other specified electric current	Yes	No
W861	Exposure to other specified electric current	Yes	No
W862	Exposure to other specified electric current	Yes	No
W863	Exposure to other specified electric current	Yes	No
W864	Exposure to other specified electric current	Yes	No
W865	Exposure to other specified electric current	Yes	No
W866	Exposure to other specified electric current	Yes	No
W867	Exposure to other specified electric current	Yes	No
W868	Exposure to other specified electric current	Yes	No
W869	Exposure to other specified electric current	Yes	No
W87	Exposure to unspecified electric current	Yes	No
W870	Exposure to unspecified electric current	Yes	No
W871	Exposure to unspecified electric current	Yes	No
W872	Exposure to unspecified electric current	Yes	No
W873	Exposure to unspecified electric current	Yes	No
W874	Exposure to unspecified electric current	Yes	No
W875	Exposure to unspecified electric current	Yes	No
W876	Exposure to unspecified electric current	Yes	No
W877	Exposure to unspecified electric current	Yes	No
W878	Exposure to unspecified electric current	Yes	No
W879	Exposure to unspecified electric current	Yes	No
W92	Exposure to excessive heat of man-made origin	Yes	No
W920	Exposure to excessive heat of man-made origin	Yes	No
W921	Exposure to excessive heat of man-made origin	Yes	No
W922	Exposure to excessive heat of man-made origin	Yes	No
W923	Exposure to excessive heat of man-made origin	Yes	No
W924	Exposure to excessive heat of man-made origin	Yes	No
W925	Exposure to excessive heat of man-made origin	Yes	No
W926	Exposure to excessive heat of man-made origin	Yes	No
W927	Exposure to excessive heat of man-made origin	Yes	No
W928	Exposure to excessive heat of man-made origin	Yes	No
W929	Exposure to excessive heat of man-made origin	Yes	No
W93	Exposure to excessive cold of man-made origin	Yes	No
W930	Exposure to excessive cold of man-made origin	Yes	No
W931	Exposure to excessive cold of man-made origin	Yes	No
W932	Exposure to excessive cold of man-made origin	Yes	No
W933	Exposure to excessive cold of man-made origin	Yes	No
W934	Exposure to excessive cold of man-made origin	Yes	No
W935	Exposure to excessive cold of man-made origin	Yes	No
W936	Exposure to excessive cold of man-made origin	Yes	No
W937	Exposure to excessive cold of man-made origin	Yes	No
W938	Exposure to excessive cold of man-made origin	Yes	No
W939	Exposure to excessive cold of man-made origin	Yes	No

W99	Exposure to other and unspecified man-made environmental fa	Yes	No
W990	Exposure to other and unspecified man-made environmental fa	Yes	No
W991	Exposure to other and unspecified man-made environmental fa	Yes	No
W992	Exposure to other and unspecified man-made environmental fa	Yes	No
W993	Exposure to other and unspecified man-made environmental fa	Yes	No
W994	Exposure to other and unspecified man-made environmental fa	Yes	No
W995	Exposure to other and unspecified man-made environmental fa	Yes	No
W996	Exposure to other and unspecified man-made environmental fa	Yes	No
W997	Exposure to other and unspecified man-made environmental fa	Yes	No
W998	Exposure to other and unspecified man-made environmental fa	Yes	No
W999	Exposure to other and unspecified man-made environmental fa	Yes	No
X00	Exposure to uncontrolled fire in building or structure	Yes	No
X000	Exposure to uncontrolled fire in building or structure	Yes	No
X001	Exposure to uncontrolled fire in building or structure	Yes	No
X002	Exposure to uncontrolled fire in building or structure	Yes	No
X003	Exposure to uncontrolled fire in building or structure	Yes	No
X004	Exposure to uncontrolled fire in building or structure	Yes	No
X005	Exposure to uncontrolled fire in building or structure	Yes	No
X006	Exposure to uncontrolled fire in building or structure	Yes	No
X007	Exposure to uncontrolled fire in building or structure	Yes	No
X008	Exposure to uncontrolled fire in building or structure	Yes	No
X009	Exposure to uncontrolled fire in building or structure	Yes	No
X01	Exposure to uncontrolled fire, not in building or structure	Yes	No
X010	Exposure to uncontrolled fire, not in building or structure	Yes	No
X011	Exposure to uncontrolled fire, not in building or structure	Yes	No
X012	Exposure to uncontrolled fire, not in building or structure	Yes	No
X013	Exposure to uncontrolled fire, not in building or structure	Yes	No
X014	Exposure to uncontrolled fire, not in building or structure	Yes	No
X015	Exposure to uncontrolled fire, not in building or structure	Yes	No
X016	Exposure to uncontrolled fire, not in building or structure	Yes	No
X017	Exposure to uncontrolled fire, not in building or structure	Yes	No
X018	Exposure to uncontrolled fire, not in building or structure	Yes	No
X019	Exposure to uncontrolled fire, not in building or structure	Yes	No
X03	Exposure to controlled fire, not in building or structure	Yes	No
X030	Exposure to controlled fire, not in building or structure	Yes	No
X031	Exposure to controlled fire, not in building or structure	Yes	No
X032	Exposure to controlled fire, not in building or structure	Yes	No
X033	Exposure to controlled fire, not in building or structure	Yes	No
X034	Exposure to controlled fire, not in building or structure	Yes	No
X035	Exposure to controlled fire, not in building or structure	Yes	No
X036	Exposure to controlled fire, not in building or structure	Yes	No
X037	Exposure to controlled fire, not in building or structure	Yes	No
X038	Exposure to controlled fire, not in building or structure	Yes	No
X039	Exposure to controlled fire, not in building or structure	Yes	No
X04	Exposure to ignition of highly flammable material	Yes	No
X040	Exposure to ignition of highly flammable material	Yes	No
X041	Exposure to ignition of highly flammable material	Yes	No
X042	Exposure to ignition of highly flammable material	Yes	No
X043	Exposure to ignition of highly flammable material	Yes	No
X044	Exposure to ignition of highly flammable material	Yes	No
X045	Exposure to ignition of highly flammable material	Yes	No
X046	Exposure to ignition of highly flammable material	Yes	No
X047	Exposure to ignition of highly flammable material	Yes	No

X048	Exposure to ignition of highly flammable material	Yes	No
X049	Exposure to ignition of highly flammable material	Yes	No
X05	Exposure to ignition or melting of nightwear	Yes	No
X050	Exposure to ignition or melting of nightwear	Yes	No
X051	Exposure to ignition or melting of nightwear	Yes	No
X052	Exposure to ignition or melting of nightwear	Yes	No
X053	Exposure to ignition or melting of nightwear	Yes	No
X054	Exposure to ignition or melting of nightwear	Yes	No
X055	Exposure to ignition or melting of nightwear	Yes	No
X056	Exposure to ignition or melting of nightwear	Yes	No
X057	Exposure to ignition or melting of nightwear	Yes	No
X058	Exposure to ignition or melting of nightwear	Yes	No
X059	Exposure to ignition or melting of nightwear	Yes	No
X06	Exposure to ignition or melting of other clothing and apparel	Yes	No
X060	Exposure to ignition or melting of other clothing and apparel	Yes	No
X061	Exposure to ignition or melting of other clothing and apparel	Yes	No
X062	Exposure to ignition or melting of other clothing and apparel	Yes	No
X063	Exposure to ignition or melting of other clothing and apparel	Yes	No
X064	Exposure to ignition or melting of other clothing and apparel	Yes	No
X065	Exposure to ignition or melting of other clothing and apparel	Yes	No
X066	Exposure to ignition or melting of other clothing and apparel	Yes	No
X067	Exposure to ignition or melting of other clothing and apparel	Yes	No
X068	Exposure to ignition or melting of other clothing and apparel	Yes	No
X069	Exposure to ignition or melting of other clothing and apparel	Yes	No
X08	Exposure to other specified smoke, fire and flames	Yes	No
X080	Exposure to other specified smoke, fire and flames	Yes	No
X081	Exposure to other specified smoke, fire and flames	Yes	No
X082	Exposure to other specified smoke, fire and flames	Yes	No
X083	Exposure to other specified smoke, fire and flames	Yes	No
X084	Exposure to other specified smoke, fire and flames	Yes	No
X085	Exposure to other specified smoke, fire and flames	Yes	No
X086	Exposure to other specified smoke, fire and flames	Yes	No
X087	Exposure to other specified smoke, fire and flames	Yes	No
X088	Exposure to other specified smoke, fire and flames	Yes	No
X089	Exposure to other specified smoke, fire and flames	Yes	No
X09	Exposure to unspecified smoke, fire and flames	Yes	No
X090	Exposure to unspecified smoke, fire and flames	Yes	No
X091	Exposure to unspecified smoke, fire and flames	Yes	No
X092	Exposure to unspecified smoke, fire and flames	Yes	No
X093	Exposure to unspecified smoke, fire and flames	Yes	No
X094	Exposure to unspecified smoke, fire and flames	Yes	No
X095	Exposure to unspecified smoke, fire and flames	Yes	No
X096	Exposure to unspecified smoke, fire and flames	Yes	No
X097	Exposure to unspecified smoke, fire and flames	Yes	No
X098	Exposure to unspecified smoke, fire and flames	Yes	No
X099	Exposure to unspecified smoke, fire and flames	Yes	No
X10	Contact with hot drinks, food, fats and cooking oils	Yes	No
X100	Contact with hot drinks, food, fats and cooking oils	Yes	No
X101	Contact with hot drinks, food, fats and cooking oils	Yes	No
X102	Contact with hot drinks, food, fats and cooking oils	Yes	No
X103	Contact with hot drinks, food, fats and cooking oils	Yes	No
X104	Contact with hot drinks, food, fats and cooking oils	Yes	No
X105	Contact with hot drinks, food, fats and cooking oils	Yes	No

X106	Contact with hot drinks, food, fats and cooking oils	Yes	No
X107	Contact with hot drinks, food, fats and cooking oils	Yes	No
X108	Contact with hot drinks, food, fats and cooking oils	Yes	No
X109	Contact with hot drinks, food, fats and cooking oils	Yes	No
X11	Contact with hot tap-water	Yes	No
X110	Contact with hot tap-water	Yes	No
X111	Contact with hot tap-water	Yes	No
X112	Contact with hot tap-water	Yes	No
X113	Contact with hot tap-water	Yes	No
X114	Contact with hot tap-water	Yes	No
X115	Contact with hot tap-water	Yes	No
X116	Contact with hot tap-water	Yes	No
X117	Contact with hot tap-water	Yes	No
X118	Contact with hot tap-water	Yes	No
X119	Contact with hot tap-water	Yes	No
X12	Contact with other hot fluids	Yes	No
X120	Contact with other hot fluids	Yes	No
X121	Contact with other hot fluids	Yes	No
X122	Contact with other hot fluids	Yes	No
X123	Contact with other hot fluids	Yes	No
X124	Contact with other hot fluids	Yes	No
X125	Contact with other hot fluids	Yes	No
X126	Contact with other hot fluids	Yes	No
X127	Contact with other hot fluids	Yes	No
X128	Contact with other hot fluids	Yes	No
X129	Contact with other hot fluids	Yes	No
X13	Contact with steam and hot vapours	Yes	No
X130	Contact with steam and hot vapours	Yes	No
X131	Contact with steam and hot vapours	Yes	No
X132	Contact with steam and hot vapours	Yes	No
X133	Contact with steam and hot vapours	Yes	No
X134	Contact with steam and hot vapours	Yes	No
X135	Contact with steam and hot vapours	Yes	No
X136	Contact with steam and hot vapours	Yes	No
X137	Contact with steam and hot vapours	Yes	No
X138	Contact with steam and hot vapours	Yes	No
X139	Contact with steam and hot vapours	Yes	No
X14	Contact with hot air and gases	Yes	No
X140	Contact with hot air and gases	Yes	No
X141	Contact with hot air and gases	Yes	No
X142	Contact with hot air and gases	Yes	No
X143	Contact with hot air and gases	Yes	No
X144	Contact with hot air and gases	Yes	No
X145	Contact with hot air and gases	Yes	No
X146	Contact with hot air and gases	Yes	No
X147	Contact with hot air and gases	Yes	No
X148	Contact with hot air and gases	Yes	No
X149	Contact with hot air and gases	Yes	No
X15	Contact with hot household appliances	Yes	No
X150	Contact with hot household appliances	Yes	No
X151	Contact with hot household appliances	Yes	No
X152	Contact with hot household appliances	Yes	No
X153	Contact with hot household appliances	Yes	No

X154	Contact with hot household appliances	Yes	No
X155	Contact with hot household appliances	Yes	No
X156	Contact with hot household appliances	Yes	No
X157	Contact with hot household appliances	Yes	No
X158	Contact with hot household appliances	Yes	No
X159	Contact with hot household appliances	Yes	No
X16	Contact with hot heating appliances, radiators and pipes	Yes	No
X160	Contact with hot heating appliances, radiators and pipes	Yes	No
X161	Contact with hot heating appliances, radiators and pipes	Yes	No
X162	Contact with hot heating appliances, radiators and pipes	Yes	No
X163	Contact with hot heating appliances, radiators and pipes	Yes	No
X164	Contact with hot heating appliances, radiators and pipes	Yes	No
X165	Contact with hot heating appliances, radiators and pipes	Yes	No
X166	Contact with hot heating appliances, radiators and pipes	Yes	No
X167	Contact with hot heating appliances, radiators and pipes	Yes	No
X168	Contact with hot heating appliances, radiators and pipes	Yes	No
X169	Contact with hot heating appliances, radiators and pipes	Yes	No
X17	Contact with hot engines, machinery and tools	Yes	No
X170	Contact with hot engines, machinery and tools	Yes	No
X171	Contact with hot engines, machinery and tools	Yes	No
X172	Contact with hot engines, machinery and tools	Yes	No
X173	Contact with hot engines, machinery and tools	Yes	No
X174	Contact with hot engines, machinery and tools	Yes	No
X175	Contact with hot engines, machinery and tools	Yes	No
X176	Contact with hot engines, machinery and tools	Yes	No
X177	Contact with hot engines, machinery and tools	Yes	No
X178	Contact with hot engines, machinery and tools	Yes	No
X179	Contact with hot engines, machinery and tools	Yes	No
X18	Contact with other hot metals	Yes	No
X180	Contact with other hot metals	Yes	No
X181	Contact with other hot metals	Yes	No
X182	Contact with other hot metals	Yes	No
X183	Contact with other hot metals	Yes	No
X184	Contact with other hot metals	Yes	No
X185	Contact with other hot metals	Yes	No
X186	Contact with other hot metals	Yes	No
X187	Contact with other hot metals	Yes	No
X188	Contact with other hot metals	Yes	No
X189	Contact with other hot metals	Yes	No
X19	Contact with other and unspecified heat and hot substances	Yes	No
X190	Contact with other and unspecified heat and hot substances	Yes	No
X191	Contact with other and unspecified heat and hot substances	Yes	No
X192	Contact with other and unspecified heat and hot substances	Yes	No
X193	Contact with other and unspecified heat and hot substances	Yes	No
X194	Contact with other and unspecified heat and hot substances	Yes	No
X195	Contact with other and unspecified heat and hot substances	Yes	No
X196	Contact with other and unspecified heat and hot substances	Yes	No
X197	Contact with other and unspecified heat and hot substances	Yes	No
X198	Contact with other and unspecified heat and hot substances	Yes	No
X199	Contact with other and unspecified heat and hot substances	Yes	No
X20	Contact with venomous snakes and lizards	Yes	No
X200	Contact with venomous snakes and lizards	Yes	No
X201	Contact with venomous snakes and lizards	Yes	No

X202	Contact with venomous snakes and lizards	Yes	No
X203	Contact with venomous snakes and lizards	Yes	No
X204	Contact with venomous snakes and lizards	Yes	No
X205	Contact with venomous snakes and lizards	Yes	No
X206	Contact with venomous snakes and lizards	Yes	No
X207	Contact with venomous snakes and lizards	Yes	No
X208	Contact with venomous snakes and lizards	Yes	No
X209	Contact with venomous snakes and lizards	Yes	No
X21	Contact with venomous spiders	Yes	No
X210	Contact with venomous spiders	Yes	No
X211	Contact with venomous spiders	Yes	No
X212	Contact with venomous spiders	Yes	No
X213	Contact with venomous spiders	Yes	No
X214	Contact with venomous spiders	Yes	No
X215	Contact with venomous spiders	Yes	No
X216	Contact with venomous spiders	Yes	No
X217	Contact with venomous spiders	Yes	No
X218	Contact with venomous spiders	Yes	No
X219	Contact with venomous spiders	Yes	No
X22	Contact with scorpions	Yes	No
X220	Contact with scorpions	Yes	No
X221	Contact with scorpions	Yes	No
X222	Contact with scorpions	Yes	No
X223	Contact with scorpions	Yes	No
X224	Contact with scorpions	Yes	No
X225	Contact with scorpions	Yes	No
X226	Contact with scorpions	Yes	No
X227	Contact with scorpions	Yes	No
X228	Contact with scorpions	Yes	No
X229	Contact with scorpions	Yes	No
X23	Contact with hornets, wasps and bees	Yes	No
X230	Contact with hornets, wasps and bees	Yes	No
X231	Contact with hornets, wasps and bees	Yes	No
X232	Contact with hornets, wasps and bees	Yes	No
X233	Contact with hornets, wasps and bees	Yes	No
X234	Contact with hornets, wasps and bees	Yes	No
X235	Contact with hornets, wasps and bees	Yes	No
X236	Contact with hornets, wasps and bees	Yes	No
X237	Contact with hornets, wasps and bees	Yes	No
X238	Contact with hornets, wasps and bees	Yes	No
X239	Contact with hornets, wasps and bees	Yes	No
X24	Contact with centipedes and venomous millipedes (tropical)	Yes	No
X240	Contact with centipedes and venomous millipedes (tropical)	Yes	No
X241	Contact with centipedes and venomous millipedes (tropical)	Yes	No
X242	Contact with centipedes and venomous millipedes (tropical)	Yes	No
X243	Contact with centipedes and venomous millipedes (tropical)	Yes	No
X244	Contact with centipedes and venomous millipedes (tropical)	Yes	No
X245	Contact with centipedes and venomous millipedes (tropical)	Yes	No
X246	Contact with centipedes and venomous millipedes (tropical)	Yes	No
X247	Contact with centipedes and venomous millipedes (tropical)	Yes	No
X248	Contact with centipedes and venomous millipedes (tropical)	Yes	No
X249	Contact with centipedes and venomous millipedes (tropical)	Yes	No
X25	Contact with other venomous arthropods	Yes	No

X250	Contact with other venomous arthropods	Yes	No
X251	Contact with other venomous arthropods	Yes	No
X252	Contact with other venomous arthropods	Yes	No
X253	Contact with other venomous arthropods	Yes	No
X254	Contact with other venomous arthropods	Yes	No
X255	Contact with other venomous arthropods	Yes	No
X256	Contact with other venomous arthropods	Yes	No
X257	Contact with other venomous arthropods	Yes	No
X258	Contact with other venomous arthropods	Yes	No
X259	Contact with other venomous arthropods	Yes	No
X26	Contact with venomous marine animals and plants	Yes	No
X260	Contact with venomous marine animals and plants	Yes	No
X261	Contact with venomous marine animals and plants	Yes	No
X262	Contact with venomous marine animals and plants	Yes	No
X263	Contact with venomous marine animals and plants	Yes	No
X264	Contact with venomous marine animals and plants	Yes	No
X265	Contact with venomous marine animals and plants	Yes	No
X266	Contact with venomous marine animals and plants	Yes	No
X267	Contact with venomous marine animals and plants	Yes	No
X268	Contact with venomous marine animals and plants	Yes	No
X269	Contact with venomous marine animals and plants	Yes	No
X27	Contact with other specified venomous animals	Yes	No
X270	Contact with other specified venomous animals	Yes	No
X271	Contact with other specified venomous animals	Yes	No
X272	Contact with other specified venomous animals	Yes	No
X273	Contact with other specified venomous animals	Yes	No
X274	Contact with other specified venomous animals	Yes	No
X275	Contact with other specified venomous animals	Yes	No
X276	Contact with other specified venomous animals	Yes	No
X277	Contact with other specified venomous animals	Yes	No
X278	Contact with other specified venomous animals	Yes	No
X279	Contact with other specified venomous animals	Yes	No
X28	Contact with other specified venomous plants	Yes	No
X280	Contact with other specified venomous plants	Yes	No
X281	Contact with other specified venomous plants	Yes	No
X282	Contact with other specified venomous plants	Yes	No
X283	Contact with other specified venomous plants	Yes	No
X284	Contact with other specified venomous plants	Yes	No
X285	Contact with other specified venomous plants	Yes	No
X286	Contact with other specified venomous plants	Yes	No
X287	Contact with other specified venomous plants	Yes	No
X288	Contact with other specified venomous plants	Yes	No
X289	Contact with other specified venomous plants	Yes	No
X29	Contact with unspecified venomous animal or plant	Yes	No
X290	Contact with unspecified venomous animal or plant	Yes	No
X291	Contact with unspecified venomous animal or plant	Yes	No
X292	Contact with unspecified venomous animal or plant	Yes	No
X293	Contact with unspecified venomous animal or plant	Yes	No
X294	Contact with unspecified venomous animal or plant	Yes	No
X295	Contact with unspecified venomous animal or plant	Yes	No
X296	Contact with unspecified venomous animal or plant	Yes	No
X297	Contact with unspecified venomous animal or plant	Yes	No
X298	Contact with unspecified venomous animal or plant	Yes	No

X299	Contact with unspecified venomous animal or plant	Yes	No
X30	Exposure to excessive natural heat	Yes	No
X300	Exposure to excessive natural heat	Yes	No
X301	Exposure to excessive natural heat	Yes	No
X302	Exposure to excessive natural heat	Yes	No
X303	Exposure to excessive natural heat	Yes	No
X304	Exposure to excessive natural heat	Yes	No
X305	Exposure to excessive natural heat	Yes	No
X306	Exposure to excessive natural heat	Yes	No
X307	Exposure to excessive natural heat	Yes	No
X308	Exposure to excessive natural heat	Yes	No
X309	Exposure to excessive natural heat	Yes	No
X31	Exposure to excessive natural cold	Yes	No
X310	Exposure to excessive natural cold	Yes	No
X311	Exposure to excessive natural cold	Yes	No
X312	Exposure to excessive natural cold	Yes	No
X313	Exposure to excessive natural cold	Yes	No
X314	Exposure to excessive natural cold	Yes	No
X315	Exposure to excessive natural cold	Yes	No
X316	Exposure to excessive natural cold	Yes	No
X317	Exposure to excessive natural cold	Yes	No
X318	Exposure to excessive natural cold	Yes	No
X319	Exposure to excessive natural cold	Yes	No
X32	Exposure to sunlight	Yes	No
X320	Exposure to sunlight	Yes	No
X321	Exposure to sunlight	Yes	No
X322	Exposure to sunlight	Yes	No
X323	Exposure to sunlight	Yes	No
X324	Exposure to sunlight	Yes	No
X325	Exposure to sunlight	Yes	No
X326	Exposure to sunlight	Yes	No
X327	Exposure to sunlight	Yes	No
X328	Exposure to sunlight	Yes	No
X329	Exposure to sunlight	Yes	No
X58	Exposure to other specified factors	Yes	No
X580	Exposure to other specified factors	Yes	No
X581	Exposure to other specified factors	Yes	No
X582	Exposure to other specified factors	Yes	No
X583	Exposure to other specified factors	Yes	No
X584	Exposure to other specified factors	Yes	No
X585	Exposure to other specified factors	Yes	No
X586	Exposure to other specified factors	Yes	No
X587	Exposure to other specified factors	Yes	No
X588	Exposure to other specified factors	Yes	No
X589	Exposure to other specified factors	Yes	No
X59	Exposure to unspecified factor	Yes	No
X590	Exposure to unspecified factor	Yes	No
X591	Exposure to unspecified factor	Yes	No
X592	Exposure to unspecified factor	Yes	No
X593	Exposure to unspecified factor	Yes	No
X594	Exposure to unspecified factor	Yes	No
X595	Exposure to unspecified factor	Yes	No
X596	Exposure to unspecified factor	Yes	No

X597	Exposure to unspecified factor	Yes	No
X598	Exposure to unspecified factor	Yes	No
X599	Exposure to unspecified factor	Yes	No
