# Supplementary material for: Impact of the national home safety equipment scheme ‘Safe At Home’ on hospital admissions for unintentional injury in children under 5: a controlled interrupted time series analysis
Source: J Epidemiol Community Health. 2021 Jun 22;76(1):53–9. doi: 10.1136/jech-2021-216613 (PMC8666806; doi:10.1136/jech-2021-216613)
Supplement: Supplementary data [file jech-2021-216613supp003.pdf]

Supplementary methods file

Analytical model design.
We used the approach in the tutorial paper by Bernal [1] to set up our regression model in the first instance.

We used a regression model equation that utilized the time elapsed in the study (T), measured in months from January 2004 to December 2015 and a binary indicator (G) to represent the intervention group (G=1) or control group (G=0). A term X was used to indicate the time period (e.g. the pre or post intervention period). Interaction terms between binary indicator variables and time were included to denote the changes in slopes from one time period of the study to another (such as when entering the implementation phase). These variables were not used to model step changes though, only slope (trend) changes from one time period to the next as the intervention was implemented over a period of time.

The basic regression model for a pre-intervention period and a single post-intervention time period with a control group, using the notation in Bernal (2018) [2] is:

$$Y_t = \beta_0 + \beta_1 T + \beta_2 TX_t + \beta_3 G + \beta_4 GT + \beta_5 GX_t T$$

Y_t is the outcome variable (injury admission rate) at time t, β_0 represents the intercept at T=0 in the control group, β_1 is the change in outcome per time unit increase in the control group (representing the underlying pre-intervention trend), β_2 indicates the change in slope in the control group in the post-intervention period (using the interaction between time and time period: TX_t) β_3 represents the difference in intercept at T=0 for the intervention group compared with the control group, β_4 represents the difference in slopes between the intervention and control group in the pre-intervention period and β_5 represents the difference between the changes in slopes in the control and intervention groups in the post-intervention period (3-way interaction term). Therefore β_5 is the main parameter of interest as it indicates whether there are differences in slopes between the intervention and control groups in the post-intervention period compared to the pre-intervention slopes.

In our full model we included additional interaction terms between the binary group variable (G), time period (X_t) and study time T, for each separate time period of the study (baseline, implementation phase, 1st post implementation phase, 2nd post implementation phase. The model therefore expanded to:

$$Y_t = \beta_0 + \beta_1 T + \beta_2 TX_1 + \beta_3 G + \beta_4 GT + \beta_5 GX_1 T + \beta_6 TX_2 + \beta_7 GX_2 T + \beta_8 TX_3 + \beta_9 GX_3 T$$

Where T = time in months from the beginning of the study to the end.
TX_1 = interaction term between the start of the SAH scheme and time. Essentially this counts time from the beginning of the start of the intervention implementation period. And similarly:
TX_2 = interaction term between the start of the 1st post intervention phase and time.
TX_3 = interaction term between the start of the 2nd post intervention phase and time.

The model coefficients β_5 , β_7 and β_9 are the main parameters of interest as they indicates whether there are differences in slopes between the intervention and control groups in each of the post-intervention time periods compared to pre-intervention slopes.

Our final model also included Fourier (sinusoidal) terms to model the seasonal variation in injury rates.

We used a zero truncated, negative binomial model (a variant of Poisson regression) due to the injury data being overdispersed and there being no zero counts. This method models the log of the injury rate, using the injury counts and the mid-year population estimate as an offset term, rather than the injury rate itself. So the above model becomes:

$\log{(Y_t)} = \beta_0 + \beta_1 T + \beta_2 TX_1 + \beta_3 G + \beta_4 GT + \beta_5 GX_1 T + \beta_6 TX_2 + \beta_7 GX_2 T + \beta_8 TX_3 + \beta_9 GX_3 T$ + sinusoidal terms for seasonal effects

Where the log of the mean injury rate is modelled as a linear combination of the covariates.

References

[1] Bernal JL, Cummins S, Gasparrini A: Interrupted time series regression for the evaluation of public health interventions: a tutorial. International Journal of Epidemiology 2017, 46(1):348-355.

[2] Bernal JL, Cummins S, Gasparrini A: The use of controls in interrupted time series studies of public health interventions. International Journal of Epidemiology 2018, 47(6):2082-2093.
